# Significant Zr isotope variations in single zircon grains recording magma evolution history

**DOI:** 10.1073/pnas.2002053117

**Published:** 2020-08-18

**Authors:** Jing-Liang Guo, Zaicong Wang, Wen Zhang, Frédéric Moynier, Dandan Cui, Zhaochu Hu, Mihai N. Ducea

**Affiliations:** ^a^State Key Laboratory of Geological Processes and Mineral Resources, School of Earth Sciences, China University of Geosciences, Wuhan 430074, China;; ^b^Department of Geosciences, University of Arizona, Tucson, AZ 85718;; ^c^Institut de Physique du Globe de Paris, Université de Paris, CNRS, 75238 Paris Cedex 05, France;; ^d^Faculty of Geology and Geophysics, University of Bucharest, 010041 Bucharest, Romania

**Keywords:** Zr isotopes, zircon, magma evolution, laser ablation MC-ICP-MS, crustal differentiation

## Abstract

Zircons widely occur in magmatic rocks and often display internal zonation finely recording the magmatic history. Here, we presented in situ high-precision (2SD <0.15‰ for δ^94^Zr) and high–spatial-resolution (20 µm) stable Zr isotope compositions of magmatic zircons in a suite of calc-alkaline plutonic rocks from the juvenile part of the Gangdese arc, southern Tibet. These zircon grains are internally zoned with Zr isotopically light cores and increasingly heavier rims. Our data suggest the preferential incorporation of lighter Zr isotopes in zircon from the melt, which would drive the residual melt to heavier values. The Rayleigh distillation model can well explain the observed internal zoning in single zircon grains, and the best-fit models gave average zircon–melt fractionation factors for each sample ranging from 0.99955 to 0.99988. The average fractionation factors are positively correlated with the median Ti-in-zircon temperatures, indicating a strong temperature dependence of Zr isotopic fractionation. The results demonstrate that in situ Zr isotope analyses would be another powerful contribution to the geochemical toolbox related to zircon. The findings of this study solve the fundamental issue on how zircon fractionates Zr isotopes in calc-alkaline magmas, the major type of magmas that led to forming continental crust over time. The results also show the great potential of stable Zr isotopes in tracing magmatic thermal and chemical evolution and thus possibly continental crustal differentiation.

## Significance

Zircon, a common accessory mineral in crustal rocks, records plentiful and critical information on the Earth’s history. The isotopes of its major component, Zr, could be another powerful but unexplored tracer. We apply high-precision, high–spatial-resolution, in situ laser ablation Zr isotope measurements of magmatic zircons in continental arc plutonic rocks. Single zircon grains show impressive internal zoning with lighter Zr isotopes in the core but heavier ones toward the rim, solving a fundamental but controversial issue on how zircon fractionates Zr isotope in evolving magmas. Our results also reveal a strong temperature dependence of Zr isotopic fractionation. The Zr isotope is thus very promising in deciphering the differentiation history of magmatic systems and possibly the continental crust through time.

Zircon (ZrSiO_4_) is one of the most important accessory minerals in rocks. It occurs in a large variety of lithologies and is generated primarily in intermediate to felsic igneous rocks (1, 2). Its trace element (e.g., Ti and rare-earth element [REE]) and isotopic (e.g., U–Pb, Lu–Hf, Si, and O) abundances have provided major information on the absolute timing, petrochronologic conditions, and tectonic backgrounds of important geological events along the Earth’s history (3–8). They are especially important for deciphering the growth and reworking history of the continental crust (9), the early differentiation of the Earth (4, 10), and the styles of plate tectonics through time (11, 12).

Magmatic zircons crystallize early in evolved calc-alkaline magmas (1). They are relatively insoluble in crustal melts and resistant to chemical and physical breakdown. The diffusion of major and trace elements in zircon is also sluggish (13). These characteristics allow zircon to preserve elemental and isotopic zoning, as well as several generations of geochemical information within a single grain (2, 13). Compared to bulk analyses, in situ analytical methods could extract more detailed thermal and chemical information from intragrain chemical zoning (14, 15). Since zircon has a significantly higher Zr concentration (∼48 wt% as a major element) than rock-forming minerals (none to a few hundred parts per million) (16), the Zr content of magma should be primarily controlled by zircon after its saturation. Given that Zr, as one of the high field strength elements, has long been used to monitor magmatic differentiation processes of the silicate Earth (17, 18), the Zr isotopes of zircon might also record related messages.

Recent developments of bulk and in situ analytical methods have permitted studies on mass-dependent Zr isotope fractionation in terrestrial samples, including zircons (15, 19–24). They show the great potential of Zr isotope in tracing magmatic processes. However, available data have been rather limited and resulted in very controversial interpretation (21, 22), such as for fractionation factor α (whether greater or smaller than 1). The α is defined as (^94^Zr/^90^Zr)_zircon_/(^94^Zr/^90^Zr)_melt_, which depicts how Zr isotopes partition into the crystallizing zircon and surrounding melt and is thus fundamental to understanding magmatic Zr isotope evolution. Inglis et al. (21) studied a highly differentiated magmatic suite from the Hekla volcano in Iceland, and suggested an α of 0.9995, given the increasing δ^94^Zr (the permil deviation of ^94^Zr/^90^Zr from the IPGP-Zr standard) along with decreasing Zr contents in highly evolved samples (SiO_2_ >65 wt%). It implies that zircon favors light Zr isotopes from the melt, consistent with theoretical predictions based on the Zr-coordination difference between zircon and the melt (21). On the other hand, zircon crystals extracted from FC1 gabbroic anorthosite (Duluth Complex, northeastern Minnesota) show extremely large Zr isotope variations (∼5‰), revealed by both in situ (15) and bulk measurements (22). The majority of bulk-zircon values are heavier than the bulk rock, and an α of 1.00106 was suggested accordingly (22). While these studies reached opposite conclusions, they both suggested that zircon crystallization would fractionate the Zr isotope composition of the melt. This effect might be recorded in the growth zoning of single zircon grains.

The internal growth zoning in magmatic zircons is common (2), and once formed, it could preserve information on the subtle and transient magmatic differentiation due to the negligible diffusion of rare earth and high-field strength elements (25). Thus, high–spatial-resolution in situ analyses of internally zoned zircon grains may provide key insights into magma differentiation. Here, we apply high-precision laser ablation multicollector inductively coupled plasma mass spectrometry (LA-MC-ICP-MS) and report in situ Zr isotope compositions of magmatic zircons from mafic to intermediate plutonic rocks, which represent the juvenile part of the Gangdese arc in southern Tibet (26, 27). The results reveal large internal Zr isotope variations in single grains, showing unambiguously the preference of light Zr isotope by zircons and set the base for wide future applications. This study further indicates a strong temperature influence on zircon–melt Zr isotope fractionation, suggesting its potential in depicting magmatic thermal and chemical evolution.

## Mafic–Intermediate Plutonic Rocks and Zircons from the Gangdese Arc, Southern Tibet

**Fig. 1.**
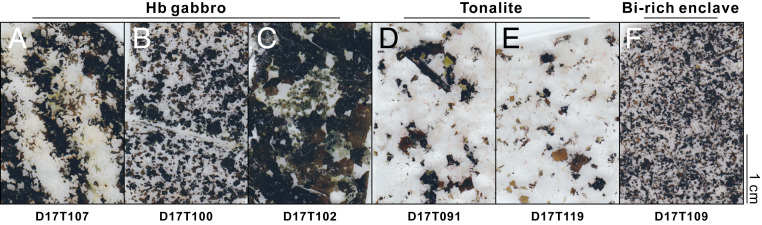
Scanned images of thick petrographic sections for plutonic rocks from the Gangdese arc, southern Tibet. (*A–C*) Hornblende gabbros. (*D* and *E*) Tonalites. (*F*) Biotite-rich enclave. Bi, biotite; Hb, hornblende.

The Gangdese arc in southern Tibet was produced by the northward subduction of the Neo-Tethyan oceanic lithosphere during the Mesozoic; this arc later witnessed the India–Asia collision during the Cenozoic (26, 28). Large volumes of Mesozoic plutonic rocks outcropped along the eastern segment of the Gangdese arc. These rocks typically show depleted mantle-like isotopic features, indicating significant juvenile crustal growth during this period (26). The studied samples belong to a suite of mafic–intermediate plutonic rocks collected from the Quxu batholith in the eastern Gangdese arc, including hornblende gabbros, tonalites, and biotite-rich microgranular enclaves hosted by the tonalites (Fig. 1 and *SI Appendix*, Table S1). These samples represent the juvenile part of Gangdese arc crust formed mainly by the fractional crystallization of arc magmas (27). Zirconium isotopes of the zircons may have recorded the differentiation of those calc-alkaline arc plutonic rocks. Here, we analyzed their U–Pb ages, major and trace element compositions, as well as Zr isotope compositions.

**Fig. 2.**
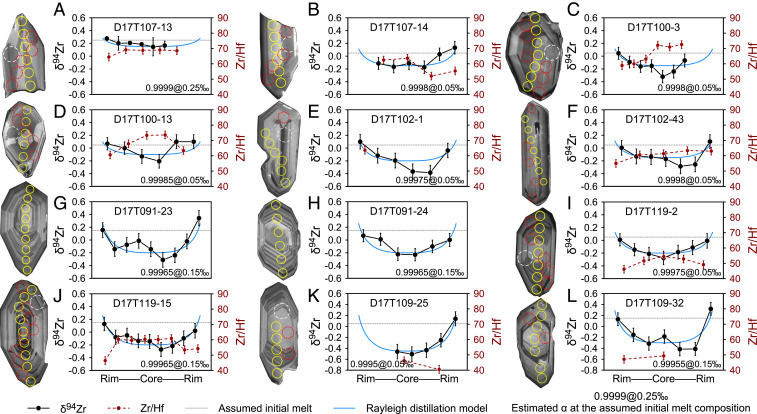
Representative Zr isotope and Zr/Hf profiles of magmatic zircon in plutonic rocks from the Gangdese arc, southern Tibet. (*A–F*) Hornblende gabbros. (*G–J*) Tonalites. (*K* and *L*) Biotite-rich enclave. The solid black and dashed red lines indicate δ^94^Zr and Zr/Hf profiles, respectively. The solid blue curves denote best-fit isotope profiles modeled from the Rayleigh distillation equation. The dotted gray lines indicate the initial melt composition used for modeling. Cathodoluminescence images of zircon are presented on the *Left*, showing the internal structures. The solid yellow, dotted red, and dashed white circles denote LA spots for Zr isotopes (20 μm in diameter), Zr/Hf profiles (24 μm in diameter), and U–Pb isotopes and trace elements (32 μm in diameter), respectively. Error bars for the δ^94^Zr and Zr/Hf ratios represent ±2SE uncertainties.

Zircons are rare in hornblende gabbros but are common in tonalites and biotite-rich enclaves. Most extracted grains are ∼100 to 300 μm in length with euhedral shapes. They vary in size and internal structure among different rock types (Fig. 2). Zircons from the hornblende gabbros are generally smaller and have linear, sector, or wide-banded oscillatory zoning (Fig. 2 *A*–*F*). In contrast, zircons from the tonalites and the biotite-rich enclave are larger and show dominantly oscillatory zoning with thin bands (Fig. 2 *G*–*J*). These features, together with high Th/U ratios (0.4 to 2.9) (*SI Appendix*, Table S2), indicate a magmatic origin (2). No systematic U–Pb age difference was found between the core and the rim. The emplacement ages of all samples range from ∼96 to ∼90 Ma (*SI Appendix*, Fig. S1 and Table S2), which are consistent with the magmatic flare-up (100 to 85 Ma) of regional arc magmatism (26).

The Zr isotope compositions of magmatic zircons were analyzed by femtosecond LA-MC-ICP-MS using a small spot size of 20 μm in diameter, which assured high spatial resolution and high precision (*SI Appendix*, Table S4). Isotope ratios were calibrated against zircon standard GJ-1 or Penglai, which are homogeneous in Zr isotope compositions shown by Zhang et al. (15). GJ-1 is a gem-quality megacryst zircon from Sri Lanka that has been widely used for U–Pb and Hf isotope analysis (29). The Penglai zircon is megacrysts (up to several millimeters in length) from Quaternary potassic basalts in south China used for O and Hf isotope analysis (30). In order to directly compare our zircon data with previous bulk analyses, the ^94^Zr/^90^Zr ratios were further expressed as the permil deviation from solution standard IPGP-Zr (Plasma-Cal standard solution; lot 5131203028). This standard has been used as a reference standard in most studies reporting Zr isotopic compositions and was recently cross-calibrated with different geological reference materials (15, 20, 21, 23). The within-run and long-term external precision for δ^94^Zr in this study are better than 0.10‰ (2SE) and 0.15‰ (2SD), respectively. The δ^94^Zr values of all analyzed zircons range from −0.86‰ to 0.41‰, with an average of −0.04 ± 0.39‰ (2SD, *n* = 237). The overall range (1.27‰) is larger than recently reported bulk rocks (0.582‰) (21), but smaller than that (∼5‰) observed in zircon and baddeleyite crystals from anorthosite FC1 (15, 22).

**Fig. 3.**
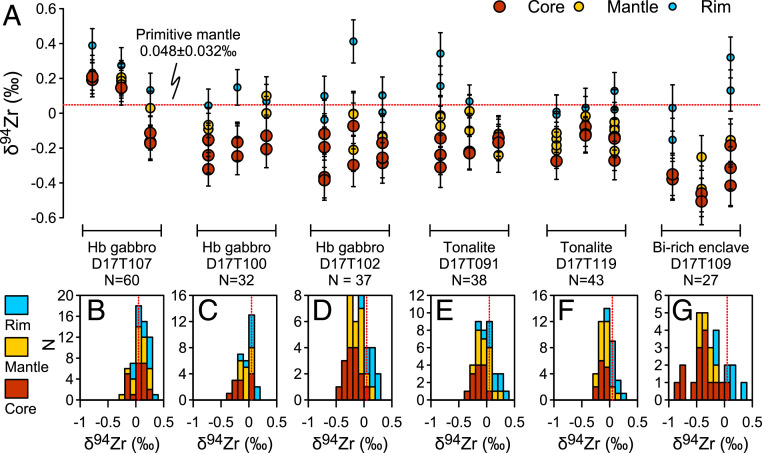
Intragrain core-to-rim Zr isotope variations in zircon from plutonic rocks in the Gangdese arc, southern Tibet. (*A*) Three representative zircon grains for each sample. (*B–G*) Histograms of δ^94^Zr values for the core, mantle, and rim of all zircon grains in each sample. *N* is the number of total analyses for each sample. The dotted red lines denote the primitive mantle value of 0.048 ± 0.032‰ (2SD) (21). Error bars represent ±2SE uncertainty.

Most zircon grains in this study show well-developed internal isotope zoning (Figs. 2 and 3). The intragrain variations can be as significant as 0.74‰, with an average of 0.31 ± 0.38‰ (2SD, *n* = 38) (*SI Appendix*, Table S4). The isotope profiles are characterized by low δ^94^Zr in the core and elevated values toward the rim (Figs. 2 and 3). The average δ^94^Zr value of zircon core for each sample shows a negative correlation with the relative core–rim difference (Δ_Rim–Core_) (*SI Appendix*, Table S1). A few zircon grains show complex zoning patterns with elevated or reduced δ^94^Zr values at the mantle relative to the core (e.g., Fig. 2 *G*, *J*, and *L*). Some of them show truncation surfaces in the core (e.g., Fig. 2*L*), indicating a possible antecrystic origin (inherited from previous magmatic pulses) given the indistinguishable ages (31).

Major and trace element profiles were also obtained along the Zr isotope profiles (*SI Appendix*, Table S5). The Zr/Hf ratios generally decrease from core to rim, opposite to δ^94^Zr (Fig. 2). This trend is clearer for zircons in samples with lower magmatic temperatures, and the slopes of δ^94^Zr vs. Zr/Hf for single zircon grains become steeper from hornblende gabbros to tonalites and the biotite-rich enclave (*SI Appendix*, Fig. S5). The Zr/Hf ratio of zircon has been used as an indicator for magmatic differentiation; zircons from more evolved melts tend to have lower Zr/Hf ratios (32). The broadly negative correlation between δ^94^Zr values and Zr/Hf ratios implies that the Zr isotope variations in zircon also records magmatic differentiation related to zircon crystallization.

The crystallization temperature of zircon was estimated using the Ti-in-zircon thermometer (33) (*SI Appendix*, Tables S2 and S5). This thermometer requires inputs of zircon Ti concentrations, bulk-rock SiO_2_ and TiO_2_ activities, and the pressure. The activities have a critical impact on the estimated temperatures (34), which were calculated using the thermodynamic software Perple_X (35) except for the biotite-rich enclave. This sample has quartz and rutile (inclusions in biotite), and thus the activities of SiO_2_ and TiO_2_ were both assigned to 1. The obtained temperatures were further corrected to 0.4 GPa (corresponding to a temperature increase of 30 °C) (33). The studied samples show systematic differences in the median Ti-in-zircon temperature: 743 to 831 °C for hornblende gabbros, 726 to 742 °C for tonalites, and 689 °C for the biotite-rich enclave (*SI Appendix*, Table S1). The temperature differences in different samples are consistent with their lithologies and petrographic textures (*SI Appendix*).

## Significance of Internal Zr Isotope Zoning in Single Zircon Grains

The most significant finding in this study is the large internal Zr isotope zoning (up to 0.74‰) in single zircon grains from the studied arc plutonic rocks (Figs. 2 and 3). Chemical zoning in minerals could be induced by growth or subsequent diffusion. The diffusivity of Zr in zircon was inferred from its geochemical twin Hf, which is almost immobile in zircon even at temperatures of 900 to 1,000 °C (25). Therefore, the Zr isotope zoning in zircon reflects most likely growth zoning, which may record the change of melt composition during magma differentiation.

Isotope profiles in these zircon grains are characterized by light cores and gradually heavier rims (Fig. 2). Most δ^94^Zr values of the zircon cores (as well as the average value of all analyses) are lower than that of the primitive mantle (0.048 ± 0.032‰) (21). Such zoning patterns indicate the preferential incorporation of light Zr isotopes into zircon (i.e., α < 1). Our finding is consistent with the first-order theoretical prediction where higher coordination-number lattices will be enriched in lighter isotopes, since in zircon, Zr has eightfold coordination (36), while in the melt it is generally found with sixfold coordination (37, 38). This is also consistent with the α of 0.9995 inferred from bulk analyses of the Hekla volcanic rocks, where rhyolites (SiO_2_ >65 wt%) show elevated δ^94^Zr values with reduced Zr contents (21). It is, however, different from the α of 1.00106 suggested from FC1 zircons in the Duluth anorthosite (15, 22). The FC1 zircons exhibit a much larger δ^94^Zr range of ∼5‰ (15, 22) than observed in megacryst zircons (<0.3‰) (15, 24) or fine- to medium-grained zircons in this study (1.27‰). The majority of FC1 zircons are isotopically heavier than the bulk rock by ∼0.3‰, resulting in α > 1 (22). However, the large range in FC1 was mainly caused by two low values (−2.530‰ and −4.278‰) in zircon fragments that were not treated by chemical abrasion (a method to selectively remove domains with high accumulated radiation damage) (22). If they were excluded, the range would drop to ∼1.8‰ (vs. 1.27‰ in this study). One anomalously low value of −3.44‰ also exists in in situ data, in contrast to the rest ranging from −0.84‰ to 1.49‰ (15). Radiation damage and later hydrothermal alteration were actually documented in anorthosite zircon AS3 from another outcrop in the Duluth Complex, resulting in depleted ZrO_2_ and SiO_2_ contents, discordant U–Pb ages, and enriched nonformula elements in metamict domains (39). Slight age discordance has also been reported for FC1 zircons (40). It is unclear whether metamictization (and later hydrothermal alteration) or the early crystallization of other Zr-bearing minerals have influenced the Zr isotope composition of FC1 zircons and the bulk rock. More work is needed to answer this question.

**Fig. 4.**
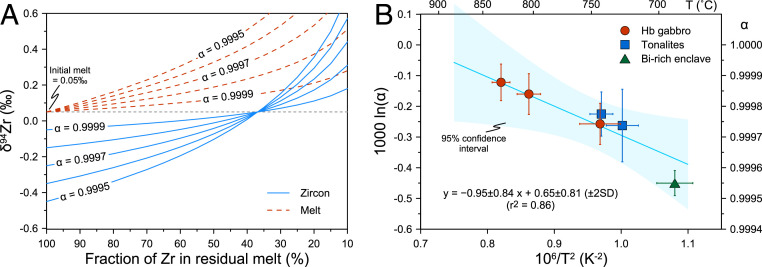
The fractionation mechanism of Zr isotopes between zircon and melt. (*A*) Rayleigh distillation modeling of Zr isotope evolution of crystallizing zircons and the residual melts. The solid and dashed lines denote zircon and melt compositions, respectively. The initial melt composition was assumed to be 0.05‰. (*B*) Correlation between average fractionation factor α and median Ti-in-zircon temperatures (T) for the studied samples. Fractionation factors were estimated from the best-fit Rayleigh distillation models. The overall trend indicates a strong temperature dependence, but large uncertainty exists in the regression, and cautions are needed when applying the obtained empiric equation. Error bars represent ±2SE uncertainty. The shaded area indicates the 95% confidence interval.

The internal zoning in zircon can provide more direct evidence and avoid some of the potential problems faced by bulk analyses (15, 24). The observed isotope profiles can be further modeled by Rayleigh distillation (Fig. 2 and *SI Appendix*, Text S6). This model assumes 1) equilibrium at the zircon–melt interface and 2) the melt is homogeneous, but once zircon is crystallized, it is considered to be isolated from the system. Assuming zircon as the only mineral controlling Zr isotope fractionation, the composition of crystallizing zircon and the residual melt (Fig. 4*A*) can be modeled using the following equations:

$$\delta^{94}Zr_{\mathrm{zircon}}=\;\left(\delta^{94}Zr_{\mathrm{melt},0}\;+\;1,000\right)\alpha \;F^{\left(\alpha -1\right)}-1,000,$$ [1]

$$\delta^{94}Zr_{\mathrm{melt}}=\;\left(\delta^{94}Zr_{\mathrm{melt},0}\;+\;1,000\right)\;F^{\left(\alpha -1\right)}-1,000,$$ [2]

where δ^94^Zr_melt,0_ is the initial melt isotopic composition, α is the zircon–melt isotopic fractionation factor, and F stands for the fraction of Zr content remaining in the melt. If α < 1, the crystallizing zircon is isotopically lighter than the surrounding melt; while zircon grows, the melt would evolve to heavier values, and so would the lately formed zircon rims (Fig. 4*A*). The Zr isotope profile in zircon can be further modeled by assuming sphere shapes for both zircons and the melt cell (24, 41). The radius of zircon grain would be proportional to the cubic root of 1 − F (i.e., the fraction of Zr consumed by zircon) (Fig. 2 and *SI Appendix*). More complex situations can be modeled by using a recently published MATLAB routine dealing with zircon dissolution and crystallization in complex magmatic systems (41).

The fractionation factor controls not only the δ^94^Zr difference between the crystallizing zircon and the surrounding melt, but also the shape of isotope profile (the closer the α is to 1, the flatter the profile is) (Fig. 2). Both factors were considered to determine the best-fit model, from which α can be estimated (Fig. 2). In our modeling, we avoided zircon cores that are inherited in origin. Different initial melt compositions (δ^94^Zr_melt,__0_ = 0.05‰, 0.15‰, and 0.25‰) were tested. The value of 0.05‰ was preferred unless higher values provided better-fitting curves for the observed isotope profiles, especially for those with overall heavy isotope compositions (Fig. 2 and *SI Appendix*, Table S6).

The assumption of a homogeneous melt for Zr in the Rayleigh distillation model may seem to be problematic, because its diffusivity (D) of Zr is low in the melt (42, 43) and kinetic isotope fractionation may take place in the diffusive boundary melt layer near zircon (44). Since light isotopes diffuse faster, they may lead to the observed isotopically light cores of zircon. However, the growth rate (R) of zircon is normally low (45, 46), which may reasonably result in R/D ratios <0.01 cm^−1^ and significant kinetic isotope fractionation is not expected unless R/D is much higher (44). Nevertheless, zircon growth rates and Zr diffusivities in the melt are not constant. If kinetic isotope effect was in control, the produced isotope profile in single zircon grain would possibly have a “W” shape as shown for trace elements based on a diffusion-controlled crystal growth model (44), being characterized by an isotopically heavy core, a light mantle, and a heavy rim (given a finite melt cell). The estimated α for zircons with W-shaped isotope profiles could be underestimated. However, zircon δ^94^Zr profiles in this study mostly exhibit simple U shapes, and only a few have W shapes (Fig. 2 *G* and *J*). This indicates that if kinetic isotope fractionation had existed, its role was limited. The observed isotope profiles are still best portraited by the Rayleigh distillation model as shown in Fig. 2.

The average α values for all six samples range from 0.99955 to 0.99988 (Fig. 2 and *SI Appendix*, Table S6), closer to 0.9995 inferred from Hekla volcanic rocks (21). The obtained values are all below 1, confirming the preference of light Zr isotopes in zircon. Moreover, α varies systematically among the hornblende gabbros (0.99974 to 0.99988), tonalites (0.99974 to 0.99978), and biotite-rich enclave (0.99955) (Fig. 4*B*). This suggests that the Zr isotope evolution of magmatic systems is a dynamic process, and a constant α may not be able to fully describe this process.

## Temperature Dependence of Zr Isotope Fractionation

As shown above, the zircon–melt fractionation factor α may vary in magmas of different temperatures and compositions. It is important to assess the potential controlling factor(s) on α. As predicted by classic stable isotope fractionation theories (47), mass-dependent fractionation should have a strong temperature dependence. At high-temperature conditions, the relationship between α for thermodynamic fractionation and the temperature (T, in kelvin) is described as follows:

$$1,000\;\ln\left(\alpha \right)\;=\;A\;\times \;10^{6}/T^{2}+\;B,$$ [3]

where A and B are equilibrium constants, and B should be 0 since fractionation approaches 0‰ at infinitely high temperatures. We use the average α value and median Ti-in-zircon temperature of zircon to evaluate the temperature dependence (Fig. 4*B*). The correlation (square of the correlation coefficient, *r*^2^ = 0.86) between 1,000 ln(α) and 1/T^2^ (T in kelvin) is obtained as follows:

$$1,000\;\ln\left(\alpha \right)\;=\;–0.95\pm 0.84\times 10^{6}/T^{2}+0.65\pm 0.81.$$ [4]

The correlation relies on the estimated fractionation factor, and thus the shape of Zr isotope profiles and the assumed initial melt compositions. The nonzero free term B is mostly caused by uncertainties in our data (the sample size is not large enough at present) and in the formula extrapolation. Less likely, it may be induced by zircon formation at nonequilibrium conditions for samples with low magmatic temperatures. Even if the latter were the case, the overall condition should be still close to equilibrium, as indicated by the U-shaped isotope profiles in most zircon grains. Given the large uncertainty in the regression, this empiric Eq. **4** so far cannot be applied to estimate precise temperature, which requires future calibration. Nonetheless, the good correlation (*r*^2^ = 0.86) itself still indicates a strong temperature dependence of zircon–melt Zr isotope fractionation.

The empirical Eq. **4** indicates that α will drop from 0.99983 to 0.99965 when the temperature decreases from 800 to 700 °C. At temperatures >850 °C, limited isotope fractionation (<0.1‰) is expected, whereas at lower temperatures, fractionation is supposed to be more significant especially for felsic magmas that may reach early zircon saturation. This equation also implies the potential use of simple zircon Zr isotope zoning as a tracer for changes in magma temperatures. Another application would be the estimation of initial melt composition [δ^94^Zr_melt,__0_ = δ^94^Zr_zircon,_ _core_ − 1,000 ln(α), and it is also noted that the rim composition is generally close to the assumed initial melt in Fig. 2] if the temperature can be constrained by other thermometers (Fig. 4*B*), which may be applicable to trace magmatic Zr isotope evolution.

## Tracing Magmatic Evolution History and Continental Crustal Differentiation

**Fig. 5.**
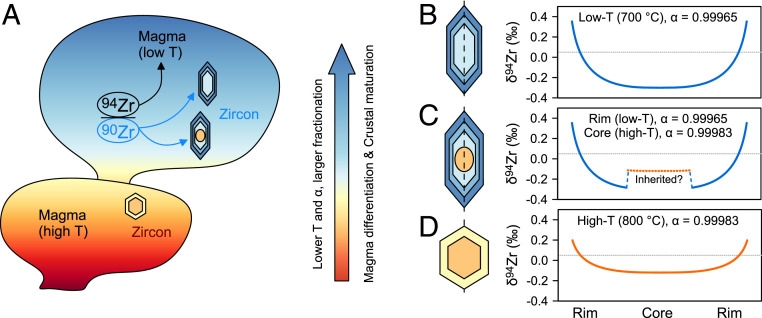
Schematic of zircon-induced Zr isotope fractionation during magma differentiation and examples of the produced Zr isotope profiles in single zircon grains, showing the potential of Zr isotopes in tracing the differentiation of magmatic systems and possibly the continental crust. The isotope profiles of zircon were modeled from the Rayleigh distillation equation, assuming an initial melt composition of 0.05‰ (dotted gray lines) with fractionation factor α calculated from Eq. **4** at given temperatures. (*A*) Light Zr isotopes in calc-alkaline magmas are preferentially incorporated by zircon, driving the residual melt to isotopically heavier compositions. Due to the strong temperature (T) dependence of zircon*–*melt Zr isotope fractionation, larger isotope fractionation is expected when magmas evolve to lower temperatures. (*B*) Modeled Zr isotope profile in zircon crystallized at 700 °C with a simple growth history. (*C*) Modeled Zr isotope profile in zircon with a complex growth history. The rim (solid blue line) and the core (dashed orange line) were calculated at temperatures of 700 and 800 °C, respectively. Note that complex zoning patterns may exist in natural zircons. (*D*) Modeled Zr isotope profile of zircon crystallized at 800 °C with a simple growth history.

Our study shows the importance of in situ zircon Zr isotope analyses in understanding the chemical and thermal evolution of calc-alkaline magmatic systems. These systems have played a major role in producing the continental crust (48). Magmatic zircon can preserve growth zoning of both Zr isotopes (Fig. 2) and trace elements (49) due to its low solubility in crustal melts, high resistance to chemical/physical breakdown, and low diffusivities of trivalent and tetravalent cations (e.g., REE, Ti, Zr, Hf, U, and Th) (25, 50). Unlike the trace element zoning that is controlled by multiple factors (such as the pressure and temperature, melt composition, coexisting minerals, and the growth rate of zircon) (49, 51), the Zr isotope zoning in magmatic zircons is less complex and mainly controlled by the melt composition and temperature (Figs. 4*B* and 5). Technically, it is also much easier to achieve high spatial resolution and high precision for LA in situ Zr isotope measurements as Zr is a major element of zircon with limited isotopic interferences (this study and ref. 15). For zircon grains with simple growth history (Fig. 5 *B* and *D*), their internal Zr isotope profiles can be used to trace changes in the magma temperature by applying the empirically calibrated correlation between zircon–melt α and the magma temperature. For zircons with relatively complex growth history (e.g., Fig. 5*C*), differences between anatectic cores and autocrystic rims may reveal additional information on the magmatic evolution history. For example, an anatectic core, with a truncation surface in CL images and a higher δ^94^Zr value, may have formed at a higher temperature than the rim (Fig. 5*C*). Together with other zircon-based geochemical tools, stable Zr isotopes would have wide applications in depicting magmatic processes such as magma assimilation and recharge or preeruption volcanic processes.

The internal isotope profile of single magmatic zircon further suggests that zircon preferentially incorporates light Zr isotopes, which means that the heavy isotopes would be concentrated in the residual melt. Zirconium, as an incompatible element during mantle melting and early magma differentiation (17, 18), is favored by the melt that eventually formed the continental crust of the Earth (48). Large fractionation among its isotopes is supposed to occur at late stages when the residual melts evolve to felsic compositions (>65 wt% SiO_2_) by liquid–crystal segregation (18, 21). Since zircon is generally unsaturated in oceanic basalts for their mafic (SiO_2_ ∼50 wt%), low-Zr (∼70 ppm), and high-temperature nature (52), large Zr isotope fractionation is not expected for the oceanic crust. Even if zircon exists, its crystallization temperature would probably be high and large Zr isotope fractionation is still not expected. This is consistent with the observed narrow variation range of δ^94^Zr (−0.033‰ to 0.086‰) in oceanic basalts (21). In comparison, the continental crust is characterized by an average andesitic composition (SiO_2_ = ∼60 wt%) (48) with more differentiated intermediate to felsic magmatic rocks. These rocks are easier to reach early zircon saturation than mafic ones (1). If the isotopically light zircon can be effectively removed, rocks like highly differentiated granites and rhyolites formed from the residual melt would have heavier Zr isotope compositions. The upper continental crust, where evolved magmas are concentrated, thus may have a heavier average Zr isotope composition than the primitive mantle. When the crust becomes more mature, the enrichment degree of heavy Zr isotopes in the upper crust would probably also increase. Thus, the Zr isotope composition of the upper crust in different continental regions may be used as an indicator for crustal maturation. Once confirmed, zircon Zr isotopes may open a new and insightful way to understand the evolution of the continental crust over geological time (from 4.4 billion years by Jack Hill zircons to present), particularly at the early stage of the first one or two billion years when the geological record but zircon has been relatively rare.

## Conclusions

We present in situ Zr isotope compositions of magmatic zircon in a calc-alkaline plutonic suite from the juvenile part of the Gangdese arc, southern Tibet. The Zr isotope data were obtained by LA-MC-ICP-MS with a high spatial resolution (20 µm) and high precision (2SD <0.15‰ for δ^94^Zr). The results show large variations ranging from −0.86‰ to 0.41‰, and most individual zircon grains exhibit internal zoning with low δ^94^Zr in the core and higher values toward the rim. This indicates that zircon favors light Zr isotopes from the melt, and its crystallization would drive melt to heavier Zr isotope compositions. This would set the base for future interpretation of the Zr isotope evolution in calc-alkaline igneous rocks, the major constituent of the continental crust. The observed isotope profiles in single zircon grains can be well explained by the Rayleigh distillation model. The best-fit models gave different average α values (0.99955 to 0.99988) for the studied samples, which are positively correlated with the median Ti-in-zircon temperatures, indicating a strong temperature dependence of zircon–melt Zr isotope fractionation. The results also demonstrate that in situ Zr isotope profiles in zircon could provide key insights into the chemical and thermal history of magmatic systems. The stable Zr isotope is thus a promising powerful tracer in revealing the differentiation of terrestrial magmatic systems and the Earth’s continental crust.

## Methods

The stable Zr isotopes of zircon were analyzed by using an NWRfemto femtosecond LA system (New Wave Research) coupled with a Neptune Plus MC-ICP-MS (Thermo Fisher Scientific) at the China University of Geosciences, Wuhan. The spot size was set to 20 μm in diameter. The ablation frequency was as low as 1 Hz, which reduced the risk of isotope fractionation induced by small-beam LA. Zircon U–Pb dating and trace element analyses were performed by using a 193-nm excimer ArF LA system (GeoLas HD; Coherent) coupled with Agilent 7700x quadrupole ICP-MS. Major and trace element compositions of zircon were further analyzed next to Zr isotope profiles by using GeoLas Pro 193-nm excimer ArF LA system coupled with Agilent 7700e ICP-MS at the Wuhan SampleSolution Analytical Technology Company. The CL images of zircon were taken to reveal the internal structure of zircon by using Analytical Scanning Electron Microscope JSM-IT100 (InTouchScope; JEOL) connected to a MINICL system (Gatan). The bulk-rock major and trace element compositions were analyzed by using X-ray fluorescence and Agilent 7900 quadrupole ICP-MS, respectively. Analytical details can be found in *SI Appendix*.

## Supplementary Material

Supplementary File

## Data Availability

All study data are included in *SI Appendix*.
