# Supplementary material for: Significant Zr isotope variations in single zircon grains recording magma evolution history
Source: Proc Natl Acad Sci U S A. 2020 Aug 18;117(35):21125–31. doi: 10.1073/pnas.2002053117 (PMC7474644; doi:10.1073/pnas.2002053117)
Supplement: Supplementary File [file pnas.2002053117.sapp.pdf]

PNAS
www.pnas.org

Supplementary Information for

Significant Zr isotope variations in single zircon grains recording magma evolution history

Jing-Liang Guo, Zaicong Wang, Wen Zhang, Frédéric Moynier, Dandan Cui, Zhaochu Hu, Mihai N Ducea

Jing-Liang Guo
Email: jl.guo@cug.edu.cn

Zaicong Wang
Email: zaicongwang@cug.edu.cn

This PDF file includes:

Supplementary text
Figures S1 to S5
SI References
Tables S1 to S9

www.pnas.org/cgi/doi/10.1073/pnas.2002053117

Supplementary Information Text

Text S1 LA-ICP-MS U–Pb dating of magmatic zircons

The zircon $^{206}Pb/^{238}U$ ages of all samples vary from 104.5 Ma to 87.0 Ma (Tables S2). Concordant zircons with age concordance of 90–110% (except for D17T119, 92–75%) were used to calculate the weighted mean $^{206}Pb/^{238}U$ age of each sample (Fig. S1). The weighted mean ages are identical to the Concordia U–Pb ages (if available) within errors (Fig. S1). We suggest the weighted mean $^{206}Pb/^{238}U$ ages to best represent the emplacement ages, which are 94.02±0.74 Ma (2σ, MSWD=1.19, n=9) for hornblende gabbro D17T107, 93.75±0.73 Ma (2σ, MSWD=7.1, n=10) for hornblende gabbro D17T100, 94.8±1.5 Ma (2σ, MSWD=0.11, n=15) for hornblende gabbro D17T102, 94.2±1.9 Ma (2σ, MSWD=7.4, n=17) for tonalite D17T091, 93.04±0.75 Ma (2σ, MSWD=1.08, n=17) for tonalite D17T119, and 96.2±1.3 Ma (2σ, MSWD=1.5, n=10) for biotite-rich enclave D17T109. All samples have comparable emplacement ages of ~96–93 Ma, which are within the magmatic flare-up of regional arc magmatism.

Text S2 Petrogenesis of the studied rocks from the Gangdese arc, southern Tibet

The hornblende gabbros mainly consist of hornblende + plagioclase ± clinopyroxene (Fig. 1 *A-C*). Medium- to coarse-grained samples (e.g., D17T102) show cumulate textures with hornblende ± clinopyroxene as the cumulate phase (Fig. 1 *C*). Some of the fine-grained samples (e.g., D17T107) are layered (Fig. 1 *A*). The variable P_2O_5 (up to 0.6 wt.%) and TiO_2 (up to 1.8 wt.%) contents (Fig. S2) further indicate possible accumulation of apatite and Ti-magnetite. Olivine is absent in these samples, implying that the magmas have already been evolved. The petrographic and geochemical features of the hornblende gabbros suggest that they are fractional crystallization products of arc magmas. The tonalites are small stocks or laccoliths intruding the hornblende gabbros. They mainly consist of feldspar + quartz + epidote + biotite with accessory minerals like apatite, magnetite, titanite, and zircon (Fig. 1 *D and E*). The absence of phenocrysts and the presence of magmatic epidote imply a slow cooling rate. The biotite-rich enclaves are hosted in the tonalites and have lengths of ~10–20 cm (Fig. 1 *F*). They are fine-grained and mainly consist of feldspar + biotite + quartz with trace amounts of calcite, zircon, and needle-like apatite. Rutile inclusions are present in biotite. The fine-grained texture and the presence of needle-like apatite indicate fast cooling. The biotite-rich enclaves have low SiO_2 and high K_2O contents, and are more enriched in rare earth elements than their host tonalites (Table S3). Their features are comparable to the biotite-rich schlieren or rind on mafic enclaves in felsic plutons (e.g., Bernasconi Hills Pluton in the Peninsular Ranges Batholith, southern California), which were formed by reactions between the solidified hornblende-bearing enclave and a hydrous K-rich residual melt or fluid when the host felsic magma had cooled to a low melt fraction state (1).

All studied samples are enriched in large ion lithophile elements and depleted in high field strength elements with insignificant Eu anomalies (Table S3). Late Cretaceous igneous rocks from the region show typical depleted mantle-like Sr–Nd isotope features (2). These features suggest the studied samples as part of the juvenile Gangdese arc crust, whose formation was related to the Late Cretaceous oceanic lithosphere subduction.

Text S3 Mass balance calculations for the modeled zircon radius vs. the Zr fraction of melt

The calculations assume 1) sphere shapes of zircon and the melt cell, and 2) Zr is highly incompatible (Kd ~ 0) in major-element minerals (nucleation of tiny zircon grains on the advancing surface of growing major-element minerals are not considered). For more complex situations, a recently published MATLAB software (3) is recommended. Given the same assumptions, comparable results were obtained using this software. The method used in this study is described below.

Parameters used in the calculation include:

Parameters for the melt cell		Parameters for the crystallizing zircon	
Radius	R	Radius	r
Volume	$V_{cell} = 4/3\ \pi\ R^3$	Volume	$V_{zrc} = 4/3\ \pi\ r^3$
Density	ρ_1	Density	ρ_2
Mass	$m_{cell} = V_{cell}\ \rho_1$	Mass	$m_{zrc} = V_{zrc}\ \rho_2$
Initial Zr concentration	C_{bulk}	Zr concentration	C_{zrc} = 480000 ppm
Fraction of Zr	F	Fraction of Zr	1−F
Total mass of Zr	m_{cell}<br>$= m\ C_{bulk}$<br>$= V_{cell}\ \rho_1\ C_{bulk}$<br>$= 4/3\ \pi\ R^3\ \rho_1\ C_{bulk}$	Total mass of Zr	m_{zrc}<br>$= m\ C_{zrc}$<br>$= V_{zrc}\ \rho_2\ C_{zrc}$<br>$= 4/3\ \pi\ r^3\ \rho_2\ C_{zrc}$

For mass balance, the total mass of Zr in zircon m_{zrc} should equal to m_{cell} (1−F), which is:

$$4/3\ \pi\ r^3\ \rho_2\ C_{zrc} = 4/3\ \pi\ R^3\ \rho_1\ C_{bulk}\ (1-F)$$

After reorganization, the radius of zircon is expressed as:

$$r = R \cdot (\rho_1/\rho_2 \cdot C_{bulk}/C_{zrc})^{1/3} \cdot (1-F)^{1/3}$$

Since R is user defined and C_{bulk}, C_{zrc}, ρ_1, and ρ_2 are considered to be constant, the radius of the precipitated zircon would be proportional to the cubic root of 1−F (i.e., the fraction of Zr consumed by zircon crystallization). The final radius of the modeled zircon grain could be adjusted to fit the observed size of zircons by changing the radius of the melt cell R.

Text S4 Analytical methods

Whole-rock geochemical analysis. Rock samples were crushed in a corundum jaw crusher and then powdered down to >200 mesh in an agate mill. Major and trace element compositions were analyzed at the Wuhan SampleSolution Analytical Technology Co., Ltd., Wuhan, China. The

major elements were measured by XRF (Rikagu RIX 2100) using fused glass disks. Precision and accuracy are better than 5%, as shown by analyses of USGS basalt and andesite standards (BCR-2, BHVO-2, and AGV-1). Trace elements were analyzed by Agilent 7900 ICP–MS. Sample powders were digested by HF + HNO_3 acid in Teflon bombs under high pressures (7–12 MPa). For most trace elements, the measured concentrations of BCR-2, BHVO-2, and AGV-1 agree with their reference values within a 5% difference. Duplicates of three samples were analyzed to test the analytical reproducibility, which is better than 5% for most elements.

Zircon separation and cathodoluminescence (CL) imaging. Zircons were separated from whole rocks by mechanical crushing, conventional magnetic and heavy-liquid separation methods. The hand specimens for zircon separation are typical ~10–20 cm in length. They commonly yielded several 100s or more zircon grains. At least 150 grains were hand-picked randomly and mounted in epoxy resin under a binocular microscope for most samples. The epoxy mounts were then polished to expose the center of zircons. Optical photomicrographs under both transmitted and reflected lights and CL images were documented to reveal the internal structure of zircons. Further analyses were carried out based on these images to avoid mineral inclusions, cracks, or altered zones. Zircon CL images were obtained by using an Analytical Scanning Electron Microscope JSM-IT100 (InTouchScope™, JEOL Ltd.) connected to a MINICL system (Gatan Inc.) at the Wuhan SampleSolution Analytical Technology Co., Ltd., China. The imaging condition was 10.0–13.0 kV voltage for the electric field and 80–85 μA current for the tungsten filament.

In-situ U–Pb isotope and trace element analyses of zircons by LA-ICP-MS. Zircon U–Pb dating and trace element analyses were simultaneously conducted by using a 193 nm excimer ArF laser ablation system (GeoLas HD, Coherent Inc., Göttingen, Germany) coupled with Agilent 7700x quadrupole inductively coupled plasma mass spectrometry (ICP–MS) at the State Key Laboratory of Geological Process and Mineral Resources, China University of Geosciences (Wuhan). Helium was applied as a carrier gas. Argon was used as the make-up gas and mixed with the carrier gas via a T-connector before entering the ICP. Two milliliters per minute of nitrogen were added to the central gas flow in LA-ICP-MS to increase the sensitivity of U–Th–Pb isotopes (4). A “wire” signal smoothing device is included in this laser ablation system (5). The carrier and make-up gas flows were optimized by ablating NIST SRM 610 to obtain the maximum signal intensity for ^{208}Pb, while keeping low ThO/Th and Ca^{2+}/Ca^{+} ratios to minimize the matrix-induced interference. The laser beam was set to 32 μm in diameter with a frequency of 6 Hz. The energy density was about 4 J/cm^2. Standard zircon 91500 (6) was used as an external standard for U–Pb dating. Zircon GJ-1 (7) was measured as the monitor standard. Both NIST SRM 610 and the standard zircons were used for trace element calibration. Reference values for Ti concentrations of zircon 91500 and GJ-1 are from refs. (8) and (9), respectively. Silicon was used

for internal standardization to reduce the matrix effect between synthetic glass and natural zircons (10). Each analysis incorporated a background acquisition of 20–30 s (gas blank) followed by 50 s data acquisition from the sample. Data reduction was performed using an Excel-based software ICPMSDataCal (11). Common Pb correction (12) was negligible for most zircons. Concordia diagrams and weighted averages were produced using Isoplot (ver. 4.15) (13). The obtained weighted average $^{206}Pb/^{238}U$ age is 600 ± 10 Ma (2SD, n = 32), consistent with its reference $^{206}Pb/^{238}U$ age of 599.8 ± 4.8 Ma (2σ) within analytical uncertainty (7). In-situ U–Pb isotope and Ti concentration data of sample and standard zircons are given in Table S2 and Table S8, respectively.

In-situ major and trace element profile analyses of zircons by LA-ICP-MS. Major and Trace element analyses of zircons were conducted by using a 193 nm excimer ArF laser ablation system (GeoLasPro, Coherent Inc., Göttingen, Germany) coupled with Agilent 7700e quadrupole inductively coupled plasma mass spectrometry (ICP–MS) at the Wuhan SampleSolution Analytical Technology Co., Ltd., Wuhan, China. Helium was applied as a carrier gas. Argon was used as the make-up gas and mixed with the carrier gas via a T-connector before entering the ICP. A “wire” signal smoothing device is included in this laser ablation system (5). In order to obtain a high spatial resolution without losing much of the precision, the spot size and frequency of the laser were set to 24 μm and 6 Hz, respectively. The analyzed spots were as close as possible to the spots for Zr isotope analyses. Element compositions were calibrated against various reference materials (NIST SRM 610, BHVO-2G, BCR-2G, and BIR-1G) (11). Standard zircon 91500 and GJ-1 were also analyzed as monitoring standards. Each analysis incorporated a background acquisition of approximately 20–30 s followed by 50 s sample data acquisition. An Excel-based software ICPMSDataCal was used for data reduction (11). The obtained major and trace element data of sample and standard zircons are given in Table S5.

In-situ Zr isotope analyses of zircons by fs-LA-MC-ICP-MS. In-situ Zr isotope analyses were performed by using a 257 nm Yb:YGB femtosecond laser ablation system (NWR FemtoUC, New Wave Research, U.S.A) connected to a multiple collector ICP–MS (Neptune Plus, Thermo Fisher Scientific, Bremen, Germany) at the State Key Laboratory of Geological Processes and Mineral Resources, China University of Geosciences (Wuhan). The Faraday collector configuration of the mass spectrometer was composed of an array from L4 to H2 to monitor $^{89}Y^+$, $^{90}Zr^+$, $^{91}Zr^+$, $^{92}Zr^+$, $^{94}Zr^+$, $^{95}Mo^+$, and $^{96}Zr^+$. A high-sensitivity combination of X-skimmer and Jet-sample cones was mounted in the Neptune Plus interface. The mass spectrometer was operated in the low mass resolution mode.

The laser ablation was conducted under a helium atmosphere, while argon was mixed into the sample-out line downstream from the ablation chamber before entering the torch of the mass

spectrometer. A two-volume laser-ablation cell (TwoVol2, New Wave Research, USA) was used to avoid the possible position effect. The output energy and the energy density of fs-LA-ICP-MS were set to 70% and 1.2 J cm^{-2}, respectively. The laser was set to a spot size of 20 μm with a low frequency of 1 Hz. Using the low ablation frequency is important to reduce the risk of isotope fractionation by the increased depth/diameter ratio of the ablation crater with time, especially for the small ablated spot size. A small amount of N_2 (8 ml min^{-1}) was added to the carrier gas flow behind a signal-smoothing device (5) via a simple Y-shaped connector. The signal-smoothing device was used downstream from the sample cell, which eliminated the short-term variability of the signal efficiently. Standard zircon GJ-1 was used to optimize the instrumental parameters, including the He and Ar gas flow rates, the torch position, the RF power, and the source lens setting. This helped to achieve suitable signal sensitivities and optimal peak shapes. Under these operating conditions, signal intensities of 8.7–18.6 V were achieved on the mass of ^{90}Zr for GJ-1. The background intensities of ^{90}Zr were below 0.001 V. Operating parameters for the laser system and MC-ICP-MS are summarized in Table S9.

The acquired data for each analysis consisted of 120 cycles (0.524 s integration time per cycle): the background was measured for the first 30 cycles, while the sample was measured for the remaining 90 cycles after the laser was turned on. The analytical sequence proceeded by bracketing 2–4 samples with the measurements of 1–2 standards. Standard zircon GJ-1 and Penglai were employed as the matrix-matching standards. The standard-sample bracketing technique was applied to correct the mass fractionation and instrument drift. The final Zr isotope ratios ($^{94}Zr/^{90}Zr$, $^{94}Zr/^{91}Zr$, and $^{96}Zr/^{90}Zr$) were calculated by correcting for instrumental mass fractionation using GJ-1 or Penglai. The Zr isotope compositions were expressed using the permil notation: $\delta^{9x/9y}Zr_{standard}$ (‰) = 1000 × [$(^{9x}Zr/^{9y}Zr)_{sample}/(^{9x}Zr/^{9y}Zr)_{standard}$ -1)], where x can be 4 and 6 and y can be 0 and 1. The obtained Zr isotope ratios were normalized to GJ-1 and IPGP-Zr, and only $\delta^{94/90}Zr$ values were given relative to IPGP-Zr. The IPGP-Zr is a standard solution used for double spike (^{91}Zr–^{96}Zr) MC-ICP-MS measurements of bulk samples in several previous studies (14–16). The within-run precision for one single-spot analysis was calculated by the combination of the SE (standard error) from both the samples and standards. Duplicate analyses of standard zircons GJ-1 and Penglai gave 2SD uncertainties of 0.07–0.09‰ for $\delta^{94/90}Zr_{GJ-1}$ (Fig. S3). By combining the internal and external uncertainties, most analyses of unknowns gave external uncertainties better than 0.15‰ (2SD) for $\delta^{94/90}Zr_{GJ-1}$. More details of instrumental operating conditions and data reduction procedures were described in ref. (16).

Signal intensities of 6.7–18.0 V on mass ^{90}Zr were achieved for unknown samples, which enable high-precision Zr isotope analyses with internal analytical uncertainties better than 0.05‰ (2SE) for $^{94}Zr/^{90}Zr$. The obtained $\delta^{94/90}Zr_{GJ-1}$, $\delta^{94/91}Zr_{GJ-1}$, and $\delta^{96/90}Zr_{GJ-1}$ of all samples are well correlated with each other (Fig. S4). The slopes (0.747 and 1.490, respectively) of regression

lines for $\delta^{94/91}Zr_{GJ-1}$ vs. $\delta^{94/90}Zr_{GJ-1}$ and $\delta^{96/90}Zr_{GJ-1}$ vs. $\delta^{94/90}Zr_{GJ-1}$ agree with the theoretical mass-dependent fractionation values of 0.746 and 1.484, respectively. This indicates the negligible influence of any isotope interference. The obtained $\delta^{94/90}Zr_{GJ-1}$ values of standard zircons GJ-1, Penglai, 91500, Plešovice, and zircon megacryst Zr-Paki as unknowns are consistent with their published values obtained by double spike MC-ICP-MS (15) and LA-MC-ICP-MS measurements (16) (Fig. S3 and Table S7).

Figures

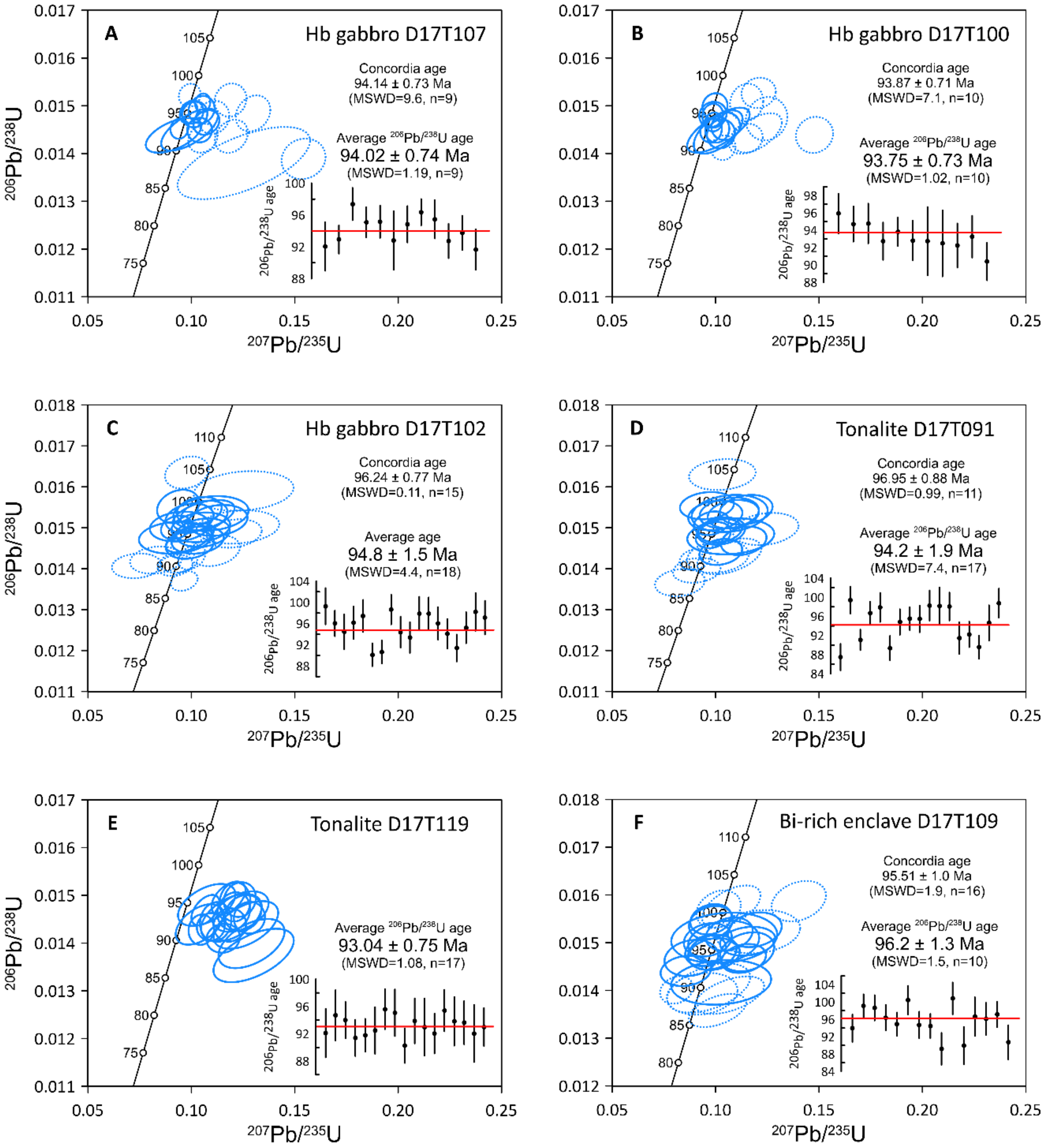

Fig. S1. Zircon U–Pb Concordia diagrams for plutonic arc rocks from southern Tibet. Data-point error ellipses are plotted at 1σ level. Solid and dashed ellipses denote analyses that were included and excluded from the calculation of concordant ages, respectively. Insets show $^{206}Pb/^{238}U$ ages (in Ma) of analyses with age concordance of 90–110% except for tonalite D17T119 (75–92%). The calculated ages and error bars in the insets are given at ±2σ level.

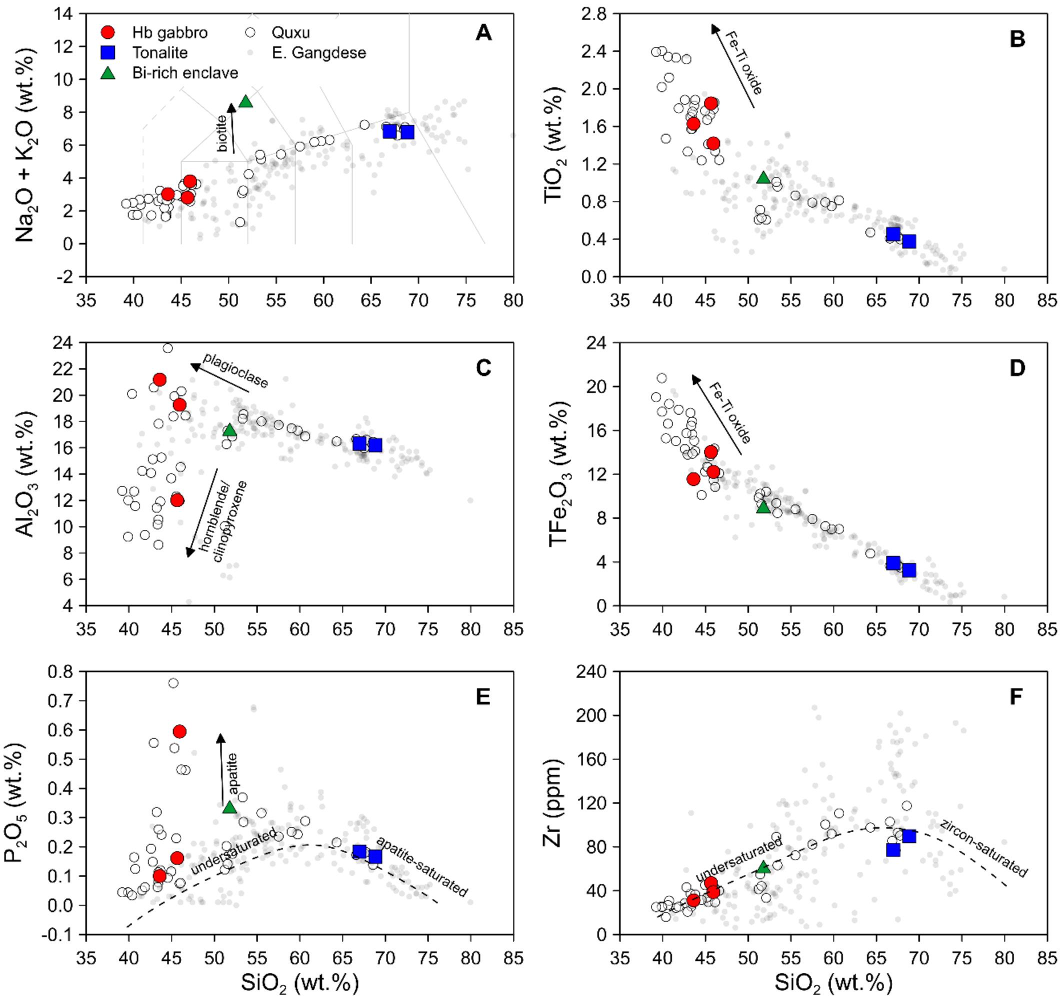

Fig. S2. Harker diagrams of plutonic arc rocks from southern Tibet. (*A*) Total alkaline contents ($Na_2O + K_2O$). (*B*) TiO_2. (*C*) Al_2O_3. (*D*) Total ion as Fe_2O_3. (*E*) P_2O_5. (*F*) Zr. Filled symbols indicate samples in this study. Open circles denote late Cretaceous igneous samples from the study area. Small dots denote late Cretaceous igneous rocks from the eastern Gangdese arc (17).

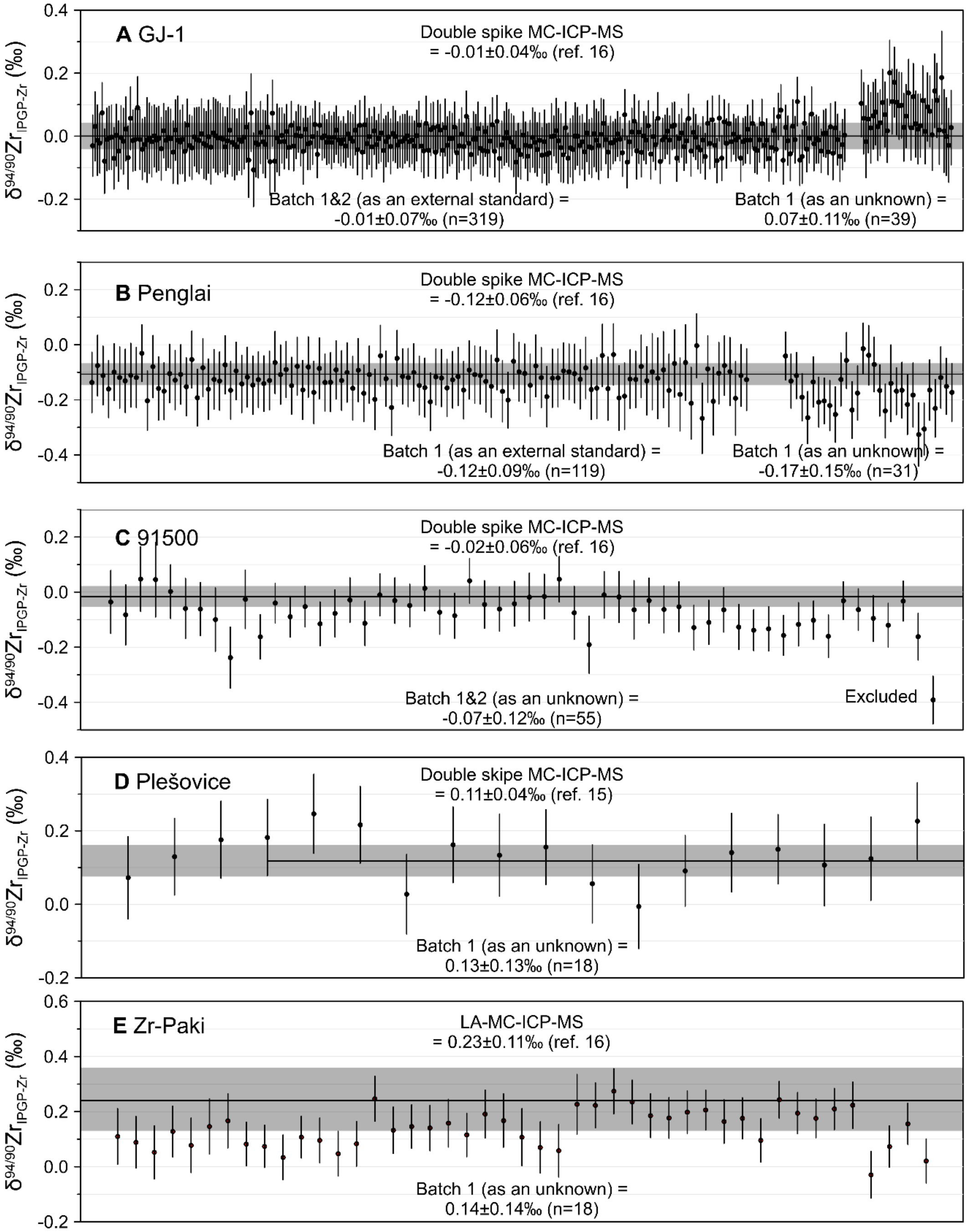

Fig. S3. Repeated measurements of δ^{94}Zr of standard zircons in three analytical sessions. (*A*) GJ-1. (*B*) Penglai. (*C*) 91500. (*D*) Plešovice**.** (*E*) Zr-Paki. Error bars are given at the ±2SE level. The solid lines and grey areas denote the reference values with ±2SD uncertainties determined by double spike MC-ICP-MS (15) or LA-MC-ICP-MS (16).

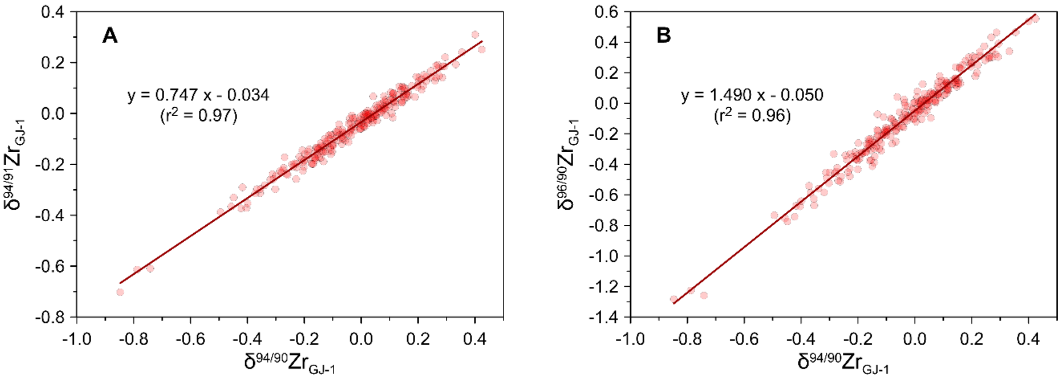

Fig. S4. The multiple-isotope plot of Zr isotopic compositions of zircons in plutonic arc rocks from southern Tibet. (*A*) $\delta^{94/91}Zr_{GJ\text{-}1}$ vs. $\delta^{94/90}Zr_{GJ\text{-}1}$. (*B*) $\delta^{96/90}Zr_{GJ\text{-}1}$ vs. $\delta^{94/90}Zr_{GJ\text{-}1}$.

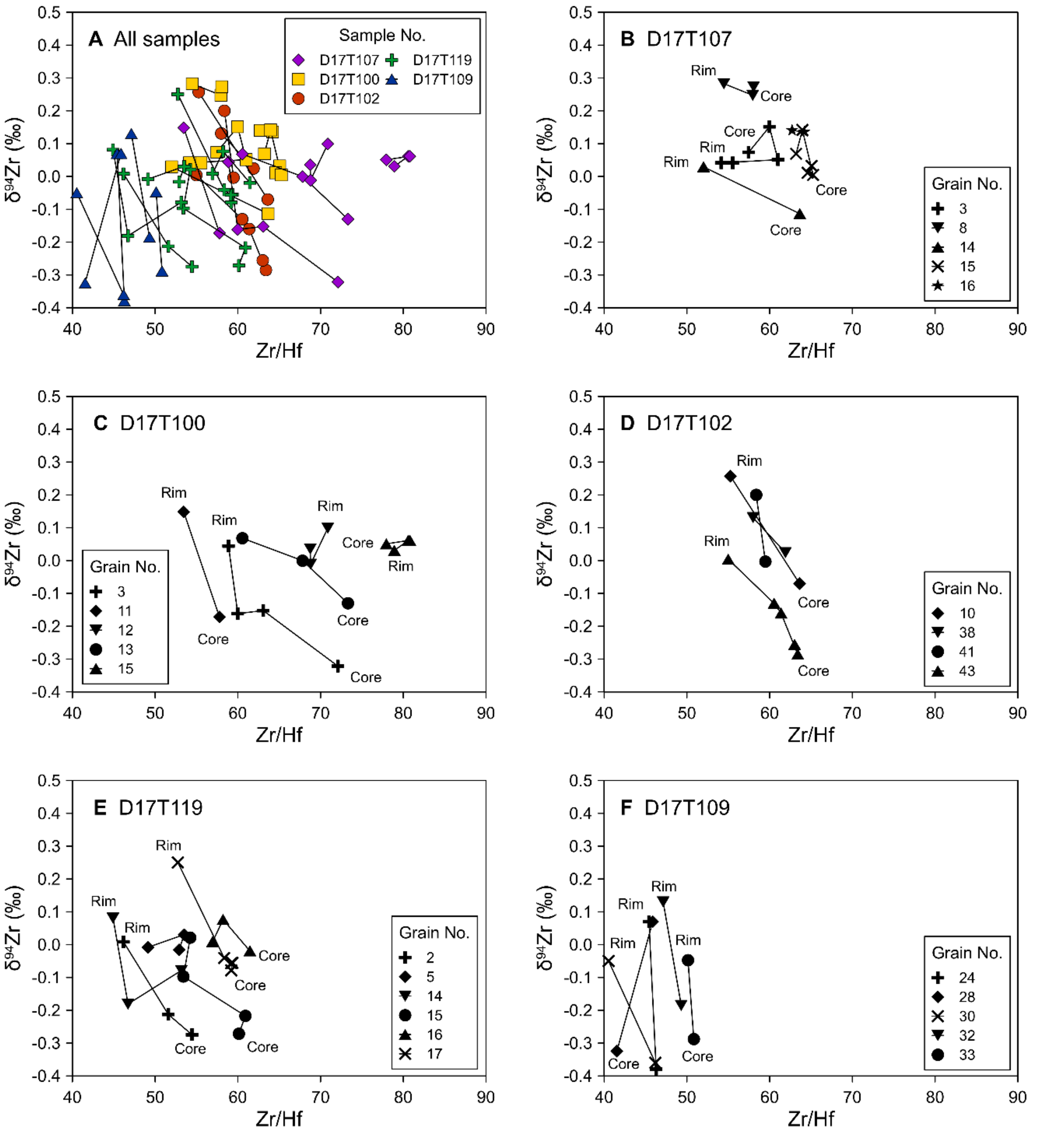

Fig. S5. Representative $\delta^{94/90}Zr_{IPGP\text{-}Zr}$ vs. Zr/Hf in single zircon grains in plutonic arc rocks from southern Tibet. The Zr isotopes and element compositions were measured by fs-LA-MC-ICP-MS using a 20 μm spot size and by LA-ICP-MS using a 24 μm spot size, respectively.

SI References

1. M. J. Farner, C.-T. A. Lee, K. D. Putirka, Mafic–felsic magma mixing limited by reactive processes: A case study of biotite-rich rinds on mafic enclaves. *Earth Planet. Sci. Lett.* **393**, 49–59 (2014).
2. L. Ma, *et al.*, Late Cretaceous crustal growth in the Gangdese area, southern Tibet: Petrological and Sr–Nd–Hf–O isotopic evidence from Zhengga diorite–gabbro. *Chem. Geol.* **349–350**, 54–70 (2013).
3. I. N. Bindeman, O. E. Melnik, Zircon survival, rebirth and recycling during crustal melting, magma crystallization, and mixing based on numerical modelling. *J. Petrol.* (2016).
4. Z. Hu, *et al.*, Signal enhancement in laser ablation ICP-MS by addition of nitrogen in the central channel gas. *J. Anal. At. Spectrom.* **23**, 1093–1101 (2008).
5. Z. Hu, *et al.*, A "wire" signal smoothing device for laser ablation inductively coupled plasma mass spectrometry analysis. *Spectrochim. Acta Part B At. Spectrosc.* **78**, 50–57 (2012).
6. M. Wiedenbeck, *et al.*, Three natural zircon standards for U-Th-Pb, Lu-Hf, trace element and REE analyses. *Geostandards Newslett.: J. Geostandards Geoanalysis* **19**, 1–23 (1995).
7. S. E. Jackson, N. J. Pearson, W. L. Griffin, E. A. Belousova, The application of laser ablation-inductively coupled plasma-mass spectrometry to in situ U–Pb zircon geochronology. *Chem. Geol.* **211**, 47–69 (2004).
8. D. Szymanowski, *et al.*, Isotope-dilution anchoring of zircon reference materials for accurate Ti-in-zircon thermometry. *Chem. Geol.* **481**, 146–154 (2018).
9. S. Piazolo, E. Belousova, A. La Fontaine, C. Corcoran, J. M. Cairney, Trace element homogeneity from micron- to atomic scale: Implication for the suitability of the zircon GJ-1 as a trace element reference material. *Chem. Geol.* **456**, 10–18 (2017).
10. Z. Hu, *et al.*, Contrasting matrix induced elemental fractionation in NIST SRM and rock glasses during laser ablation ICP-MS analysis at high spatial resolution. *J. Anal. At. Spectrom.* **26**, 425–430 (2011).
11. Y. Liu, *et al.*, In situ analysis of major and trace elements of anhydrous minerals by LA-ICP-MS without applying an internal standard. *Chem. Geol.* **257**, 34–43 (2008).
12. T. Andersen, Correction of common lead in U–Pb analyses that do not report ^{204}Pb. *Chem. Geol.* **192**, 59–79 (2002).
13. R. K. Ludwig, User's manual for Isoplot 3.75, a geochronological toolkit for Microsoft Excel. *Berkeley Geochronl. Cent. Spec. Publ.* **No. 5**, 1–75 (2012).
14. E. C. Inglis, *et al.*, Isotopic fractionation of zirconium during magmatic differentiation and the stable isotope composition of the silicate Earth. *Geochim. Cosmochim. Acta* **250**, 311–323 (2019).
15. E. C. Inglis, J. B. Creech, Z. Deng, F. Moynier, High-precision zirconium stable isotope measurements of geological reference materials as measured by double-spike MC-ICPMS. *Chem. Geol.* **493**, 544–552 (2018).
16. W. Zhang, *et al.*, Determination of Zr isotopic ratios in zircons using laser-ablation multiple-collector inductively coupled-plasma mass-spectrometry. *J. Anal. At. Spectrom.* **34**, 1800–1809 (2019).
17. J. B. Chapman, P. Kapp, Tibetan Magmatism Database. *Geochem. Geophys. Geosyst.* **18**, 4229–4234 (2017).

Tables

Table S1. Summary of whole-rock compositions, zircon U–Pb ages, Zr isotope compositions, and Ti-in-zircon temperatures.

Table S2. LA-ICP-MS U–Pb isotope and Ti concentration data of zircons in plutonic arc rocks from southern Tibet.

Table S3. Whole-rock geochemistry of plutonic arc rocks from southern Tibet.

Table S4. In-situ fs-LA-MC-ICP-MS Zr isotope compositions of zircons in plutonic arc rocks from southern Tibet.

Table S5. In-situ LA-ICP-MS major and trace element profile data of zircons in plutonic arc rocks from southern Tibet.

Table S6. Rayleigh distillation modeling results of internal Zr isotope zoning in single zircon grains.

Table S7. In-situ fs-LA-MC-ICP-MS Zr isotope compositions of standard zircons and zircon megacryst Zr-Paki.

Table S8. LA-ICP-MS U–Pb isotope and Ti concentration data of standard zircons.

Table S9. Summary of the operating parameters for MC-ICP-MS and the femtosecond laser ablation system.

Table S1

Summary of whole-rock compositions, zircon U-Pb ages, Zr isotope compositions, and Ti-in-zircon temperatures.

Sample No.	Rock Type *	SiO_2	Zr	Age †	$\delta^{94}Zr$ range (‰)			$\delta^{94}Zr$ average (‰)				α ‡	T § (°C)
		wt.%	ppm	Ma	Min	Max	$\Delta_{max\text{-}min}$	Core	Mantle	Rim	$\Delta_{Rim\text{-}Core}$	Average	Median
D17T107	Hb gabbro	45.35	38.8	94.0±0.7	-0.20	0.39	0.59	0.04	0.08	0.14	0.10	0.99988	831
D17T100	Hb gabbro	42.58	31.3	93.8±0.7	-0.32	0.19	0.51	-0.11	-0.03	0.05	0.16	0.99984	804
D17T102	Hb gabbro	44.64	47.0	94.8±1.5	-0.47	0.41	0.88	-0.22	-0.10	0.16	0.38	0.99974	743
D17T091	Tonalite	65.26	77.2	94.2±1.9	-0.31	0.34	0.65	-0.13	-0.01	0.10	0.22	0.99974	726
D17T119	Tonalite	67.87	89.8	93.0±0.8	-0.27	0.25	0.53	-0.13	-0.08	0.06	0.19	0.99978	742
D17T109	Bi-rich enclave	49.26	62.0	96.2±1.3	-0.86	0.32	1.18	-0.41	-0.32	0.06	0.47	0.99955	689

*Hb = hornblende, Bi = biotite. † Weighted mean $^{206}Pb/^{238}U$ ages of zircon with 2σ errors obtained by LA-ICPMS. ‡ Zircon-melt fractionation factors estimated from the best-fit Rayleigh distillation models. § Median Ti-in-zircon temperatures.

Table S2

LA-ICP-MS U-Pb isotope and Ti concentration data of zircons in plutonic arc rocks from southern Tibet.

Analysis No.	Spot No.	Pb	Th	U	Th/U	f_{206Pbc} *	U-Pb Ratios				
		ppm	ppm	ppm		%	$^{207}Pb/^{235}U$	1σ	$^{206}Pb/^{238}U$	1σ	rho
Hornblende gabbro D17T107											
JUL17A30	1	29.4	2549	1120	2.3	.	0.0942	0.0029	0.01452	0.00014	0.08
JUL17A31	2	4.7	242	220	1.1	1.9	0.0991	0.0091	0.01450	0.00029	0.66
JUL17A32	3	15.5	799	569	1.4	9.2	0.0914	0.0089	0.01438	0.00024	0.55
JUL17A33	4	59.9	4785	2313	2.1	.	0.1055	0.0024	0.01506	0.00013	0.08
JUL17A34	5	12.7	858	547	1.6	.	0.1016	0.0040	0.01482	0.00018	0.08
JUL17A35	6	31.4	3096	1056	2.9	.	0.1006	0.0034	0.01487	0.00016	0.08
JUL17A36	7	7.1	314	362	0.9	.	0.1106	0.0051	0.01476	0.00023	0.08
JUL17A39	8	13.2	895	611	1.5	.	0.1056	0.0047	0.01432	0.00020	0.19
JUL17A40	9	34.8	3030	1239	2.4	.	0.0992	0.0035	0.01522	0.00016	0.09
JUL17A41	10	6.2	367	274	1.3	.	0.1298	0.0055	0.01484	0.00025	0.20
JUL17A42	11	7.2	415	328	1.3	.	0.1189	0.0051	0.01512	0.00023	0.09
JUL17A43	12	38.4	2351	1669	1.4	.	0.0987	0.0028	0.01486	0.00015	0.23
JUL17A44	13	4.3	243	227	1.1	.	0.1542	0.0071	0.01389	0.00028	0.09
JUL17A45	14	4.0	185	157	1.2	10.9	0.1253	0.0212	0.01380	0.00050	0.62
JUL17A46	15C	14.4	1002	626	1.6	.	0.1040	0.0040	0.01449	0.00017	0.01
JUL17A51	16C	15.6	1137	643	1.8	.	0.1049	0.0042	0.01492	0.00019	0.10
JUL17A52	16R	7.6	514	350	1.5	.	0.1193	0.0053	0.01436	0.00023	0.22
JUL17A53	17	12.7	860	550	1.6	.	0.1073	0.0041	0.01465	0.00017	0.20
Hornblende gabbro D17T100											
JUL17A07	1	3.7	133	198	0.7	1.1	0.1193	0.0084	0.01442	0.00028	0.56
JUL17A08	2	6.3	308	301	1.0	.	0.1208	0.0055	0.01527	0.00021	0.08
JUL17A09	3	5.7	264	259	1.0	.	0.1246	0.0066	0.01473	0.00030	0.08
JUL17A10	4	12.9	671	623	1.1	.	0.0977	0.0035	0.01449	0.00017	0.08
JUL17A11	5	9.9	537	473	1.1	.	0.1008	0.0039	0.01450	0.00018	0.08
JUL17A12	6	5.7	329	251	1.3	1.8	0.1013	0.0100	0.01449	0.00031	0.70
JUL17A13	7	11.2	599	530	1.1	.	0.0987	0.0044	0.01481	0.00018	0.08
JUL17A14	8	23.5	1729	964	1.8	.	0.0986	0.0033	0.01480	0.00016	0.08
JUL17A17	9	13.9	726	646	1.1	.	0.0996	0.0040	0.01499	0.00018	0.08
JUL17A18	10	34.2	2825	1311	2.2	.	0.0990	0.0028	0.01466	0.00013	0.08
JUL17A19	11	7.2	349	357	1.0	.	0.1026	0.0045	0.01441	0.00020	0.08
JUL17A20	12	8.2	690	325	2.1	.	0.1150	0.0053	0.01507	0.00026	0.08
JUL17A21	13	6.6	394	317	1.2	.	0.1151	0.0051	0.01432	0.00020	0.08
JUL17A22	14	5.2	198	270	0.7	1.2	0.1012	0.0078	0.01445	0.00030	0.59
JUL17A23	14R	14.4	932	670	1.4	.	0.1037	0.0039	0.01412	0.00017	0.08
JUL17A24	15	10.5	718	447	1.6	.	0.1048	0.0046	0.01457	0.00019	0.08
JUL17A29	16	3.7	234	183	1.3	.	0.1478	0.0060	0.01439	0.00025	0.08
Hornblende gabbro D17T102											
AUG22A33	1	7.1	389	292	1.3	.	0.1037	0.0072	0.01542	0.00022	0.21
AUG22A34	2	4.7	177	221	0.8	.	0.1127	0.0095	0.01518	0.00025	0.19
AUG22A37	3	6.4	398	291	1.4	.	0.0735	0.0079	0.01407	0.00020	0.13
AUG22A38	4	4.3	190	194	1.0	1.3	0.1059	0.0099	0.01530	0.00025	0.47
AUG22A39	5	6.4	381	285	1.3	.	0.1207	0.0088	0.01486	0.00023	0.21
AUG22A40	6	9.6	684	413	1.7	.	0.0938	0.0060	0.01416	0.00017	0.19
AUG22A41	7	4.7	261	218	1.2	.	0.0829	0.0083	0.01421	0.00022	0.16
AUG22A42	8	4.7	280	222	1.3	.	0.0923	0.0117	0.01476	0.00026	0.14
AUG22A43	9	4.2	236	185	1.3	.	0.1225	0.0132	0.01502	0.00027	0.17
AUG22A44	10	5.0	183	241	0.8	.	0.1056	0.0086	0.01488	0.00023	0.19
AUG22A47	11	6.2	366	278	1.3	.	0.1113	0.0081	0.01444	0.00025	0.24
AUG22A48	12	7.1	387	301	1.3	1.3	0.0993	0.0077	0.01475	0.00023	0.57
AUG22A49	13	6.2	283	287	1.0	.	0.1062	0.0075	0.01530	0.00024	0.22
AUG22A50	14	6.7	394	286	1.4	.	0.1030	0.0084	0.01470	0.00022	0.18
AUG22A51	15	5.5	301	234	1.3	.	0.0942	0.0095	0.01503	0.00024	0.16
AUG22A52	16	3.3	134	156	0.9	.	0.1095	0.0120	0.01535	0.00028	0.17
AUG22A53	17	13.9	781	672	1.2	.	0.0965	0.0043	0.01370	0.00016	0.26
AUG22A54	18	10.3	766	423	1.8	.	0.0921	0.0056	0.01408	0.00017	0.20
AUG22A57	19	6.9	398	296	1.3	.	0.0993	0.0077	0.01459	0.00023	0.20

U-Pb Ages (Ma)					Ti	T ‡
$^{207}Pb/^{235}U$	1σ	$^{206}Pb/^{238}U$	1σ	Conc. (%) †	ppm	°C
91.4	2.6	92.9	0.9	102	16.5	869
95.9	8.3	92.8	1.8	97	12.2	836
88.8	8.2	92.0	1.5	104		
101.8	2.2	96.4	0.8	95	7.7	790
98.3	3.7	94.8	1.1	97	11.6	831
97.3	3.1	95.2	1.0	98	13.1	843
106.5	4.7	94.5	1.5	89	6.4	772
101.9	4.3	91.7	1.3	90	10.4	820
96.0	3.2	97.4	1.0	101	15.2	860
123.9	4.9	95.0	1.6	77	8.7	802
114.1	4.6	96.7	1.5	85	9.5	811
95.6	2.6	95.1	1.0	100	8.3	797
145.6	6.2	88.9	1.8	61	11.9	833
119.9	18.9	88.4	3.2	74	10.1	817
100.4	3.6	92.7	1.1	92	8.8	803
101.3	3.8	95.5	1.2	94	17.0	872
114.5	4.8	91.9	1.5	80	11.7	832
103.5	3.8	93.8	1.1	91	13.9	850
					Median	831
					2SE	14
114.5	7.6	92.3	1.8	81	2.2	689
115.8	5.0	97.7	1.3	84	6.5	782
119.2	6.0	94.3	1.9	79	8.9	813
94.7	3.2	92.7	1.1	98		
97.5	3.6	92.8	1.1	95	7.0	790
98.0	9.2	92.7	2.0	95	7.1	791
95.5	4.0	94.8	1.1	99	9.8	823
95.4	3.0	94.7	1.0	99	9.4	819
96.4	3.7	95.9	1.1	99	6.5	783
95.9	2.6	93.8	0.8	98	7.9	802
99.1	4.1	92.2	1.3	93	4.2	742
110.5	4.8	96.4	1.7	87	10.5	830
110.7	4.6	91.7	1.3	83		
97.9	7.2	92.5	1.9	94	5.5	766
100.1	3.6	90.4	1.1	90	8.5	809
101.2	4.2	93.2	1.2	92	10.8	833
139.9	5.3	92.1	1.6	66	4.7	753
					Median	791
					2SE	20
100.2	6.6	98.6	1.4	98	7.9	789
108.5	8.7	97.1	1.6	90	5.8	760
72.0	7.4	90.1	1.3	125	7.9	789
102.2	9.0	97.9	1.6	96	5.7	758
115.7	8.0	95.1	1.5	82		
91.0	5.5	90.6	1.1	100	6.1	764
80.9	7.8	91.0	1.4	112	5.5	754
89.6	10.8	94.5	1.7	105	7.3	781
117.3	11.9	96.1	1.7	82	8.5	796
102.0	7.8	95.2	1.5	93	6.0	763
107.1	7.4	92.4	1.6	86	5.4	752
96.1	7.1	94.4	1.5	98		
102.4	6.9	97.9	1.5	96	6.8	774
99.6	7.7	94.1	1.4	94		
91.4	8.7	96.2	1.5	105	9.0	801
105.5	11.0	98.2	1.8	93	4.1	729
93.6	4.0	87.7	1.0	94		
89.5	5.2	90.1	1.1	101		
96.1	7.1	93.4	1.5	97	4.5	737

Analysis No.	Spot No.	Pb	Th	U	Th/U	f_{206Pbc} *	U-Pb Ratios				
		ppm	ppm	ppm		%	$^{207}Pb/^{235}U$	1σ	$^{206}Pb/^{238}U$	1σ	rho
AUG22A58	20	14.5	710	690	1.0	.	0.1003	0.0043	0.01428	0.00020	0.32
AUG22A59	21	9.7	502	418	1.2	.	0.0927	0.0049	0.01501	0.00019	0.24
AUG22A60	22	4.9	202	230	0.9	.	0.1046	0.0094	0.01501	0.00024	0.18
AUG22A61	23	4.4	228	184	1.2	.	0.1252	0.0160	0.01590	0.00032	0.16
AUG22A62	24	7.2	316	301	1.1	.	0.0978	0.0070	0.01635	0.00026	0.22
AUG22A63	25	11.7	549	538	1.0	.	0.0936	0.0067	0.01552	0.00027	0.24
AUG22A64	26	6.3	290	288	1.0	.	0.0991	0.0077	0.01523	0.00024	0.20
Tonalite D17T091											
AUG22B07	1	5.1	172	262	0.7	1.0	0.1066	0.0103	0.01430	0.00029	0.45
AUG22B08	2	5.9	229	260	0.9	.	0.1032	0.0107	0.01630	0.00025	0.15
AUG22B09	3	9.9	386	479	0.8	.	0.0979	0.0056	0.01511	0.00019	0.22
AUG22B11	5	8.5	368	440	0.8	.	0.1002	0.0054	0.01399	0.00019	0.25
AUG22B12	6	4.7	164	231	0.7	.	0.1066	0.0095	0.01536	0.00025	0.19
AUG22B13	7	6.1	237	307	0.8	.	0.1136	0.0073	0.01468	0.00023	0.24
AUG22B14	8	2.4	83	122	0.7	.	0.1073	0.0139	0.01479	0.00029	0.15
AUG22B17	9	5.4	177	265	0.7	.	0.0998	0.0087	0.01530	0.00024	0.18
AUG22B18	10	5.6	191	268	0.7	.	0.0958	0.0086	0.01554	0.00022	0.16
AUG22B19	11	5.5	222	303	0.7	.	0.0827	0.0080	0.01366	0.00022	0.17
AUG22B20	12	10.2	525	499	1.1	.	0.0900	0.0050	0.01423	0.00017	0.22
AUG22B21	13	5.0	147	243	0.6	.	0.1147	0.0085	0.01544	0.00024	0.21
AUG22B22	14	6.9	270	328	0.8	.	0.1021	0.0069	0.01493	0.00019	0.19
AUG22B23	15	3.7	102	186	0.6	.	0.1068	0.0122	0.01535	0.00030	0.17
AUG22B24	16	7.2	317	364	0.9	.	0.0908	0.0076	0.01396	0.00020	0.17
AUG22B27	17	5.6	190	279	0.7	.	0.1085	0.0082	0.01533	0.00023	0.20
AUG22B28	18	4.2	124	218	0.6	.	0.1242	0.0105	0.01497	0.00026	0.21
AUG22B29	19	7.5	255	381	0.7	.	0.0988	0.0056	0.01482	0.00021	0.25
AUG22B30	20	5.5	199	270	0.7	.	0.1026	0.0077	0.01492	0.00022	0.20
AUG22B31	21R	6.0	178	318	0.6	.	0.1025	0.0068	0.01441	0.00021	0.21
AUG22B32	21C	3.9	106	213	0.5	.	0.1011	0.0099	0.01429	0.00026	0.18
Tonalite D17T119											
JUL18B30	2	4.5	127	244	0.5	.	0.1211	0.0049	0.01486	0.00027	0.06
JUL18B31	3C	2.6	71	145	0.5	2.0	0.1264	0.0103	0.01438	0.00033	0.51
JUL18B32	3R	4.1	112	227	0.5	1.0	0.1040	0.0076	0.01439	0.00028	0.48
JUL18B33	4	3.3	84	179	0.5	1.5	0.1239	0.0089	0.01466	0.00028	0.42
JUL18B34	5	5.5	196	293	0.7	.	0.1048	0.0041	0.01428	0.00021	0.06
JUL18B35	6C	2.6	73	140	0.5	2.2	0.1193	0.0097	0.01453	0.00033	0.47
JUL18B36	6R	5.0	157	266	0.6	.	0.1186	0.0052	0.01438	0.00023	0.06
JUL18B39	7	7.9	354	389	0.9	.	0.1068	0.0045	0.01469	0.00021	0.50
JUL18B40	8	4.3	172	220	0.8	.	0.1250	0.0059	0.01463	0.00025	0.20
JUL18B41	9	4.0	120	208	0.6	1.7	0.1074	0.0092	0.01480	0.00029	0.50
JUL18B42	10	7.9	356	389	0.9	.	0.1094	0.0044	0.01434	0.00019	0.04
JUL18B43	11	5.3	221	265	0.8	.	0.1280	0.0050	0.01453	0.00022	0.04
JUL18B44	12	5.5	172	284	0.6	.	0.1234	0.0051	0.01491	0.00024	0.11
JUL18B45	13	6.7	238	359	0.7	.	0.1154	0.0053	0.01410	0.00020	0.08
JUL18B46	14	2.5	86	136	0.6	3.5	0.1301	0.0128	0.01374	0.00037	0.56
JUL18B49	15	5.3	132	293	0.5	.	0.1205	0.0052	0.01467	0.00026	0.13
JUL18B50	15R	2.7	70	150	0.5	2.8	0.1291	0.0106	0.01399	0.00034	0.49
JUL18B51	16	4.9	185	260	0.7	.	0.1117	0.0050	0.01445	0.00027	0.09
JUL18B52	17	6.1	242	309	0.8	.	0.1179	0.0054	0.01494	0.00023	0.50
Biotite-rich enclave D17T109											
AUG22B33	1	6.1	228	290	0.8	.	0.1155	0.0089	0.01503	0.00020	0.18
AUG22B34	2	6.1	211	286	0.7	.	0.1189	0.0087	0.01527	0.00024	0.21
AUG22B37	3	5.8	192	293	0.7	.	0.0984	0.0077	0.01480	0.00024	0.21
AUG22B38	4	7.3	247	381	0.6	1.8	0.1009	0.0092	0.01358	0.00023	0.48
AUG22B39	5	3.7	112	192	0.6	.	0.1027	0.0160	0.01417	0.00031	0.14
AUG22B41	7	6.8	218	357	0.6	1.2	0.1068	0.0110	0.01390	0.00026	0.51
AUG22B42	8	2.7	64	135	0.5	.	0.1042	0.0169	0.01510	0.00035	0.14
AUG22B43	9	11.3	408	490	0.8	.	0.1402	0.0087	0.01587	0.00028	0.28
AUG22B44	10	4.4	162	238	0.7	.	0.0935	0.0113	0.01393	0.00029	0.17

U-Pb Ages (Ma)					Ti	T ‡
$^{207}Pb/^{235}U$	1σ	$^{206}Pb/^{238}U$	1σ	Conc. (%) †	ppm	°C
97.0	4.0	91.4	1.3	94	7.0	778
90.0	4.5	96.0	1.2	107	7.8	788
101.0	8.6	96.0	1.5	95	5.3	751
119.8	14.3	101.7	2.0	85	6.3	767
94.8	6.5	104.5	1.6	110	6.1	764
90.8	6.2	99.3	1.7	109	3.8	723
95.9	7.1	97.4	1.5	102	8.3	793
					Median	764
					2SE	10
102.9	9.4	91.5	1.8	89	6.7	766
99.8	9.8	104.2	1.6	104	4.4	729
94.8	5.2	*96.7*	1.2	102	3.4	707
97.0	5.0	89.6	1.2	92	4.2	724
102.8	8.7	98.3	1.6	96	3.0	696
109.2	6.6	*93.9*	1.5	86	2.7	687
103.5	12.7	*94.6*	1.8	91		
96.6	8.0	97.9	1.5	101	3.8	715
92.9	8.0	99.4	1.4	107	3.4	705
80.7	7.5	87.5	1.4	108	5.4	746
87.5	4.6	91.1	1.1	104	5.4	745
110.3	7.7	98.8	1.5	90	6.5	762
98.7	6.3	*95.5*	1.2	97	4.9	736
103.0	11.1	98.2	1.9	95	2.7	689
88.3	7.1	89.4	1.3	101	2.5	682
104.6	7.5	98.1	1.5	94	5.0	738
118.9	9.4	95.8	1.7	81	5.2	742
95.6	5.2	*94.8*	1.3	99	4.4	729
99.2	7.1	*95.5*	1.4	96	6.8	766
99.0	6.3	92.2	1.3	93	3.0	697
97.8	9.0	91.5	1.7	94	3.8	714
					Median	726
					2SE	12
116.1	4.4	95.1	1.7	82	5.9	750
120.8	9.3	92.0	2.1	76	5.3	742
100.4	7.0	92.1	1.8	92	3.2	699
118.6	8.0	93.8	1.8	79	6.7	762
101.2	3.7	91.4	1.3	90	6.2	756
114.4	8.8	93.0	2.1	81	4.0	716
113.8	4.7	92.0	1.5	81	5.0	737
103.0	4.1	94.0	1.3	91	5.1	738
119.6	5.3	93.6	1.6	78	4.6	729
103.6	8.4	94.7	1.8	91	3.6	708
105.4	4.1	91.8	1.2	87	2.5	678
122.3	4.5	93.0	1.4	76	4.0	717
118.2	4.6	95.4	1.5	81	2.7	686
110.8	4.8	90.3	1.3	81	3.7	710
124.2	11.5	88.0	2.4	71	3.2	699
115.5	4.7	93.9	1.7	81		
123.3	9.5	89.6	2.2	73	1.9	661
107.5	4.6	92.5	1.7	86	2.1	668
113.2	4.9	95.6	1.5	84	5.7	748
					Median	717
					2SE	14
111.0	8.0	96.2	1.3	87	5.1	716
114.1	7.8	97.7	1.5	86	4.0	697
95.3	7.1	94.7	1.5	99	5.8	728
97.6	8.4	87.0	1.5	89	6.8	741
99.2	14.6	90.7	2.0	91	3.4	683
103.0	10.1	89.0	1.7	86	4.7	709
100.6	15.5	96.6	2.2	96		
133.2	7.7	101.5	1.8	76	2.5	661
90.7	10.4	89.2	1.8	98	5.2	719

Analysis No.	Spot No.	Pb ppm	Th ppm	U ppm	Th/U	f_{206Pbc} * %	U-Pb Ratios $^{207}Pb/^{235}U$	1σ	$^{206}Pb/^{238}U$	1σ	rho
AUG22B47	11	6.1	223	301	0.7	.	0.0962	0.0077	0.01506	0.00023	0.19
AUG22B48	12	5.4	189	286	0.7	.	0.0856	0.0081	0.01441	0.00021	0.15
AUG22B49	13	6.6	214	326	0.7	3.3	0.0966	0.0133	0.01405	0.00034	0.45
AUG22B50	14	7.4	341	331	1.0	.	0.1014	0.0071	0.01570	0.00026	0.24
AUG22B51	15	3.1	91	158	0.6	.	0.1205	0.0135	0.01523	0.00030	0.17
AUG22B52	16	7.2	244	346	0.7	.	0.0977	0.0066	0.01549	0.00021	0.20
AUG22B53	17	6.2	256	308	0.8	.	0.0992	0.0083	0.01476	0.00022	0.18
AUG22B54	18	3.7	129	186	0.7	.	0.1039	0.0114	0.01502	0.00029	0.18
AUG22B57	19	5.9	209	300	0.7	.	0.1152	0.0087	0.01481	0.00027	0.24
AUG22B58	20	7.5	291	361	0.8	.	0.0978	0.0070	0.01542	0.00023	0.21
AUG22B59	21	5.7	150	296	0.5	.	0.1066	0.0075	0.01518	0.00023	0.22
AUG22B60	22	5.1	154	238	0.6	1.2	0.1076	0.0094	0.01576	0.00029	0.53
AUG22B61	23	7.4	292	360	0.8	.	0.0952	0.0065	0.01483	0.00021	0.21
AUG22B62	24	7.0	292	341	0.9	.	0.1136	0.0071	0.01467	0.00020	0.22
AUG22B63	25	7.6	364	379	1.0	.	0.0811	0.0053	0.01401	0.00019	0.21
AUG22B64	26	4.1	120	215	0.6	.	0.0882	0.0086	0.01468	0.00025	0.17

* Concordance = 100 × ($^{206}Pb/^{238}U$ age) / ($^{207}Pb/^{235}U$ age). † Ti-in-zircon temperature.

U-Pb Ages (Ma)					Ti	T ‡
$^{207}Pb/^{235}U$	1σ	$^{206}Pb/^{238}U$	1σ	Conc. (%) †	ppm	°C
93.3	7.1	96.4	1.5	103	4.2	701
83.3	7.5	92.2	1.3	111	4.0	697
93.6	12.2	89.9	2.2	96		
98.1	6.5	100.4	1.6	102	5.5	722
115.5	12.1	97.4	1.9	84	4.1	699
94.7	6.1	99.1	1.3	105	4.3	703
96.0	7.6	94.5	1.4	98	8.0	756
100.4	10.4	96.1	1.8	96	3.5	686
110.7	7.9	94.8	1.7	86	5.2	718
94.7	6.5	98.6	1.5	104	5.2	718
102.8	6.9	97.1	1.5	94	6.3	734
103.7	8.6	100.8	1.8	97	4.8	712
92.3	6.0	94.9	1.3	103	3.1	676
109.2	6.5	93.9	1.3	86	3.8	692
79.2	5.0	89.7	1.2	113	6.8	741
85.9	8.0	93.9	1.6	109	6.6	739
					Median	710
					2SE	10

Table S3

Whole-rock geochemistry of plutonic arc rocks from southern Tibet.

Rock Type	Hornblende gabbro			Tonalite		Biotite-rich enclave
Sample No.	D17T107	D17T100	D17T102	D17T091	D17T119	D17T109
Major element compositions (wt.%)						
SiO_2	42.58	45.35	44.64	65.26	67.87	49.26
TiO_2	1.59	1.40	1.80	0.44	0.37	1.01
Al2O3	20.70	19.02	11.74	15.91	15.96	16.55
TFe_2O_3	11.29	12.06	13.72	3.79	3.20	8.61
MnO	0.11	0.16	0.18	0.09	0.07	0.26
MgO	6.63	5.61	10.87	1.46	1.20	4.77
CaO	11.73	10.78	11.92	3.67	3.07	6.06
Na_2O	2.30	3.25	2.05	4.35	4.72	4.12
K_2O	0.65	0.50	0.69	2.31	1.98	4.15
P_2O_5	0.10	0.59	0.16	0.18	0.16	0.32
LOI	1.85	0.81	1.59	2.23	1.19	4.31
Sum	99.51	99.53	99.37	99.68	99.80	99.42
Mg# *	54	48	61	44	43	53
Tace element concentrations (ppm)						
Sc	35.3	23.2	58.7	5.17	4.25	19.4
V	471	359	507	71.7	61.3	187
Cr	16.1	13.3	107	6.45	4.89	24.2
Co	42.9	37.9	57.3	8.75	7.23	23.1
Ni	65.6	23.2	120	7.30	5.77	23.4
Cu	261	137	32.0	76.8	12.7	27.4
Zn	69.1	100	94.6	49.3	41.8	124
Rb	7.97	3.81	6.92	58.9	45.2	148
Sr	1126	1578	572	751	852	639
Y	13.9	20.8	17.1	7.46	7.57	19.6
Zr	31.3	38.8	47.0	77.2	89.8	62.0
Nb	1.79	3.34	2.27	4.20	4.01	9.96
Ba	403	130	118	460	506	476
La	5.63	14.6	7.33	27.0	17.6	17.0
Ce	15.0	39.7	20.5	48.7	34.4	45.3
Pr	2.43	6.08	3.35	4.91	3.70	6.11
Nd	12.9	28.9	16.6	18.0	14.3	27.0
Sm	3.67	6.62	4.69	2.87	2.51	5.85
Eu	1.22	1.86	1.27	0.81	0.77	1.46
Gd	3.47	5.40	4.29	2.03	1.78	4.58
Tb	0.47	0.74	0.61	0.27	0.23	0.64
Dy	2.75	4.00	3.52	1.42	1.37	3.53
Ho	0.50	0.75	0.63	0.27	0.24	0.65
Er	1.31	2.00	1.66	0.74	0.71	1.81
Tm	0.19	0.26	0.22	0.10	0.10	0.27
Yb	1.14	1.77	1.42	0.69	0.71	1.83
Lu	0.15	0.25	0.19	0.12	0.10	0.27
Hf	1.08	1.47	1.63	2.29	2.43	2.10
Ta	0.11	0.14	0.12	0.29	0.28	0.36
Pb	2.19	2.32	1.82	6.64	9.64	6.14
Th	0.62	0.91	0.99	7.53	6.06	4.21
U	0.18	0.25	0.27	1.22	1.48	2.60
Eu/Eu* †	1.05	0.95	0.87	1.02	1.11	0.86

* Mg# =100 × atomic Mg/(Mg + Fe_{total}). † Eu/Eu* = Eu_N / (Sm_N × Gd_N)$^{0.5}$.

Table S4

In-situ LA-MC-ICP-MS Zr isotope compositions of zircons in plutonic arc rocks from southern Tibet.

Analysis No.	Spot No. *	Domain †	$^{94}Zr/^{90}Zr$	2SE	$^{94}Zr/^{91}Zr$	2SE	$^{96}Zr/^{90}Zr$	2SE	^{90}Zr (V)
Hornblende gabbro D17T107									
JUL19B057	3-1	r	0.359767	0.000011	1.624801	0.000066	0.059630	0.000004	11.4
JUL19B058	3-2	m	0.359771	0.000011	1.624779	0.000067	0.059630	0.000004	11.4
JUL19B060	3-3	c	0.359801	0.000010	1.624849	0.000055	0.059638	0.000003	12.0
JUL19B061	3-4	c	0.359773	0.000010	1.624738	0.000054	0.059630	0.000004	12.1
JUL19B063	3-5	m	0.359765	0.000013	1.624760	0.000067	0.059629	0.000004	11.5
JUL19B064	3-6	m	0.359765	0.000010	1.624734	0.000060	0.059633	0.000004	11.5
JUL19B069	3-7	r	0.359754	0.000014	1.624745	0.000067	0.059629	0.000005	11.3
JUL19B070	6-1	r	0.359836	0.000012	1.625016	0.000057	0.059651	0.000004	10.9
JUL19B072	6-2	c	0.359823	0.000009	1.624953	0.000055	0.059647	0.000003	10.8
JUL19B073	6-3	c	0.359829	0.000009	1.624969	0.000054	0.059644	0.000003	10.7
JUL19B075	6-4	r	0.359872	0.000011	1.625178	0.000058	0.059657	0.000004	10.5
JUL19B076	8-1	r	0.359834	0.000011	1.625034	0.000074	0.059649	0.000004	10.2
JUL19B078	8-2	m	0.359811	0.000011	1.624890	0.000058	0.059642	0.000003	10.0
JUL19B079	8-3	c	0.359820	0.000010	1.624911	0.000064	0.059644	0.000004	9.6
JUL19B081	8-4	c	0.359809	0.000008	1.624942	0.000054	0.059644	0.000003	10.2
JUL19B082	8-5	m	0.359836	0.000010	1.624977	0.000058	0.059649	0.000003	10.4
JUL19B084	8-6	r	0.359836	0.000012	1.624989	0.000061	0.059644	0.000004	10.5
JUL19B085	9-1	r	0.359807	0.000009	1.624841	0.000054	0.059637	0.000004	11.7
JUL19C005	9-2	m	0.359753	0.000010	1.624759	0.000059	0.059625	0.000003	12.0
JUL19C006	9-3	c	0.359744	0.000010	1.624652	0.000055	0.059626	0.000003	12.8
JUL19C008	9-4	c	0.359730	0.000008	1.624591	0.000052	0.059622	0.000003	12.9
JUL19C009	12-1	m	0.359648	0.000008	1.624349	0.000054	0.059601	0.000003	14.4
JUL19C011	12-2	m	0.359713	0.000012	1.624601	0.000054	0.059617	0.000003	13.8
JUL19C012	12-3	c	0.359647	0.000010	1.624373	0.000051	0.059601	0.000003	14.1
JUL19C014	12-4	m	0.359690	0.000011	1.624523	0.000058	0.059613	0.000003	14.3
JUL19C015	12-5	r	0.359758	0.000010	1.624760	0.000050	0.059628	0.000003	13.8
JUL19C017	13-1	r	0.359813	0.000008	1.624895	0.000047	0.059646	0.000003	14.8
JUL19C018	13-2	m	0.359787	0.000008	1.624812	0.000057	0.059636	0.000003	14.2
JUL19C020	13-3	m	0.359785	0.000009	1.624793	0.000051	0.059630	0.000003	14.2
JUL19C021	13-4	m	0.359778	0.000011	1.624785	0.000050	0.059632	0.000003	14.2
JUL19C030	13-5	c	0.359776	0.000010	1.624767	0.000065	0.059632	0.000003	14.4
JUL19C031	13-6	m	0.359783	0.000008	1.624823	0.000052	0.059635	0.000003	14.5
JUL19C033	14-1	m	0.359683	0.000009	1.624443	0.000047	0.059608	0.000003	15.3
JUL19C034	14-2	c	0.359664	0.000011	1.624384	0.000048	0.059600	0.000003	14.8
JUL19C036	14-3	c	0.359663	0.000009	1.624396	0.000049	0.059604	0.000003	15.2
JUL19C037	14-4	c	0.359642	0.000009	1.624299	0.000053	0.059599	0.000004	14.8
JUL19C039	14-5	m	0.359692	0.000009	1.624482	0.000048	0.059611	0.000003	14.7
JUL19C040	14-6	r	0.359729	0.000010	1.624609	0.000050	0.059621	0.000003	14.9
JUL19C042	15-1	c	0.359713	0.000011	1.624540	0.000063	0.059619	0.000003	14.7
JUL19C043	15-2	c	0.359740	0.000009	1.624625	0.000052	0.059622	0.000003	14.7
JUL19C045	15-3	c	0.359722	0.000010	1.624587	0.000063	0.059614	0.000003	14.4
JUL19C046	15-4	c	0.359719	0.000008	1.624571	0.000047	0.059619	0.000003	14.5
JUL19C051	15-5	m	0.359753	0.000010	1.624716	0.000054	0.059625	0.000002	14.8
JUL19C052	15-6	r	0.359727	0.000010	1.624637	0.000049	0.059620	0.000003	14.5
JUL19C054	16-1	r	0.359777	0.000010	1.624794	0.000048	0.059634	0.000003	14.4
JUL19C055	16-2	m	0.359749	0.000012	1.624658	0.000052	0.059621	0.000003	14.2
JUL19C057	16-3	c	0.359769	0.000009	1.624748	0.000057	0.059625	0.000003	14.3
JUL19C058	16-4	c	0.359773	0.000009	1.624763	0.000046	0.059632	0.000003	14.4
JUL19C060	16-5	m	0.359767	0.000008	1.624757	0.000052	0.059627	0.000003	14.5
JUL19C061	16-6	r	0.359762	0.000009	1.624706	0.000049	0.059627	0.000003	14.2
JUL19C063	18-1	r	0.359722	0.000008	1.624583	0.000051	0.059614	0.000003	14.6
JUL19C064	18-2	m	0.359715	0.000009	1.624595	0.000050	0.059615	0.000003	14.5
JUL19C066	18-3	c	0.359689	0.000010	1.624461	0.000048	0.059608	0.000003	14.3
JUL19C067	18-4	c	0.359708	0.000009	1.624567	0.000056	0.059615	0.000003	14.6
JUL19C072	18-5	r	0.359697	0.000010	1.624490	0.000051	0.059615	0.000003	14.5
JUL19C073	19-1	r	0.359743	0.000010	1.624689	0.000059	0.059619	0.000003	14.3
JUL19C075	19-2	m	0.359727	0.000009	1.624611	0.000049	0.059619	0.000003	14.1
JUL19C076	19-3	c	0.359717	0.000012	1.624584	0.000055	0.059616	0.000004	14.0
JUL19C078	19-4	m	0.359727	0.000009	1.624606	0.000050	0.059620	0.000003	14.2
JUL19C079	19-5	r	0.359712	0.000011	1.624544	0.000057	0.059614	0.000003	14.1
Hornblende gabbro D17T100									
JUL19A079	3-1	r	0.359756	0.000012	1.624672	0.000059	0.059621	0.000004	14.6

$\delta^{94/90}Zr_{GJ-1}$	2SE	$\delta^{94/91}Zr_{GJ-1}$	2SE	$\delta^{96/90}Zr_{GJ-1}$	2SE	$\delta^{94}Zr = \delta^{94/90}Zr_{IPGP-Zr}$ ‡	2SE
0.05	0.09	0.03	0.09	0.06	0.13	0.04	0.10
0.06	0.09	0.02	0.09	0.06	0.13	0.05	0.10
0.16	0.09	0.09	0.08	0.22	0.13	0.15	0.10
0.09	0.09	0.02	0.08	0.10	0.13	0.07	0.10
0.07	0.09	0.02	0.08	0.01	0.13	0.06	0.10
0.07	0.09	0.00	0.08	0.07	0.13	0.06	0.10
0.02	0.09	0.00	0.08	0.01	0.15	0.00	0.10
0.24	0.09	0.17	0.08	0.37	0.14	0.23	0.10
0.20	0.09	0.14	0.08	0.31	0.13	0.19	0.10
0.22	0.09	0.15	0.08	0.25	0.13	0.21	0.10
0.40	0.09	0.31	0.08	0.54	0.13	0.39	0.10
0.29	0.09	0.22	0.08	0.39	0.14	0.28	0.10
0.26	0.09	0.13	0.08	0.29	0.14	0.25	0.10
0.29	0.09	0.14	0.08	0.32	0.14	0.27	0.10
0.20	0.09	0.13	0.08	0.26	0.14	0.19	0.10
0.27	0.09	0.15	0.08	0.35	0.14	0.26	0.10
0.29	0.09	0.18	0.08	0.30	0.13	0.28	0.10
0.21	0.09	0.09	0.07	0.18	0.13	0.20	0.10
0.10	0.09	0.08	0.08	0.05	0.14	0.09	0.10
0.08	0.09	0.02	0.08	0.06	0.13	0.07	0.10
0.04	0.09	-0.02	0.08	0.02	0.13	0.03	0.10
-0.19	0.09	-0.17	0.08	-0.32	0.14	-0.20	0.10
0.00	0.10	-0.01	0.08	-0.06	0.13	-0.01	0.11
-0.19	0.10	-0.15	0.08	-0.33	0.13	-0.20	0.11
-0.04	0.10	-0.04	0.08	-0.10	0.13	-0.06	0.11
0.14	0.10	0.11	0.07	0.15	0.13	0.13	0.10
0.29	0.09	0.19	0.07	0.46	0.13	0.28	0.10
0.21	0.09	0.13	0.08	0.30	0.13	0.20	0.10
0.22	0.09	0.11	0.08	0.20	0.12	0.21	0.10
0.20	0.09	0.10	0.08	0.24	0.12	0.19	0.10
0.16	0.09	0.07	0.09	0.23	0.14	0.15	0.10
0.18	0.09	0.11	0.08	0.28	0.14	0.17	0.10
-0.10	0.08	-0.13	0.07	-0.16	0.12	-0.11	0.09
-0.15	0.09	-0.16	0.07	-0.31	0.12	-0.17	0.10
-0.10	0.09	-0.12	0.07	-0.19	0.13	-0.11	0.10
-0.16	0.09	-0.18	0.07	-0.27	0.14	-0.17	0.10
0.04	0.09	-0.04	0.07	-0.01	0.12	0.03	0.10
0.14	0.09	0.04	0.07	0.16	0.12	0.13	0.10
0.04	0.09	-0.03	0.08	0.04	0.13	0.03	0.10
0.12	0.09	0.03	0.07	0.09	0.12	0.11	0.10
0.02	0.09	-0.02	0.08	-0.10	0.13	0.01	0.10
0.02	0.09	-0.03	0.08	-0.02	0.13	0.00	0.10
0.15	0.09	0.10	0.07	0.15	0.13	0.14	0.10
0.08	0.09	0.05	0.07	0.06	0.13	0.07	0.10
0.23	0.09	0.14	0.08	0.34	0.13	0.22	0.10
0.15	0.09	0.06	0.08	0.12	0.14	0.14	0.10
0.15	0.09	0.07	0.08	0.11	0.12	0.14	0.10
0.16	0.09	0.08	0.07	0.24	0.13	0.15	0.10
0.13	0.09	0.07	0.07	0.13	0.12	0.12	0.10
0.12	0.09	0.04	0.07	0.12	0.13	0.11	0.10
0.00	0.09	-0.03	0.07	-0.10	0.12	-0.02	0.10
-0.02	0.09	-0.02	0.07	-0.09	0.12	-0.03	0.10
-0.13	0.09	-0.13	0.07	-0.22	0.13	-0.15	0.10
-0.08	0.09	-0.07	0.07	-0.10	0.13	-0.09	0.10
-0.05	0.09	-0.08	0.07	-0.06	0.12	-0.07	0.10
0.07	0.09	0.04	0.08	0.02	0.13	0.06	0.10
0.05	0.09	-0.01	0.08	0.03	0.13	0.04	0.10
0.02	0.09	-0.03	0.08	-0.02	0.13	0.01	0.10
0.05	0.08	-0.01	0.08	0.02	0.12	0.04	0.09
0.01	0.09	-0.05	0.08	-0.07	0.12	0.00	0.10
						0.08±0.03 (2SE)	
0.06	0.08	-0.03	0.07	-0.10	0.11	0.04	0.09

Analysis No.	Spot No. *	Domain †	$^{94}Zr/^{90}Zr$	2SE	$^{94}Zr/^{91}Zr$	2SE	$^{96}Zr/^{90}Zr$	2SE	^{90}Zr (V)
JUL19A081	3-2	m	0.359713	0.000009	1.624557	0.000050	0.059615	0.000003	14.9
JUL19A082	3-3	m	0.359688	0.000010	1.624450	0.000054	0.059606	0.000004	14.6
JUL19A084	3-4	c	0.359692	0.000011	1.624477	0.000056	0.059607	0.000004	14.3
JUL19A085	3-5	c	0.359631	0.000012	1.624297	0.000057	0.059598	0.000004	14.5
JUL19A087	3-6	c	0.359651	0.000010	1.624307	0.000053	0.059596	0.000003	14.5
JUL19A088	3-7	m	0.359714	0.000009	1.624552	0.000055	0.059616	0.000003	14.8
JUL19B005	11-1	r	0.359694	0.000010	1.624534	0.000055	0.059606	0.000003	14.5
JUL19B006	11-2	m	0.359693	0.000008	1.624502	0.000049	0.059613	0.000003	14.5
JUL19B008	11-3	c	0.359658	0.000011	1.624393	0.000062	0.059601	0.000003	14.2
JUL19B009	11-4	c	0.359687	0.000010	1.624476	0.000047	0.059606	0.000003	14.4
JUL19B011	11-5	m	0.359672	0.000012	1.624445	0.000053	0.059609	0.000003	14.0
JUL19B012	11-6	r	0.359787	0.000014	1.624798	0.000063	0.059631	0.000004	14.5
JUL19B014	12-1	r	0.359801	0.000012	1.624878	0.000063	0.059638	0.000003	15.0
JUL19B015	12-2	m	0.359770	0.000009	1.624793	0.000048	0.059627	0.000003	14.5
JUL19B017	12-3	c	0.359763	0.000011	1.624732	0.000052	0.059630	0.000004	14.2
JUL19B018	12-4	c	0.359743	0.000012	1.624695	0.000056	0.059623	0.000004	14.2
JUL19B020	12-5	m	0.359731	0.000008	1.624640	0.000044	0.059620	0.000003	14.0
JUL19B021	12-6	r	0.359748	0.000011	1.624702	0.000065	0.059623	0.000004	14.8
JUL19B026	13-1	r	0.359759	0.000010	1.624785	0.000057	0.059628	0.000003	15.1
JUL19B027	13-2	m	0.359735	0.000010	1.624588	0.000056	0.059625	0.000004	14.4
JUL19B029	13-3	c	0.359678	0.000010	1.624482	0.000054	0.059608	0.000003	14.6
JUL19B030	13-4	c	0.359651	0.000009	1.624380	0.000059	0.059602	0.000004	14.6
JUL19B032	13-5	m	0.359765	0.000009	1.624797	0.000052	0.059631	0.000003	12.8
JUL19B033	13-6	r	0.359765	0.000009	1.624797	0.000052	0.059631	0.000003	12.8
JUL19B035	15-1	r	0.359730	0.000012	1.624623	0.000050	0.059619	0.000004	14.5
JUL19B036	15-2	m	0.359712	0.000009	1.624598	0.000053	0.059616	0.000004	12.7
JUL19B038	15-3	m	0.359773	0.000009	1.624801	0.000048	0.059629	0.000003	13.9
JUL19B039	15-4	c	0.359776	0.000010	1.624758	0.000048	0.059631	0.000003	13.9
JUL19B041	15-5	c	0.359757	0.000008	1.624729	0.000056	0.059625	0.000003	14.3
JUL19B042	15-6	m	0.359768	0.000011	1.624766	0.000053	0.059626	0.000003	14.0
JUL19B048	15-7	r	0.359759	0.000011	1.624713	0.000055	0.059625	0.000003	14.4
Hornblende gabbro D17T102									
JUN06B26	1-1	r	0.358683	0.000013	1.620954	0.000068	0.059347	0.000004	10.5
JUN06B27	1-2	c	0.358605	0.000015	1.620720	0.000073	0.059332	0.000003	10.0
JUN06B29	1-3	c	0.358575	0.000013	1.620692	0.000074	0.059325	0.000003	10.6
JUN06B30	1-4	c	0.358513	0.000016	1.620413	0.000082	0.059307	0.000005	10.3
JUN06B31	1-5	c	0.358507	0.000013	1.620412	0.000055	0.059312	0.000003	10.7
JUN06B35	1-6	r	0.358644	0.000012	1.620861	0.000060	0.059343	0.000005	11.0
JUN06B15	10-1	m	0.358623	0.000017	1.620804	0.000075	0.059338	0.000005	7.5
JUN06B16	10-2	m	0.358642	0.000020	1.620936	0.000092	0.059339	0.000005	7.4
JUN06B17	10-3	r	0.358750	0.000013	1.621258	0.000066	0.059369	0.000004	8.5
JUN06B21	38-1	c	0.358607	0.000015	1.620799	0.000070	0.059335	0.000005	7.5
JUN06B22	38-2	c	0.358633	0.000015	1.620861	0.000082	0.059337	0.000005	7.3
JUN06B23	38-3	m	0.358671	0.000013	1.620974	0.000069	0.059350	0.000004	7.5
JUN06B25	38-4	r	0.358711	0.000012	1.621085	0.000062	0.059361	0.000004	8.1
JUN06B07	41-1	r	0.358817	0.000014	1.621429	0.000072	0.059383	0.000005	7.4
JUN06B08	41-2	m	0.358668	0.000019	1.621018	0.000096	0.059354	0.000005	6.7
JUN06B09	41-3	c	0.358643	0.000021	1.620898	0.000099	0.059341	0.000006	7.3
JUN06B11	41-4	c	0.358547	0.000016	1.620567	0.000072	0.059323	0.000004	7.8
JUN06B12	41-5	m	0.358578	0.000016	1.620677	0.000074	0.059327	0.000004	7.9
JUN06B13	41-6	m	0.358652	0.000013	1.620911	0.000064	0.059345	0.000005	8.6
JUN06A31	24-1	m	0.358501	0.000014	1.620327	0.000071	0.059304	0.000005	7.9
JUN06A32	24-2	m	0.358477	0.000016	1.620386	0.000064	0.059299	0.000004	7.5
JUN06A34	24-3	c	0.358496	0.000015	1.620356	0.000063	0.059306	0.000004	7.7
JUN06A35	24-4	c	0.358510	0.000014	1.620384	0.000071	0.059310	0.000005	7.9
JUN06A30	24-5	m	0.358499	0.000011	1.620431	0.000060	0.059308	0.000004	7.9
JUN06A36	24-6	r	0.358622	0.000013	1.620831	0.000071	0.059335	0.000004	8.4
JUN06A11	43-1	r	0.358534	0.000014	1.620516	0.000077	0.059316	0.000004	9.8
JUN06A12	43-2	m	0.358485	0.000016	1.620339	0.000067	0.059300	0.000004	9.4
JUN06A13	43-3	m	0.358483	0.000018	1.620334	0.000087	0.059301	0.000005	8.7
JUN06A16	43-4	c	0.358481	0.000014	1.620329	0.000077	0.059299	0.000005	8.8
JUN06A17	43-5	c	0.358440	0.000013	1.620199	0.000056	0.059296	0.000005	9.9
JUN06A18	43-6	c	0.358451	0.000012	1.620223	0.000070	0.059301	0.000003	10.1
JUN06A20	43-7	r	0.358632	0.000013	1.620843	0.000069	0.059337	0.000004	9.3
JUN06A21	44-1	r	0.358688	0.000012	1.621001	0.000058	0.059350	0.000005	10.0

$\delta^{94/90}Zr_{GJ-1}$	2SE	$\delta^{94/91}Zr_{GJ-1}$	2SE	$\delta^{96/90}Zr_{GJ-1}$	2SE	$\delta^{94}Zr = \delta^{94/90}Zr_{IPGP-Zr}$ ‡	2SE
-0.08	0.08	-0.11	0.07	-0.27	0.12	-0.09	0.09
-0.15	0.08	-0.17	0.07	-0.41	0.12	-0.16	0.09
-0.14	0.09	-0.15	0.07	-0.35	0.12	-0.15	0.10
-0.31	0.09	-0.26	0.07	-0.49	0.12	-0.32	0.10
-0.23	0.09	-0.26	0.08	-0.48	0.12	-0.24	0.10
-0.06	0.09	-0.11	0.08	-0.16	0.12	-0.07	0.10
-0.14	0.09	-0.12	0.08	-0.39	0.12	-0.15	0.10
-0.15	0.09	-0.14	0.08	-0.27	0.12	-0.16	0.10
-0.24	0.10	-0.20	0.08	-0.45	0.12	-0.25	0.11
-0.15	0.10	-0.14	0.08	-0.37	0.12	-0.17	0.11
-0.16	0.09	-0.15	0.07	-0.25	0.12	-0.17	0.10
0.16	0.09	0.06	0.08	0.13	0.13	0.15	0.10
0.20	0.10	0.11	0.09	0.27	0.15	0.19	0.11
0.11	0.10	0.06	0.08	0.09	0.14	0.10	0.11
0.08	0.10	0.02	0.09	0.14	0.15	0.07	0.11
0.03	0.10	0.00	0.09	0.03	0.15	0.01	0.11
0.00	0.08	-0.04	0.07	-0.07	0.12	-0.01	0.09
0.05	0.09	-0.01	0.08	-0.02	0.13	0.04	0.10
0.08	0.09	0.07	0.08	0.06	0.13	0.07	0.10
0.01	0.09	-0.05	0.08	0.00	0.14	0.00	0.10
-0.12	0.10	-0.12	0.08	-0.26	0.13	-0.13	0.11
-0.19	0.10	-0.18	0.08	-0.36	0.14	-0.21	0.11
0.11	0.10	0.08	0.08	0.13	0.14	0.10	0.11
0.11	0.10	0.08	0.08	0.13	0.14	0.10	0.11
-0.02	0.09	-0.07	0.08	-0.08	0.13	-0.04	0.10
-0.07	0.09	-0.08	0.08	-0.12	0.13	-0.09	0.10
0.10	0.09	0.03	0.08	0.11	0.14	0.09	0.10
0.11	0.09	0.00	0.08	0.15	0.14	0.09	0.10
0.04	0.09	-0.01	0.08	0.00	0.14	0.03	0.10
0.07	0.09	0.01	0.08	0.00	0.14	0.06	0.10
0.06	0.09	0.00	0.07	0.02	0.12	0.05	0.10
						-0.04±0.05 (2SE)	
0.11	0.11	-0.02	0.09	0.06	0.14	0.10	0.11
-0.11	0.11	-0.16	0.09	-0.19	0.14	-0.12	0.12
-0.18	0.11	-0.14	0.10	-0.33	0.15	-0.20	0.12
-0.36	0.11	-0.31	0.10	-0.62	0.16	-0.37	0.12
-0.37	0.11	-0.31	0.09	-0.54	0.15	-0.38	0.12
-0.03	0.10	-0.05	0.10	-0.02	0.15	-0.04	0.11
-0.09	0.11	-0.09	0.10	-0.10	0.16	-0.10	0.11
-0.03	0.11	-0.01	0.10	-0.08	0.16	-0.04	0.12
0.27	0.10	0.19	0.10	0.43	0.16	0.26	0.11
-0.03	0.11	-0.06	0.10	-0.02	0.16	-0.05	0.12
0.04	0.11	-0.02	0.10	0.02	0.16	0.02	0.12
0.14	0.11	0.05	0.10	0.24	0.15	0.13	0.12
0.19	0.10	0.06	0.09	0.30	0.14	0.18	0.11
0.42	0.12	0.25	0.11	0.55	0.15	0.41	0.12
0.01	0.12	0.00	0.11	0.08	0.15	0.00	0.13
-0.06	0.12	-0.08	0.11	-0.15	0.16	-0.07	0.13
-0.29	0.12	-0.23	0.11	-0.33	0.15	-0.30	0.12
-0.20	0.12	-0.16	0.11	-0.26	0.15	-0.21	0.12
0.01	0.11	-0.01	0.11	0.04	0.16	-0.01	0.12
-0.20	0.11	-0.21	0.10	-0.43	0.17	-0.21	0.12
-0.27	0.12	-0.17	0.10	-0.51	0.17	-0.28	0.12
-0.21	0.13	-0.19	0.11	-0.37	0.20	-0.22	0.14
-0.17	0.13	-0.17	0.12	-0.30	0.21	-0.18	0.14
-0.21	0.11	-0.14	0.10	-0.36	0.17	-0.22	0.12
0.14	0.13	0.10	0.12	0.12	0.20	0.13	0.13
0.02	0.11	0.00	0.10	-0.05	0.17	0.00	0.11
-0.12	0.11	-0.11	0.09	-0.31	0.17	-0.13	0.12
-0.13	0.11	-0.11	0.10	-0.30	0.18	-0.14	0.12
-0.16	0.11	-0.13	0.10	-0.38	0.17	-0.17	0.12
-0.27	0.11	-0.21	0.09	-0.42	0.17	-0.29	0.12
-0.24	0.11	-0.20	0.10	-0.34	0.16	-0.26	0.12
0.11	0.10	0.08	0.09	0.08	0.14	0.10	0.11
0.27	0.10	0.18	0.09	0.30	0.15	0.26	0.10

Analysis No.	Spot No. *	Domain †	$^{94}Zr/^{90}Zr$	2SE	$^{94}Zr/^{91}Zr$	2SE	$^{96}Zr/^{90}Zr$	2SE	^{90}Zr (V)
JUN06A22	44-2	m	0.358599	0.000012	1.620694	0.000060	0.059333	0.000004	9.6
JUN06A26	44-3	c	0.358469	0.000015	1.620283	0.000064	0.059298	0.000003	10.3
JUN06A27	44-4	c	0.358420	0.000013	1.620112	0.000060	0.059288	0.000003	10.4
JUN06A28	44-5	m	0.358556	0.000012	1.620634	0.000059	0.059323	0.000003	11.0
Tonalite D17T091									
JUN06B50	1-1	c	0.358683	0.000009	1.620975	0.000056	0.059354	0.000003	9.9
JUN06B53	2-1	c	0.358685	0.000013	1.620986	0.000064	0.059358	0.000003	9.1
JUN06B54	3-1	c	0.358677	0.000011	1.620929	0.000060	0.059352	0.000004	8.5
JUN06B55	4-1	m	0.358743	0.000015	1.621245	0.000070	0.059370	0.000004	8.9
JUN06B57	5-1	m	0.358691	0.000013	1.621067	0.000065	0.059361	0.000004	9.1
JUN06B58	6-1	c	0.358668	0.000012	1.620963	0.000066	0.059353	0.000004	9.0
JUN06B59	7-1	r	0.358763	0.000012	1.621268	0.000064	0.059372	0.000005	8.7
JUN06B63	8-1	m	0.358747	0.000011	1.621207	0.000054	0.059365	0.000003	8.4
JUN06B64	9-1	r	0.358741	0.000011	1.621209	0.000063	0.059364	0.000004	8.6
JUN06B51	22-1	m	0.358716	0.000012	1.621063	0.000062	0.059364	0.000004	9.5
JUN06B36	23-1	r	0.358713	0.000015	1.621058	0.000067	0.059359	0.000005	10.4
JUN06B37	23-2	m	0.358606	0.000014	1.620731	0.000071	0.059333	0.000004	9.5
JUN06B39	23-3	m	0.358646	0.000016	1.620866	0.000082	0.059348	0.000004	9.1
JUN06B40	23-4	m	0.358668	0.000015	1.620932	0.000062	0.059346	0.000004	9.0
JUN06B41	23-5	c	0.358621	0.000015	1.620783	0.000076	0.059341	0.000005	9.2
JUN06B43	23-6	c	0.358569	0.000016	1.620634	0.000072	0.059323	0.000004	9.3
JUN06B44	23-7	c	0.358595	0.000015	1.620687	0.000076	0.059330	0.000004	9.2
JUN06B45	23-8	m	0.358673	0.000017	1.620966	0.000075	0.059347	0.000004	9.0
JUN06B49	23-9	r	0.358811	0.000016	1.621465	0.000070	0.059381	0.000005	9.4
JUL23B14	24-1	r	0.359456	0.000009	1.623632	0.000048	0.059555	0.000003	16.8
JUL23B15	24-2	m	0.359436	0.000010	1.623521	0.000052	0.059549	0.000004	16.6
JUL23B17	24-3	c	0.359308	0.000012	1.623116	0.000054	0.059518	0.000003	17.4
JUL23B18	24-4	c	0.359305	0.000009	1.623133	0.000045	0.059516	0.000003	17.7
JUL23B20	24-5	m	0.359331	0.000010	1.623226	0.000053	0.059524	0.000004	18.0
JUL23B21	24-6	r	0.359367	0.000013	1.623319	0.000062	0.059531	0.000003	17.7
JUL23B30	25-1	r	0.359423	0.000011	1.623473	0.000059	0.059548	0.000003	16.6
JUL23B31	25-2	m	0.359399	0.000011	1.623436	0.000051	0.059540	0.000003	16.5
JUL23B33	25-3	m	0.359412	0.000011	1.623414	0.000053	0.059543	0.000004	17.3
JUL23B34	25-4	c	0.359404	0.000014	1.623482	0.000054	0.059542	0.000004	17.2
JUL23B36	25-5	c	0.359369	0.000014	1.623330	0.000062	0.059535	0.000004	16.5
JUL23B37	25-6	m	0.359386	0.000010	1.623412	0.000042	0.059537	0.000003	15.7
JUL23B39	26-1	r	0.359468	0.000009	1.623671	0.000044	0.059558	0.000003	16.3
JUL23B40	26-2	r	0.359470	0.000010	1.623642	0.000048	0.059556	0.000003	16.5
JUL23B42	26-3	m	0.359445	0.000009	1.623584	0.000050	0.059549	0.000003	16.5
JUL23B43	26-4	c	0.359393	0.000012	1.623377	0.000060	0.059538	0.000004	16.3
JUL23B45	26-5	c	0.359420	0.000009	1.623527	0.000052	0.059542	0.000003	16.8
JUL23B46	26-6	m	0.359448	0.000012	1.623616	0.000052	0.059552	0.000003	16.5
JUL23B51	26-7	r	0.359453	0.000012	1.623598	0.000054	0.059552	0.000003	16.5
Tonalite D17T119									
JUL19C081	2-1	r	0.359743	0.000010	1.624693	0.000045	0.059624	0.000004	12.9
JUL19C082	2-2	m	0.359687	0.000010	1.624462	0.000064	0.059607	0.000003	12.7
JUL19C084	2-3	m	0.359667	0.000011	1.624412	0.000051	0.059603	0.000004	12.7
JUL19C085	2-4	c	0.359644	0.000012	1.624327	0.000064	0.059594	0.000003	12.5
JUL19C087	2-5	m	0.359666	0.000011	1.624371	0.000058	0.059602	0.000004	12.8
JUL19C088	2-6	m	0.359692	0.000013	1.624450	0.000064	0.059608	0.000004	12.7
JUL20A018	2-7	r	0.359926	0.000014	1.625283	0.000077	0.059668	0.000005	10.9
JUL20A019	5-1	r	0.359940	0.000013	1.625353	0.000063	0.059677	0.000004	11.1
JUL20A021	5-2	m	0.359956	0.000012	1.625343	0.000075	0.059677	0.000005	11.0
JUL20A022	5-3	c	0.359934	0.000013	1.625319	0.000068	0.059672	0.000004	11.1
JUL20A024	5-4	c	0.359956	0.000012	1.625401	0.000064	0.059680	0.000004	11.2
JUL20A025	5-5	c	0.359953	0.000011	1.625423	0.000060	0.059679	0.000004	11.1
JUL20A027	5-6	r	0.360009	0.000013	1.625602	0.000071	0.059690	0.000005	11.1
JUL20A034	14-1	r	0.360113	0.000012	1.625949	0.000060	0.059718	0.000004	12.0
JUL20A035	14-2	m	0.360018	0.000013	1.625573	0.000074	0.059693	0.000005	11.7
JUL20A037	14-3	c	0.360059	0.000014	1.625743	0.000070	0.059699	0.000004	11.5
JUL20A038	14-4	c	0.360025	0.000013	1.625616	0.000068	0.059699	0.000004	11.5
JUL22A006	14-5	r	0.359675	0.000009	1.624353	0.000053	0.059608	0.000003	14.3
JUL22A007	15-1	r	0.359684	0.000011	1.624423	0.000051	0.059610	0.000003	14.9
JUL22A009	15-2	m	0.359628	0.000009	1.624161	0.000051	0.059597	0.000003	14.8

$\delta^{94/90}Zr_{GJ-1}$	2SE	$\delta^{94/91}Zr_{GJ-1}$	2SE	$\delta^{96/90}Zr_{GJ-1}$	2SE	$\delta^{94}Zr = \delta^{94/90}Zr_{IPGP-Zr}$ ‡	2SE
0.02	0.10	-0.01	0.09	0.02	0.14	0.01	0.10
-0.32	0.11	-0.26	0.09	-0.58	0.15	-0.33	0.12
-0.46	0.11	-0.37	0.09	-0.76	0.15	-0.47	0.11
-0.08	0.10	-0.04	0.09	-0.16	0.15	-0.09	0.11
						-0.09±0.06 (2SE)	
0.00	0.11	-0.06	0.09	0.00	0.16	-0.02	0.11
0.03	0.11	-0.02	0.09	0.06	0.17	0.02	0.12
0.01	0.11	-0.05	0.09	-0.04	0.17	0.00	0.11
0.19	0.11	0.14	0.09	0.26	0.18	0.18	0.12
0.03	0.11	0.00	0.09	0.13	0.17	0.01	0.11
-0.04	0.11	-0.06	0.09	0.00	0.16	-0.05	0.11
0.23	0.11	0.13	0.09	0.31	0.17	0.22	0.11
0.26	0.10	0.14	0.09	0.31	0.15	0.25	0.11
0.25	0.10	0.14	0.09	0.28	0.15	0.24	0.11
0.09	0.11	-0.01	0.09	0.18	0.17	0.08	0.12
0.17	0.11	0.07	0.10	0.25	0.15	0.16	0.11
-0.13	0.11	-0.13	0.10	-0.18	0.14	-0.14	0.11
-0.06	0.11	-0.10	0.10	0.04	0.15	-0.07	0.12
0.00	0.11	-0.06	0.10	0.01	0.15	-0.01	0.11
-0.13	0.11	-0.15	0.10	-0.07	0.15	-0.14	0.12
-0.30	0.11	-0.24	0.09	-0.45	0.16	-0.31	0.12
-0.23	0.11	-0.21	0.09	-0.33	0.16	-0.24	0.11
-0.01	0.11	-0.04	0.09	-0.04	0.16	-0.02	0.12
0.35	0.11	0.24	0.10	0.47	0.18	0.34	0.12
0.08	0.08	0.03	0.07	0.11	0.11	0.07	0.09
0.02	0.09	-0.04	0.07	0.02	0.12	0.01	0.10
-0.21	0.09	-0.18	0.07	-0.32	0.11	-0.22	0.10
-0.22	0.09	-0.17	0.07	-0.35	0.11	-0.23	0.10
-0.09	0.09	-0.07	0.07	-0.16	0.12	-0.10	0.10
0.01	0.09	-0.01	0.07	-0.05	0.12	0.00	0.10
-0.16	0.09	-0.17	0.08	-0.28	0.12	-0.17	0.10
-0.23	0.09	-0.20	0.07	-0.43	0.12	-0.24	0.10
-0.11	0.10	-0.14	0.07	-0.18	0.14	-0.12	0.10
-0.13	0.10	-0.10	0.07	-0.21	0.14	-0.14	0.11
-0.16	0.10	-0.13	0.08	-0.23	0.14	-0.17	0.10
-0.11	0.09	-0.08	0.08	-0.21	0.13	-0.12	0.10
0.11	0.09	0.07	0.07	0.14	0.11	0.10	0.10
0.11	0.09	0.05	0.07	0.11	0.11	0.10	0.10
0.05	0.08	0.00	0.06	0.01	0.12	0.04	0.09
-0.10	0.09	-0.13	0.07	-0.17	0.12	-0.11	0.10
-0.02	0.08	-0.03	0.06	-0.09	0.12	-0.03	0.09
0.06	0.09	0.02	0.06	0.07	0.11	0.04	0.10
-0.07	0.09	-0.09	0.07	-0.19	0.12	-0.08	0.10
						-0.02±0.05 (2SE)	
0.02	0.09	-0.01	0.07	-0.01	0.13	0.01	0.10
-0.14	0.09	-0.15	0.07	-0.29	0.13	-0.15	0.10
-0.20	0.10	-0.19	0.08	-0.37	0.13	-0.21	0.10
-0.26	0.10	-0.24	0.08	-0.51	0.13	-0.27	0.11
-0.17	0.10	-0.20	0.08	-0.32	0.14	-0.18	0.11
-0.10	0.10	-0.15	0.08	-0.21	0.14	-0.11	0.11
0.00	0.11	-0.03	0.09	-0.12	0.17	-0.01	0.11
0.04	0.10	0.01	0.09	0.02	0.16	0.03	0.11
0.00	0.10	-0.06	0.09	-0.11	0.17	-0.02	0.11
-0.07	0.10	-0.07	0.09	-0.18	0.16	-0.08	0.11
-0.11	0.09	-0.10	0.08	-0.20	0.14	-0.12	0.10
-0.12	0.09	-0.09	0.08	-0.21	0.14	-0.13	0.10
-0.04	0.09	-0.02	0.09	-0.16	0.15	-0.05	0.10
0.09	0.09	0.03	0.09	0.15	0.14	0.08	0.10
-0.17	0.09	-0.20	0.09	-0.27	0.14	-0.18	0.10
-0.07	0.10	-0.10	0.10	-0.25	0.14	-0.08	0.11
-0.16	0.10	-0.18	0.10	-0.24	0.14	-0.18	0.11
0.12	0.10	0.05	0.08	0.10	0.13	0.10	0.10
0.14	0.10	0.09	0.08	0.14	0.13	0.13	0.11
-0.07	0.10	-0.12	0.08	-0.13	0.14	-0.08	0.11

Analysis No.	Spot No. *	Domain †	$^{94}Zr/^{90}Zr$	2SE	$^{94}Zr/^{91}Zr$	2SE	$^{96}Zr/^{90}Zr$	2SE	^{90}Zr (V)
JUL22A010	15-3	m	0.359639	0.000010	1.624248	0.000052	0.059606	0.000003	14.7
JUL22A012	15-4	c	0.359626	0.000010	1.624210	0.000058	0.059596	0.000003	14.4
JUL22A013	15-5	c	0.359623	0.000012	1.624149	0.000057	0.059600	0.000003	14.8
JUL22A015	15-6	c	0.359609	0.000010	1.624140	0.000057	0.059592	0.000003	14.7
JUL22A016	15-7	m	0.359629	0.000012	1.624166	0.000060	0.059601	0.000003	14.5
JUL22A018	15-8	m	0.359683	0.000010	1.624407	0.000055	0.059609	0.000003	14.8
JUL22A019	15-9	r	0.359726	0.000009	1.624527	0.000052	0.059618	0.000003	14.8
JUL22A021	16-1	r	0.359729	0.000010	1.624540	0.000053	0.059622	0.000003	14.8
JUL22A022	16-2	m	0.359736	0.000009	1.624569	0.000053	0.059622	0.000003	14.2
JUL22A027	16-3	m	0.359728	0.000009	1.624606	0.000053	0.059623	0.000003	14.2
JUL22A028	16-4	c	0.359678	0.000010	1.624370	0.000053	0.059611	0.000003	14.3
JUL22A030	16-5	c	0.359677	0.000009	1.624381	0.000049	0.059608	0.000003	14.9
JUL22A031	16-6	m	0.359722	0.000012	1.624512	0.000060	0.059617	0.000004	14.5
JUL22A033	16-7	r	0.359723	0.000010	1.624533	0.000052	0.059617	0.000003	14.4
JUL22A034	17-1	r	0.359754	0.000010	1.624624	0.000054	0.059626	0.000003	14.3
JUL22A036	17-2	m	0.359679	0.000010	1.624351	0.000042	0.059613	0.000003	14.7
JUL22A037	17-3	m	0.359685	0.000015	1.624404	0.000089	0.059608	0.000005	14.5
JUL22A039	17-4	c	0.359671	0.000010	1.624375	0.000050	0.059607	0.000003	14.7
JUL22A040	17-5	c	0.359702	0.000012	1.624496	0.000061	0.059614	0.000003	14.6
JUL22A042	17-6	m	0.359740	0.000009	1.624575	0.000054	0.059624	0.000003	14.5
JUL22A043	17-7	m	0.359740	0.000010	1.624520	0.000052	0.059627	0.000003	14.5
JUL22A048	17-8	m	0.359772	0.000010	1.624729	0.000055	0.059632	0.000003	14.6
JUL22A049	17-9	r	0.359877	0.000019	1.625062	0.000071	0.059657	0.000005	14.6
Biotite-rich enclave D17T109									
JUN06A48	24-1	r	0.358511	0.000011	1.620415	0.000061	0.059310	0.000004	11.0
JUN06A50	24-2	c	0.358428	0.000012	1.620139	0.000071	0.059290	0.000004	10.3
JUN06A51	24-3	c	0.358438	0.000013	1.620165	0.000070	0.059292	0.000003	9.8
JUN06A52	24-4	m	0.358433	0.000018	1.620140	0.000081	0.059285	0.000004	9.8
JUN06A56	24-5	r	0.358581	0.000012	1.620703	0.000054	0.059322	0.000004	11.0
JUN06A57	28-1	c	0.358416	0.000014	1.620124	0.000070	0.059285	0.000004	11.5
JUN06A58	28-2	c	0.358454	0.000013	1.620207	0.000061	0.059298	0.000005	11.2
JUN06A60	28-3	m	0.358479	0.000014	1.620312	0.000061	0.059301	0.000005	10.4
JUN06A61	28-4	r	0.358544	0.000013	1.620549	0.000060	0.059318	0.000004	10.9
JUN06A42	25-1	c	0.358394	0.000013	1.620055	0.000058	0.059278	0.000003	12.5
JUN06A43	25-2	c	0.358377	0.000014	1.619962	0.000066	0.059281	0.000004	11.9
JUN06A44	25-3	m	0.358403	0.000011	1.619986	0.000058	0.059280	0.000004	11.8
JUN06A46	25-4	m	0.358476	0.000011	1.620281	0.000063	0.059303	0.000004	11.2
JUN06A47	25-5	r	0.358616	0.000016	1.620785	0.000074	0.059333	0.000006	12.2
JUN06A62	31-1	c	0.358298	0.000010	1.619692	0.000056	0.059256	0.000004	11.3
JUN06A64	31-2	c	0.358268	0.000011	1.619554	0.000062	0.059248	0.000003	11.5
JUN06A66	32-1	r	0.358623	0.000014	1.620762	0.000054	0.059334	0.000004	11.2
JUN06A70	32-2	m	0.358533	0.000013	1.620474	0.000067	0.059311	0.000003	10.7
JUN06A71	32-3	c	0.358476	0.000012	1.620272	0.000056	0.059297	0.000004	11.0
JUN06A72	32-4	c	0.358522	0.000014	1.620437	0.000072	0.059310	0.000004	10.3
JUN06A74	32-5	c	0.358481	0.000014	1.620283	0.000069	0.059301	0.000004	10.9
JUN06A75	32-6	m	0.358483	0.000012	1.620314	0.000067	0.059304	0.000003	10.7
JUN06A76	32-7	r	0.358745	0.000010	1.621198	0.000048	0.059362	0.000004	11.2
JUN06A78	33-1	c	0.358633	0.000012	1.620794	0.000066	0.059343	0.000004	9.7
JUN06A79	33-2	c	0.358547	0.000011	1.620573	0.000056	0.059320	0.000004	10.7
JUN06A80	34-1	c	0.358653	0.000014	1.620951	0.000066	0.059345	0.000004	10.0
JUN06A84	35-1	c	0.358384	0.000011	1.619945	0.000072	0.059276	0.000003	10.6

* Grain number and spot number. † c = core, m = mantle, and r = rim. ‡ The $\delta^{94}Zr$ of GJ-1 relative to the IPGP-Zr standard is -0.012 ± 0.042‰ (2SD).

$\delta^{94/90}Zr_{GJ-1}$	2SE	$\delta^{94/91}Zr_{GJ-1}$	2SE	$\delta^{96/90}Zr_{GJ-1}$	2SE	$\delta^{94}Zr = \delta^{94/90}Zr_{IPGP-Zr}$ ‡	2SE
-0.04	0.10	-0.07	0.08	0.02	0.14	-0.05	0.11
-0.13	0.10	-0.15	0.08	-0.24	0.14	-0.14	0.11
-0.13	0.10	-0.19	0.08	-0.17	0.14	-0.15	0.11
-0.26	0.10	-0.24	0.10	-0.48	0.13	-0.27	0.11
-0.21	0.10	-0.23	0.10	-0.34	0.13	-0.22	0.11
-0.09	0.11	-0.07	0.10	-0.22	0.14	-0.10	0.11
0.03	0.11	0.00	0.10	-0.07	0.14	0.02	0.11
0.06	0.07	0.04	0.03	0.06	0.06	0.05	0.08
0.09	0.07	0.05	0.03	0.07	0.06	0.07	0.08
0.13	0.11	0.11	0.09	0.15	0.16	0.12	0.12
-0.01	0.11	-0.04	0.09	-0.04	0.15	-0.02	0.12
-0.04	0.11	-0.04	0.09	-0.11	0.16	-0.05	0.11
0.09	0.11	0.04	0.09	0.04	0.16	0.08	0.12
0.02	0.09	0.00	0.09	-0.04	0.15	0.01	0.10
0.10	0.09	0.05	0.09	0.12	0.15	0.09	0.10
-0.11	0.09	-0.13	0.08	-0.09	0.14	-0.12	0.10
-0.10	0.09	-0.10	0.09	-0.19	0.16	-0.11	0.10
-0.15	0.09	-0.14	0.08	-0.25	0.13	-0.16	0.10
-0.07	0.09	-0.06	0.08	-0.14	0.13	-0.08	0.10
-0.04	0.10	-0.08	0.09	-0.15	0.14	-0.06	0.11
-0.04	0.10	-0.11	0.09	-0.11	0.14	-0.06	0.11
-0.03	0.11	-0.02	0.10	-0.12	0.16	-0.04	0.11
0.26	0.11	0.19	0.10	0.30	0.17	0.25	0.12
						-0.06±0.03 (2SE)	
-0.14	0.12	-0.13	0.10	-0.26	0.20	-0.15	0.12
-0.37	0.11	-0.30	0.10	-0.59	0.18	-0.38	0.12
-0.34	0.11	-0.28	0.10	-0.56	0.17	-0.35	0.12
-0.35	0.12	-0.30	0.11	-0.67	0.18	-0.37	0.13
0.04	0.13	0.07	0.10	-0.05	0.18	0.03	0.13
-0.42	0.13	-0.29	0.10	-0.67	0.18	-0.43	0.13
-0.31	0.13	-0.24	0.10	-0.45	0.18	-0.32	0.13
-0.28	0.12	-0.23	0.09	-0.46	0.18	-0.29	0.12
-0.10	0.12	-0.09	0.09	-0.17	0.18	-0.11	0.12
-0.45	0.13	-0.33	0.11	-0.77	0.19	-0.46	0.13
-0.49	0.13	-0.39	0.11	-0.73	0.20	-0.51	0.13
-0.42	0.12	-0.37	0.11	-0.74	0.20	-0.43	0.13
-0.24	0.12	-0.21	0.10	-0.37	0.19	-0.25	0.12
0.15	0.12	0.10	0.10	0.13	0.21	0.14	0.13
-0.79	0.11	-0.61	0.09	-1.23	0.18	-0.80	0.12
-0.85	0.11	-0.70	0.10	-1.28	0.16	-0.86	0.12
0.14	0.11	0.04	0.10	0.16	0.17	0.13	0.12
-0.14	0.11	-0.17	0.10	-0.30	0.17	-0.15	0.12
-0.30	0.11	-0.30	0.10	-0.52	0.17	-0.31	0.12
-0.17	0.12	-0.20	0.10	-0.32	0.18	-0.19	0.12
-0.40	0.11	-0.37	0.10	-0.68	0.16	-0.42	0.12
-0.40	0.11	-0.35	0.10	-0.64	0.16	-0.41	0.12
0.33	0.11	0.19	0.10	0.34	0.16	0.32	0.12
-0.04	0.11	-0.09	0.10	-0.06	0.17	-0.05	0.12
-0.28	0.11	-0.22	0.10	-0.45	0.16	-0.29	0.12
0.02	0.11	0.01	0.10	-0.01	0.16	0.01	0.12
-0.74	0.10	-0.61	0.09	-1.26	0.15	-0.75	0.11
						-0.28±0.11 (2SE)	

Table S5

In-situ LA-ICP-MS major and trace element profile data of zircons in plutonic arc rocks from southern Tibet.

Spot No.	Domain *	Zr Spot †	$\delta^{94}Zr$ †	Zr	Hf	Zr/Hf	Th	U	Th/U	Ti	T (°C) ‡
Hornblende gabbro D17T107											
3-1	r	3-1	0.04	479207	8627	56	385	419	0.92	9.9	814
3-2	r	3-1	0.04	475476	8781	54	281	278	1.0	6.3	771
3-3	m	3-2	0.05	463084	7591	61	472	332	1.4	11.3	828
3-4	m	3-3	0.15	473116	7889	60	401	319	1.3	17.3	874
3-5	c	3-4	0.07	477394	8308	57	633	468	1.4	6.7	777
3-6	m	3-5	0.06	475910	8155	58	419	346	1.2	9.6	811
3-7	m	3-6	0.06	474960	8072	59	504	360	1.4	11.4	829
3-8	r	3-7	0.00	477456	8300	58	890	643	1.4	9.1	806
6-1	-	6-2	0.19	475343	7456	64	2830	928	3.1	12.4	838
6-2	-	6-3	0.21	476809	7591	63	3279	1087	3.0		
6-3	-	6-4	0.39	467831	7705	61	3051	1111	2.7	14.8	857
8-1	r	8-1	0.28	473036	8684	54	1289	1255	1.0	12.5	839
8-2	r	8-2	0.25	473112	8164	58	1012	675	1.5	9.1	806
8-3	m	8-3	0.27	467385	8052	58	808	561	1.4	11.8	833
8-4	m	8-4	0.19	474444	7696	62	551	391	1.4	10.9	825
8-5	r	8-6	0.28	474496	8023	59	931	653	1.4	10.5	820
13-1	r	13-1	0.28	478690	7432	64	342	274	1.2	6.6	775
13-2	m	13-2	0.20	479062	6931	69	174	154	1.1	9.9	815
13-3	m	13-3	0.21	476463	6928	69	159	142	1.1	13.0	843
13-4	m	13-4	0.19	466373	6760	69	282	200	1.4	17.2	874
13-5	r	13-5	0.15	471476	6881	69	369	288	1.3	11.1	826
14-1	r	14-1	-0.11	467450	7486	62	161	135	1.2	14.8	857
14-2	m	14-3	-0.11	470919	7399	64	166	148	1.1	13.3	846
14-3	r	14-5	0.03	466808	8976	52	480	374	1.3	11.4	829
14-4	r	14-6	0.13	476490	8612	55	343	380	0.90	11.7	832
15-1	c	15-1	0.03	479993	7376	65	1019	605	1.7	11.7	831
15-2	m	15-3	0.01	472548	7317	65	1103	638	1.7	9.0	805
15-3	m	15-4	0.00	476181	7297	65	1224	686	1.8	12.1	836
15-4	r	15-5	0.14	458607	7171	64	1024	665	1.5		
15-5	r	15-6	0.07	479248	7583	63	549	511	1.1		
16-1	r	16-2	0.14	484313	7723	63	937	548	1.7	15.1	859
16-2	c	16-3	0.14	480793	7490	64	1146	638	1.8	13.5	847
16-3	c	16-4	0.15	480685	7697	62	1225	689	1.8	12.7	841
16-4	r	16-6	0.11	487564	7572	64	1322	702	1.9	15.7	864
Median ± 2SE											831±9
Hornblende gabbro D17T100											
3-1	r	3-1	0.04	479322	8142	59	467	423	1.1	7.9	801
3-2	m	3-3	-0.16	476174	7937	60	719	581	1.2	10.9	835
3-3	m-r	3-4	-0.15	484580	7683	63	216	238	0.91	4.8	755
3-4	m	3-5	-0.32	479428	6650	72	771	527	1.5	7.9	802
3-5	m	3-6	-0.24	479672	6759	71	726	518	1.4	9.6	821
3-6	r	3-7	-0.07	475714	6557	73	958	544	1.8	9.9	824
11-1	m	11-2	-0.16	479684	8480	57	494	480	1.0	7.9	801
11-2	m	11-5	-0.17	474472	8211	58	360	382	0.94	9.0	814
11-3	r	11-6	0.15	476033	8906	53	991	748	1.3	8.1	804
12-1	r	12-6	0.04	481541	7004	69	312	240	1.3	4.1	740
12-2	r	12-5	-0.01	478176	6952	69	330	251	1.3	6.2	778
12-3	m	12-2	0.10	476670	6725	71	585	300	1.9	8.4	807
12-4	r	12-1	0.19	479066	6759	71	606	304	2.0	7.4	795
13-1	r	13-1	0.07	482450	7969	61	212	210	1.0	4.4	746
13-2	m	13-2	0.00	483334	7126	68	187	200	0.93	10.0	825
13-3	c	13-3	-0.13	475980	6493	73	285	208	1.4	11.8	842
13-4	m	13-4	-0.21	480565	6531	74	224	177	1.3	8.6	810
13-5	r	13-5	0.10	482290	7611	63	319	277	1.2	6.7	785
15-1	r	15-1	-0.04	479598	7249	66	297	222	1.3		
15-2	m	15-2	-0.09	480265	6021	80	544	332	1.6	6.9	789
15-3	c	15-3	0.09	485214	6215	78	507	343	1.5	8.1	804
15-4	c	15-4	0.09	473961	5931	80	437	299	1.5	5.6	769
15-5	c	15-5	0.03	478164	6059	79	481	319	1.5	9.5	820
15-6	m	15-6	0.06	480164	5956	81	476	301	1.6	9.4	819
15-7	m	15-6	0.06	482153	5968	81	432	278	1.6	7.1	791
15-8	r	15-7	0.05	483469	6202	78	690	424	1.6	9.3	818

La	Ce	Pr	Nd	Sm	Eu	Gd	Tb	Dy	Ho	Er	Tm	Yb	Lu
0.29	16.8	0.08	0.89	2.3	0.96	12.2	3.9	49.4	20.0	108	24.6	261	61.7
0.01	11.1	0.05	0.47	1.0	0.66	9.0	3.3	40.3	16.9	87.9	20.2	209	50.5
0.01	14.3	0.08	1.2	2.9	1.6	18.6	6.1	70.1	27.4	135	29.2	296	67.9
0.16	11.8	0.27	3.3	6.4	2.5	26.8	7.5	79.6	29.5	146	31.5	314	74.0
0.03	13.00	0.28	4.1	6.3	2.6	27.2	7.8	85.1	30.4	148	31.8	323	74.0
0.03	10.4	0.18	2.5	4.0	1.8	19.2	5.4	62.9	23.3	116	25.7	255	60.4
0.03	13.1	0.10	1.3	3.3	1.5	20.0	6.0	76	28.8	145	31.0	305	71.3
0.00	27.8	0.09	1.3	3.8	1.9	24.2	9.0	112	43.6	221	48.1	477	111
0.16	62.4	1.5	27.1	43.5	16.6	135	34.7	334	105	457	89.5	800	167
0.15	56.1	1.2	20.1	38	15.8	134.9	33.8	332	104	448	88.8	797	164
0.09	41.9	0.81	13	26	12.6	105.5	27.1	278	89.0	392	78.1	705	149
0.01	27.2	0.07	1.6	2.9	1.5	18.8	6.5	89.3	37.2	192	42.0	420	98.8
0.07	17.9	0.44	5.3	8.9	4.2	40.7	11.3	126	45.5	214	45.6	437	98.6
0.06	17.9	0.42	5.4	9.5	4.3	37.7	10.8	121	41.2	202	41.2	398	89.0
0.04	13.7	0.26	3.5	7.1	3.0	27.6	7.9	86.9	31.2	151	31.8	304	69.1
0.05	16.6	0.28	4.9	8.1	3.5	35.1	9.8	112	39.7	192	41.1	400	90.3
0.02	8.7	0.17	2.9	4.1	1.8	17.0	4.9	56.4	20.3	98.8	22.0	215	50.2
0.01	6.9	0.13	2.2	3.4	1.4	13.1	3.9	44.7	15.5	78.4	17.2	172	41.1
0.03	6.6	0.13	1.8	2.9	1.3	12.0	3.8	40.6	15.4	77.0	16.7	166	40.1
0.04	10.1	0.28	3.9	5.5	2.5	21.8	6.0	67.5	23.3	111	25.1	241	55.2
0.04	10.6	0.29	3.9	5.9	2.3	23.9	6.5	74.4	26.0	125	26.1	263	61.5
0.03	8.6	0.22	3.3	5.2	1.9	23.7	7.0	80.7	28.2	137	28.6	280	62.7
0.02	9.0	0.14	2.6	4.8	1.5	20.4	6.4	74.1	27.2	134	29.3	283	66.4
0.04	12.1	0.27	4.3	5.0	2.1	25.0	6.8	79.9	29.2	140	30.6	298	68.1
0.00	12.4	0.12	1.2	2.7	1.1	13.4	4.3	53	21.9	117	25.9	275	66.0
0.06	20.7	0.38	5.7	9.7	4.4	48.1	14.2	156	57.2	258	51.5	488	106
0.06	21.5	0.43	5.9	11.4	4.6	49.9	14.8	165	59.3	269	53.9	506	110
0.03	23.6	0.50	7.0	12.4	5.2	55.4	15.8	174	62.6	285	57.1	531	117
1.3	23.8	0.24	2.4	3.8	1.9	23.9	8.9	113	47.3	226	45.2	429	96.0
0.00	14.2	0.09	1.0	2.2	1.1	13.8	5.0	67.6	30.0	149	30.9	314	74.2
0.03	24.8	0.45	8.0	11.4	4.7	40.6	11.9	121	45.1	208	42.7	417	93.2
0.07	29.3	0.62	10.2	15.3	6.2	53.5	14.7	152	53.8	246	50.3	491	110
0.07	30	0.69	10.7	15.6	6.0	55.1	14.7	154	55.2	247	50.8	498	111
0.06	32.1	0.60	9.9	14.3	5.8	54.1	15.3	159	58.3	265	53.9	520	115
0.00	9.7	0.03	0.62	1.9	0.8	7.7	2.7	32.7	12.8	68.2	14.8	147	35.7
0.00	21.2	0.05	1.2	3.3	1.9	23.6	9.4	114	47.0	245	53.8	535	125
0.01	7.6	0.04	0.74	2.2	1.2	12.2	4.0	48.4	21.0	117	27.2	283	70.6
0.03	18.2	0.27	5.4	9.1	4.0	39.1	11.6	135	51.1	259	55.2	549	127
0.02	17.5	0.28	4.5	8.8	4.3	39.9	11.7	134	51.9	262	55.5	553	130
0.02	19.4	0.23	4.7	10	5.4	50.9	14.8	159	59.0	291	59.6	574	131
0.01	28.1	0.07	1.6	3.0	1.8	21.0	7.8	101	40.4	220	48.3	497	118
0.01	21.7	0.09	1.4	3.3	1.7	19.1	6.9	85.6	34.9	191	42.4	444	106
0.01	42.4	0.10	1.6	3.3	1.8	23.6	8.7	113	46.9	246	55.2	559	132
0.01	5.8	0.11	1.6	3.1	1.6	12.9	3.6	42.5	15.2	77.0	17.0	171	39.9
0.01	5.4	0.11	2.0	3.5	1.7	13.3	3.8	44.6	16.0	78.9	17.4	173	41.9
0.02	14.8	0.37	4.9	9.9	5.0	36.5	10.2	97.9	34.2	161	32.9	317	70.9
0.02	14.4	0.31	5.2	9.7	5.5	38.6	10.0	106	36.1	167	34.5	336	74.5
0.00	7.4	0.01	0.69	1.4	0.74	10.2	3.7	48.5	20.0	110	24.7	256	62.0
0.00	9.6	0.03	1.2	1.9	1.1	12.4	4.1	51.7	21.9	115	25.9	262	64.4
0.01	12.1	0.03	1.2	2.8	1.8	18.0	5.8	67.5	26.9	135	29.5	289	68.9
0.01	9.8	0.07	1.4	2.7	1.7	14.7	5.1	59.3	22.9	117	25.9	262	63.2
0.00	8.2	0.04	0.78	2.4	1.5	16.7	6.0	80.7	32.4	167	37.2	371	85.0
0.06	10.2	0.15	2.2	3.5	2.3	17.5	4.6	55.2	20.7	105	22.8	217	49.9
0.04	14.3	0.30	4.7	8.8	5.4	38.4	10.5	111	39.5	187	38.2	379	86.1
0.05	12.4	0.33	4.0	7.5	4.5	32	8.8	92.0	33.6	166	34.9	341	80.0
0.06	11.9	0.29	4.2	7.8	4.8	31.8	8.7	93.2	32.5	159	33.3	323	74.7
0.04	13.0	0.28	4.7	8.1	4.8	34	9.4	98.3	35.1	166	35.0	346	79.0
0.07	14.6	0.29	5.0	8.1	4.4	34.4	9.3	98.1	34.8	165	34.5	338	76.8
0.03	13.1	0.29	4.4	8.0	4.6	31.7	8.7	90.1	32.3	157	32.8	320	75.1
0.04	18.7	0.21	3.5	6.3	4.0	27.9	8.9	99.7	37.7	182	38.6	381	87.1

Median ± 2SE											804±11
Hornblende gabbro D17T102											
1-1	r	1-1	0.10	479207	7534	64	525	347	1.5	5.3	751
10-1	m	10-1,2	-0.07	478422	7521	64	686	558	1.2	5.1	749
10-2	r	10-3	0.26	480769	8701	55	299	378	0.79	6.5	770
24-1	r	24-2,3	-0.25	477071	8548	56	1164	678	1.7	7.5	784
24-2	r	24-2,3	-0.25	478819	7992	60	522	376	1.4	6.7	773
38-1	r	38-1	-0.05	478912	8542	56	2080	1146	1.8	11.9	829
38-2	c	38-2	0.02	483541	7812	62	1290	905	1.4	8.2	792
38-3	r	38-3	0.13	482552	8320	58	499	430	1.2	4.5	738
41-1	r	41-1,6	0.20	485866	8323	58	131	198	0.66	4.5	738
41-2	m	41-2	0.00	484838	8150	59	451	364	1.2	4.6	738
43-1	r	43-1	0.00	477791	8689	55	523	456	1.1	4.3	734
43-2	c	43-2	-0.13	480454	7937	61	37	61	0.6	4.2	730
43-3	c	43-3,4	-0.16	479043	7805	61	156	117	1.3	4.8	743
43-4	r	43-5	-0.29	477512	7533	63	197	183	1.1	3.8	723
43-5	r	43-6	-0.26	482035	7650	63	96	112	0.85	3.1	707
Median ± 2SE											743±16
Tonalite D17T119											
2-1	r	2-1	0.01	480137	10397	46	236	448	0.53	3.9	714
2-2	m	2-3	-0.21	474618	9199	52	224	291	0.77	5.3	742
2-3	c	2-4	-0.27	479487	8803	54	175	236	0.74	3.8	713
2-4	m	2-6	-0.11	480582	9092	53	147	233	0.63		
2-5	r	2-7	-0.01	478624	9741	49	404	480	0.84	6.4	758
5-1	r	5-1	0.03	477294	8921	54	187	280	0.67	6.6	760
5-2	c	5-2	-0.02	481736	9107	53	105	210	0.50	4.2	722
5-3	c	5-4	-0.12	475098	8939	53	89	171	0.52		
5-4	r	5-6	-0.05	479273	9467	51	183	322	0.57	4.9	734
14-1	r	14-1	0.08	478019	10644	45	380	604	0.63	3.9	715
14-2	m	14-2	-0.18	479158	10255	47	285	380	0.75	3.9	715
14-3	c	14-3	-0.08	479485	9019	53	107	193	0.56	5.7	748
14-4	c	14-4	-0.18	474748	9714	49	171	264	0.65	3.6	708
14-5	m	14-3	-0.08	476409	9720	49	168	258	0.65	5.5	745
14-6	r	14-5	0.10	470822	9783	48	141	278	0.51	4.8	732
15-1	r	15-1	0.13	497526	10714	46	262	527	0.50	4.7	731
15-2	m	15-3	-0.05	488567	8107	60	130	217	0.60	9.8	798
15-3	c	15-4	-0.14	496245	8318	60	178	234	0.76	5.6	746
15-4	c	15-5	-0.15	495143	8205	60	227	280	0.81	3.8	714
15-5	c	15-6	-0.27	489249	8136	60	167	231	0.72	6.4	758
15-6	m	15-7	-0.22	495016	8128	61	220	282	0.78	9.2	792
15-7	r	15-8	-0.10	491426	9200	53	286	393	0.73	5.9	750
15-8	r	15-9	0.02	493782	9103	54	237	294	0.81	4.1	719
16-1	r	16-7	0.01	494383	8681	57	345	425	0.81	6.2	755
16-2	m	16-6	0.08	495630	8513	58	127	231	0.55	4.2	721
16-3	c	16-4	-0.02	493727	8035	61	132	188	0.70	4.4	726
16-4	m	16-3	0.12	496521	8027	62	162	240	0.68	6.2	755
16-5	r	16-1	0.05	494234	8540	58	446	484	0.92	7.0	766
17-1	r	17-1	0.09	494421	8938	55	170	313	0.54	4.8	733
17-2	m	17-3	-0.11	493061	8117	61	112	219	0.51	6.4	758
17-3	c	17-5	-0.08	494674	8356	59	245	264	0.93	8.9	789
17-4	c	17-6	-0.06	498862	8406	59	182	204	0.89	7.0	767
17-5	m	17-7	-0.06	499903	8448	59	184	236	0.78	6.0	752
17-6	m	17-8	-0.04	497469	8526	58	126	237	0.53	3.8	712
17-7	r	17-9	0.25	498383	9449	53	186	330	0.56	3.0	695
Median ± 2SE											742±9
Biotite-rich enclave D17T109											
24-1	r	24-4	0.07	472910	10414	45	181	331	0.55	5.7	726
24-2	r	24-2	-0.38	475309	10270	46	159	288	0.55	4.7	709
28-1	m	28-1,2	0.07	469976	10247	46	240	327	0.74	4.9	713
28-2	c	28-2	-0.32	474145	11416	42	104	228	0.46	3.3	680
30-1	c	30-1	-0.36	473266	10247	46	154	234	0.66	3.6	689
30-2	r	30-4	-0.05	472327	11653	41	207	363	0.57	3.6	689
32-1	r	32-1	0.13	464730	9861	47	295	383	0.77	6.0	730
32-2	m	32-4	-0.19	482172	9777	49	176	256	0.69	2.6	663

0.00	17.7	0.03	0.73	2.1	1.2	9.9	3.5	41.8	16.6	84.6	19.4	183	45.1
0.02	34.3	0.12	2.0	4.1	2.4	24.6	8.5	102	39.8	210	48.6	469	118
0.01	10.8	0.03	0.35	0.95	0.56	5.9	2.2	29.2	13.1	77.7	19.9	218	62.8
0.00	37.4	0.07	1.5	3.0	1.7	14.5	4.5	53.4	19.6	103	24.2	237	61.8
0.01	23.9	0.05	1.1	2.2	1.2	10.9	3.9	47.7	18.8	99.5	23.2	233	61.1
0.03	95	0.17	3.2	7.2	3.0	32.4	9.8	120	44.8	240	56.2	537	133
0.04	90.9	0.37	6.6	11.5	5.1	54.2	18.1	204	76.8	397	91.3	861	209
0.01	19.4	0.05	0.74	1.2	0.69	5.6	2.3	26.6	10.9	62.9	15.6	166	44.4
0.00	13.1	0.04	0.43	1.1	0.63	5.7	2.4	29.4	12.0	71.8	18.3	190	51.8
0.00	17.9	0.12	2.5	4.4	1.9	16.5	5.3	60.8	22.1	116	27.6	269	68.6
0.02	28.2	0.25	3.6	4.7	2.6	21.7	7.1	81.7	30.6	161	37.5	370	93.5
0.01	3.9	0.01	0.32	0.92	0.38	3.9	1.3	15.2	5.5	30.4	7.1	72.8	18.6
0.00	8.4	0.08	0.96	1.9	1.2	9.9	3.2	36.5	12.7	64.7	14.3	131	32.9
0.02	10.9	0.14	2.1	4.1	1.9	15.7	4.7	52.2	18.7	98.9	22.4	213	53.4
0.00	6.4	0.09	1.6	2.4	1.4	10.1	3.0	34.4	12.8	62.8	14.4	135	32.8

0	26.4	0.03	0.89	1.8	1.1	13.9	5.3	66.6	28.4	151	34.7	356	86.4
4.4	39.4	2.80	13.3	4.2	1.2	11.8	3.9	43.2	16.7	84.2	18.2	183	43.8
0.02	19.7	0.13	2.3	4.3	2.1	19.0	6.1	69.9	27.7	140	30.7	322	79.3
0.07	22.6	0.09	0.84	2.3	1.3	14.7	5.1	64.4	27.4	141	30.9	325	80.6
0.02	46.1	0.12	1.9	5.8	3.0	32.7	11.2	130	51.4	250	53.1	539	128
0.01	24.2	0.06	1.5	3.1	1.6	19.2	6.4	80.2	33.6	169	37.5	389	94.3
0.00	19.1	0.06	0.99	2.3	1.1	13.0	4.3	56.0	24.0	128	28.7	304	75.6
0.02	13.9	0.07	0.78	1.8	0.95	10.7	3.9	45.9	19.9	106	24.5	258	65.6
0.00	25.5	0.08	1.2	3.3	1.4	17.4	6.1	78.6	33.4	172	38.7	407	98.8
0.00	38.9	0.03	1.0	3.4	1.5	21.0	7.7	96.5	41.5	217	46.8	479	115
0.01	28.6	0.04	1.1	2.4	1.3	14.4	4.7	60.3	23.9	121	26.6	273	65.2
0.00	17.2	0.05	0.88	2.2	1.3	11.5	4.1	53.6	22.8	118	26.8	279	68.9
0.07	23	0.08	1.5	2.5	1.3	15.2	5.3	64.1	26.4	140	30.9	323	78.3
0.08	21.9	0.09	1.2	2.5	1.2	13.3	4.7	57.7	24.1	129	28.8	294	74.6
0.00	23.6	0.07	1.3	2.5	1.4	14.2	5.3	69.9	29.4	152	34.8	350	85.9
0.07	23.3	0.09	1.0	1.9	0.82	12.2	4.5	52.9	21.3	112	27.0	281	68.4
0.00	19.4	0.06	1.3	2.1	1.4	14.6	4.8	59.6	24.6	127	29.9	312	77.1
0.05	19.7	0.09	1.2	2.7	1.5	15.5	5.0	62.8	24.5	129	30.6	316	78.8
0.01	23.3	0.15	3.0	5.2	2.4	24.6	7.5	83.3	30.9	160	36.9	382	93.6
0.02	19.0	0.08	1.1	2.7	1.5	13.4	4.9	58.4	23.9	131	29.8	317	79.1
0.01	26.4	0.06	1.3	3.0	1.7	17.1	6.0	74.0	28.9	149	33.9	345	86.7
0.00	31.9	0.09	1.3	3.2	1.3	18.7	6.3	76.6	31.9	167	38.8	413	101
0.07	21.2	0.09	1.1	1.8	0.89	11.3	3.4	40.8	15.5	77.9	18.0	181	44
0.01	34.4	0.05	1.6	3.6	1.9	22.2	7.5	89.4	34.0	172	39.6	397	97.4
0.01	22.1	0.09	0.92	2.5	1.1	12.8	5.0	61.8	25.4	138	31.9	327	81.6
0.01	16.2	0.15	2.5	3.8	2.1	19.4	5.8	63.7	24.6	120	27.3	276	68.7
0.03	21.2	0.08	1.0	2.6	1.1	14.7	5.1	62.2	25.4	133	30.7	310	78.5
0.00	39.2	0.07	1.8	3.9	1.9	25.8	8.6	96.4	37.8	198	44.6	440	108
0.00	27.8	0.07	0.96	2.4	1.4	17.2	6.0	78.3	31.9	172	39.5	400	99.1
0.00	17.9	0.03	0.73	2.1	1.1	12.9	4.4	58.3	23.1	125	29.3	301	77.5
0.01	23.7	0.11	1.5	2.9	1.5	20.2	6.2	73.6	28.0	139	31.7	315	77.2
0.00	18.1	0.09	1.7	3.3	1.6	17.0	5.5	64.4	24.0	122	27.4	279	69.1
0.00	21.7	0.04	1.0	2.0	1.3	16.3	5.2	62.0	25.3	133	30.6	321	80.0
0.01	22.1	0.04	1.1	2.1	1.2	13.8	4.9	64.2	26.2	145	33.0	339	86.5
0.00	24.9	0.05	0.89	2.5	1.2	14.7	5.4	66.0	27.2	147	34.2	359	89.6

0.00	28.4	0.1	1.7	2.6	1.5	19.5	6.8	80.9	34.4	182	42.0	424	105
0.00	23.4	0.05	1.3	2.6	1.3	16.6	5.9	73.9	30.4	163	37.9	388	95.9
0.00	27.8	0.04	0.78	2.1	1.1	13.2	4.9	54.6	21.2	110	24.4	246	59.5
0.00	14.8	0.03	0.46	1.2	0.63	8.6	3.0	39.3	16.3	91.1	21.8	234	59.6
0.00	18.7	0.12	2.0	3.8	1.7	17.8	5.7	64.0	25.0	131	30.5	310	77.3
0.00	22.1	0.04	0.68	1.9	0.81	11.4	4.1	47.3	19.8	108	25.5	267	66.1
11.1	63.8	3.6	16.6	6.1	2.2	23.1	7.9	90.7	35.5	184	40.4	397	95.4
0.01	20.9	0.04	1.3	2.7	1.3	15.2	5.4	63.5	25.7	137	31.6	321	79.7

33-1	r	-	-	487886	9946	49	125	211	0.59	3.2	679
33-2	c	33-1	-0.05	484270	9661	50	344	423	0.81	2.5	660
33-3	c	33-2	-0.29	482740	9497	51	422	518	0.81	4.6	709
34-2	m	34-1	0.01	476565	14220	34	2152	1742	1.2	3.6	689
35-1	r	35-1	-0.75	480005	10548	46	755	699	1.1	4.5	707
Median ± 2SE											689±12
Standard zircon 91500											
MAY12P06				479669	5712	84	20.6	60.1	0.34	4.6	
MAY12P22				486531	5779	84	20.1	58.3	0.34	3.9	
MAY12P38				486460	5767	84	21.5	59.9	0.36	3.9	
MAY12P54				476948	5930	80	20.0	58.6	0.34	4.0	
MAY12P70				479764	5751	83	19.7	58.2	0.34	4.0	
MAY12P81				489657	5803	84	19.4	57.9	0.34	3.8	
MAY12Q06				480041	6598	73	22.1	62.8	0.35	5.1	
MAY12Q22				489630	6312	78	23.6	65.0	0.36	6.1	
MAY12Q38				491371	6256	79	21.8	61.8	0.35	5.5	
MAY12Q54				485505	6170	79	24.7	67.9	0.36	4.7	
MAY12Q60				488730	6367	77	23.1	64.1	0.36	5.5	
MAY18T08				478238	5885	81	29.5	78.3	0.38	4.5	
MAY18T09				476755	5878	81	29.2	77.5	0.38	4.1	
MAY18T10				477561	5887	81	29.7	78.3	0.38	5.2	
MAY18T30				481301	5811	83	29.6	78.2	0.38	4.9	
MAY18T46				481201	5754	84	29.2	78.4	0.37	6.8	
MAY18T62				481924	5846	82	29.7	78.3	0.38	4.2	
MAY18T78				475533	6001	79	30.2	79.0	0.38	4.6	
MAY18T89				476454	5874	81	30.0	78.9	0.38	4.6	
MAY18U06				487541	5786	84	31.0	82.2	0.38	5.0	
MAY18U22				485134	5793	84	31.2	84.6	0.37	3.6	
MAY18U39				483883	5653	86	30.5	83.2	0.37	4.9	
MAY18U54				482308	5693	85	31.1	82.5	0.38	4.5	
MAY18U70				481620	5683	85	30.2	81.1	0.37	5.4	
MAY18U81				477754	5757	83	30.6	82.3	0.37	4.4	
MAY19P06				499204	5325	94	29.4	76.0	0.39	4.9	
MAY19P22				502034	5226	96	29.2	75.6	0.39	5.1	
MAY19P33				499891	5308	94	29.1	75.4	0.39	4.5	
Median ± 2SD				484380±14395	5843±603	83.1±10				4.7±1.4	
Ref. (6)				490611±1003	5857±235	84±3					
Ref. (8)										4.7±0.3	
Standard zirconGJ-1											
MAY12P05				478093	6992	68	8.6	291	0.03	2.9	
MAY12P21				478633	6827	70	8.5	286	0.03	3.6	
MAY12P37				481398	7022	69	8.9	284	0.03	3.6	
MAY12P53				478373	7175	67	8.7	288	0.03	2.0	
MAY12P69				477885	6918	69	8.7	287	0.03	4.1	
MAY12P80				482534	6857	70	8.5	280	0.03	3.9	
MAY12Q05				476334	7717	62	9.8	316	0.03	3.6	
MAY12Q21				481347	7548	64	9.4	309	0.03	3.0	
MAY12Q37				484362	7524	64	9.7	305	0.03	3.6	
MAY12Q53				482984	7587	64	9.7	313	0.03	4.4	
MAY12Q59				483236	7623	63	9.2	310	0.03	4.3	
MAY18T05				472050	6978	68	10.1	311	0.03	4.7	
MAY18T06				470929	6946	68	9.7	306	0.03	3.3	
MAY18T07				472801	7025	67	10.0	304	0.03	5.3	
MAY18T29				482274	6989	69	10.1	304	0.03	4.2	
MAY18T45				479356	6982	69	10.0	309	0.03	6.3	
MAY18T61				486908	7007	69	9.8	301	0.03	4.7	
MAY18T77				480584	7336	66	10.1	303	0.03	3.7	
MAY18T90				481526	7128	68	10.4	302	0.03	3.3	

0.00	11.6	0.02	0.26	1.3	0.71	6.0	2.1	26.6	12.1	64.6	15.0	159	39.5
0.00	22.3	0.06	0.69	1.9	1.6	12.9	4.3	54.6	23.1	126	29.2	298	74.0
0.01	27.2	0.08	1.2	2.4	1.8	14.5	5.3	64.8	27.6	155	36.5	366	90.3
0.02	73.6	0.24	4.8	7.7	5.0	36.2	12.0	142	58.9	297	64.8	607	139
0.01	39.3	0.13	2.5	4.6	3.1	25.2	8.3	93.4	38.3	195	43.9	437	107

0.00	2.3	0.02	0.10	0.30	0.22	2.1	0.79	11.0	4.4	26.0	6.6	63.2	14.8
0.00	2.5	0.01	0.27	0.31	0.26	1.8	0.77	10.4	4.3	25.4	6.3	62.4	14.1
0.00	2.4	0.00	0.14	0.38	0.18	2.1	0.88	11.9	4.9	26.1	6.6	67.2	15.3
0.00	2.3	0.00	0.23	0.37	0.17	2.3	0.77	11.2	4.5	25.8	6.2	63.0	14.6
0.00	2.2	0.01	0.19	0.46	0.25	2.4	0.85	10.2	4.3	25.1	6.3	62.5	14.0
0.01	2.3	0.01	0.18	0.46	0.17	2.0	0.72	10.5	4.2	25.0	6.2	62.1	13.8
0.00	2.4	0.01	0.04	0.31	0.21	2.0	0.79	9.7	4.3	24.2	6.2	62.0	13.3
0.00	2.3	0.02	0.16	0.34	0.22	2.5	0.83	10.9	4.6	26.4	6.5	67.3	14.3
0.00	2.1	0.02	0.07	0.31	0.23	2.2	0.80	10.0	4.2	24.6	6.0	61.8	13.6
0.00	2.5	0.02	0.20	0.36	0.27	2.2	0.85	11.4	4.9	27.9	6.8	69.9	15.0
0.00	2.5	0.02	0.18	0.30	0.18	2.3	0.85	10.3	4.4	26.2	6.4	66.4	14.0
0.00	2.6	0.01	0.36	0.39	0.28	2.6	0.85	12.1	5.1	29.2	7.0	72.6	15.1
0.00	2.6	0.01	0.32	0.42	0.24	2.6	0.88	12.6	5.2	28.8	6.9	70.8	14.9
0.00	2.5	0.02	0.14	0.48	0.24	2.4	0.90	11.9	5.1	28.5	6.8	70.8	15.1
0.00	2.6	0.01	0.20	0.41	0.32	2.8	1.0	12.4	4.9	29.8	6.8	71.1	15.3
0.00	2.6	0.01	0.14	0.35	0.20	2.7	0.92	12.2	5.1	28.1	6.8	69.9	14.8
0.00	2.6	0.01	0.24	0.58	0.29	2.6	0.87	12.2	4.9	28.7	6.9	71.5	15.2
0.00	2.8	0.01	0.22	0.65	0.25	2.6	0.96	12.8	5.3	28.9	6.7	71.6	15.5
0.00	2.7	0.02	0.32	0.52	0.20	2.3	0.88	11.8	5.2	29.2	7.0	70.7	15.1
0.01	2.7	0.01	0.19	0.48	0.24	2.4	0.88	12.2	5.5	28.7	6.7	69.5	15.4
0.00	2.6	0.02	0.25	0.44	0.27	2.0	1.0	12.3	5.2	28.8	6.7	69.2	15.0
0.00	2.8	0.00	0.29	0.50	0.24	2.3	0.91	12.5	5.2	28.6	6.7	69.0	14.9
0.00	2.6	0.01	0.12	0.53	0.28	2.4	0.92	11.3	5.3	28.2	6.8	68.5	14.7
0.00	2.8	0.00	0.37	0.53	0.24	2.4	0.93	11.7	5.2	28.6	6.7	70.3	14.9
0.00	2.7	0.02	0.14	0.54	0.25	2.5	0.83	12.7	5.1	29.5	6.8	67.4	15.0
0.00	2.6	0.02	0.14	0.41	0.23	2.3	1.0	11.7	4.8	26.6	6.7	68.4	14.9
0.00	2.7	0.01	0.17	0.46	0.25	2.2	0.88	11.3	4.6	26.8	6.7	67.4	14.6
0.00	2.5	0.02	0.28	0.31	0.20	2.4	0.90	11.9	4.7	26.5	6.9	69.3	14.6

0.00	17.2	0.02	0.49	1.7	0.94	7.0	2.1	21.4	7.0	32.4	7.0	62.6	14.9
0.00	16.4	0.03	0.59	1.7	0.88	7.0	2.1	21.9	7.2	32.7	6.7	63.3	14.1
0.00	16.5	0.04	0.61	1.6	1.0	6.7	2.1	20.9	7.0	32.9	6.8	61.7	14.6
0.00	17.1	0.02	0.63	1.4	1.0	7.3	2.1	22.0	7.3	34.1	7.3	63.9	14.9
0.00	16.7	0.01	0.71	1.7	1.0	7.1	2.2	20.7	7.2	33.2	7.0	62.6	14.6
0.00	16.3	0.02	0.52	1.4	0.90	6.7	2.0	20.6	6.7	31.6	6.7	61.3	14.7
0.00	16.8	0.02	0.42	1.6	1.0	7.6	2.1	20.9	7.1	33.7	7.2	64.5	14.4
0.00	16.8	0.02	0.51	1.6	1.1	6.6	2.2	20.5	6.9	32.3	6.9	63.2	13.7
0.01	16.1	0.03	0.63	1.4	0.95	6.8	1.9	20.3	6.7	32.1	6.8	61.9	14.0
0.00	16.6	0.03	0.63	1.3	1.0	7.1	2.1	20.8	6.9	33.9	6.8	63.7	13.8
0.00	16.5	0.03	0.79	1.6	1.0	7.1	2.0	20.0	7.0	33.8	6.8	63.5	13.8
0.00	16.1	0.03	0.60	1.5	1.1	6.9	2.0	21.0	7.2	33.8	7.0	65.7	14.1
0.00	16.2	0.02	0.74	1.3	1.0	7.6	2.1	21.5	7.4	34.2	7.1	66.0	14.3
0.00	16.0	0.03	0.49	1.3	1.0	7.6	1.9	21.1	7.0	33.5	6.5	63.8	14.1
0.00	16.1	0.02	0.46	1.5	0.78	6.9	2.1	21.0	7.5	33.9	6.8	65.3	14.4
0.00	15.4	0.04	0.55	1.6	0.90	6.2	2.0	21.6	7.1	33.4	6.9	65.6	14.1
0.01	15.8	0.03	0.54	1.5	0.85	7.6	2.2	21.9	7.2	33.2	6.9	64.9	14.3
0.01	16.1	0.02	0.58	1.4	0.89	7.3	2.1	21.2	7.4	34.6	7.1	66.1	14.6
0.01	16.4	0.03	0.65	1.3	1.0	6.8	2.1	21.9	7.3	33.9	6.9	68.0	14.1

MAY18U05	485605	6943	70	10.3	322	0.03	4.0
MAY18U21	485735	7033	69	10.3	319	0.03	4.2
MAY18U38	483213	6864	70	10.2	321	0.03	3.6
MAY18U53	480729	6969	69	10.0	327	0.03	3.9
MAY18U69	479045	6825	70	10.5	321	0.03	2.9
MAY18U82	478906	6874	70	10.5	319	0.03	3.4
MAY19P05	494146	6380	77	10.0	299	0.03	3.3
MAY19P21	497122	6296	79	10.1	300	0.03	3.5
MAY19P34	498234	6390	78	10.0	297	0.03	3.6
Average ± 2SD	481941±12913	7027±704	68.8±8.1	9.7±1.3	305±25		3.8±1.6
Ref. (9)		6681±113		9.8±0.3	284±27		3.4±0.4

* c = core, m = mantle, and r = rim. † Spot No. for Zr isotopes. Note that the spot sites for Zr isotopes and element compositions are not strictly the same.

0.00	16.3	0.02	0.68	1.6	1.1	7.0	2.1	21.8	7.3	33.1	7.0	65.2	14.1
0.00	16.5	0.01	0.57	1.4	1.1	6.9	2.1	20.7	7.2	32.8	6.7	64.2	14.4
0.01	15.8	0.01	0.64	1.3	0.89	7.5	2.2	21.6	7.5	33.4	6.7	63.6	14.2
0.01	16.2	0.04	0.36	1.5	1.0	6.8	2.2	21.6	7.6	34.3	6.9	65.1	14.4
0.01	16.2	0.02	0.62	1.5	1.0	7.1	2.0	21.1	7.2	33.6	6.8	63.3	14.3
0.01	16.5	0.02	0.55	1.8	1.1	6.7	2.1	21.7	7.4	33.7	6.7	63.8	14.1
0.01	15.7	0.04	0.34	1.4	0.88	6.9	2.1	19.8	6.7	30.5	6.8	63.9	13.6
0.00	15.3	0.03	0.57	1.3	1.0	6.9	1.9	20.0	6.9	31.5	6.6	63.5	14.0
0.00	15.4	0.03	0.34	1.1	0.81	7.3	1.9	20.6	6.9	30.9	6.7	63.7	13.8
0±0	16.2±0.9	0.03±0.02	0.56±0.22	1.5±0.3	0.96±0.16	7.0±0.7	2.1±0.2	21.1±1.3	7.1±0.5	33.1±2.1	6.9±0.4	64.1±3.0	14.2±0.7
0±0	14.9±2.3	0.03±0.01	0.63±0.05	1.4±0.2	0.96±0.16	6.6±0.4	1.9±0.2	20.0±1.3	6.7±0.5	28.7±1.7	6.4±0.2	64.8±3.9	11.5±0.9

Table S6

Rayleigh distillation modeling results of internal Zr isotope zoning in single zircon grains.

Grain No.	$\delta^{94}Zr_{melt, 0}$ (‰) *	α †	Average	2SE	1000 ln(α)	2SE	T (°C) ‡	2SE	$10^6/T^2$ (K^{-2})	2SE
D17T107-3	0.05	0.99998	0.99988	0.00006	-0.12	0.06	831	9	0.820	0.013
D17T107-6	0.25	0.99995								
D17T107-8	0.25	0.99998								
D17T107-9	0.25	0.99980								
D17T107-12	0.25	0.99965								
D17T107-13	0.25	0.99990								
D17T107-14	0.05	0.99980								
D17T107-15	0.15	0.99990								
D17T107-16	0.25	0.99990								
D17T107-18	0.05	0.99985								
D17T107-19	0.05	0.99995								
D17T100-3	0.05	0.99980	0.99984	0.00007	-0.16	0.07	804	11	0.862	0.018
D17T100-11	0.05	0.99975								
D17T100-12	0.15	0.99985								
D17T100-13	0.05	0.99985								
D17T100-15	0.05	0.99995								
D17T102-1	0.05	0.99975	0.99974	0.00007	-0.26	0.07	743	16	0.968	0.031
D17T102-10	0.25	0.99965								
D17T102-24	0.05	0.99965								
D17T102-38	0.15	0.99990								
D17T102-41	0.25	0.99970								
D17T102-43	0.05	0.99980								
D17T102-44	0.25	0.99975								
D17T091-23	0.15	0.99965	0.99974	0.00012	-0.26	0.12	726	12	1.002	0.024
D17T091-24	0.15	0.99965								
D17T091-25	0.05	0.99975								
D17T091-26	0.05	0.99990								
D17T119-2	0.05	0.99975	0.99978	0.00007	-0.23	0.07	742	9	0.970	0.017
D17T119-5	0.05	0.99985								
D17T119-14	0.05	0.99975								
D17T119-15	0.15	0.99965								
D17T119-16	0.15	0.99990								
D17T119-17	0.15	0.99975								
D17T109-24	0.05	0.99955	0.99955	0.00004	-0.45	0.04	689	12	1.080	0.027
D7T109-25	0.05	0.99950								
D7T109-28	0.05	0.99960								
D7T109-32	0.15	0.99955								

* Assumed $\delta^{94}Zr$ values for the initial melt. † Zircon-melt fractionation factors estimated from the best-fit Rayleigh distillation models of Zr isotope profiles in single zircon grains. ‡ Median Ti-in-zircon temperatures.

Table S7

In-situ LA-MC-ICP-MS Zr isotope compositions of standard zircons and zircon megacryst Zr-Paki.

Analysis No.	Sample Name	Comment	$^{94}Zr/^{90}Zr$	2SE	$^{94}Zr/^{91}Zr$	2SE	$^{96}Zr/^{90}Zr$	2SE
GJ-1 as an external standard								
JUN06B02	GJ-1	Batch 1	0.358679	0.000014	1.62102	0.00007	0.059348	0.000004
JUN06B06	GJ-1	Batch 1	0.358692	0.000015	1.62109	0.00007	0.059354	0.000004
JUN06B10	GJ-1	Batch 1	0.358661	0.000018	1.62096	0.00009	0.059346	0.000004
JUN06B14	GJ-1	Batch 1	0.358661	0.000014	1.62091	0.00007	0.059339	0.000004
JUN06B18	GJ-1	Batch 1	0.358669	0.000013	1.62099	0.00007	0.059348	0.000004
JUN06B20	GJ-1	Batch 1	0.358607	0.000015	1.62077	0.00007	0.059331	0.000005
JUN06B24	GJ-1	Batch 1	0.358656	0.000015	1.62101	0.00007	0.059341	0.000004
JUN06B28	GJ-1	Batch 1	0.358655	0.000014	1.62095	0.00006	0.059346	0.000004
JUN06B32	GJ-1	Batch 1	0.358650	0.000016	1.62090	0.00007	0.059343	0.000004
JUN06B34	GJ-1	Batch 1	0.358644	0.000014	1.62083	0.00007	0.059343	0.000004
JUN06B38	GJ-1	Batch 1	0.358685	0.000014	1.62106	0.00008	0.059345	0.000003
JUN06B42	GJ-1	Batch 1	0.358675	0.000014	1.62100	0.00006	0.059346	0.000004
JUN06B46	GJ-1	Batch 1	0.358700	0.000014	1.62106	0.00006	0.059353	0.000004
JUN06B48	GJ-1	Batch 1	0.358695	0.000016	1.62107	0.00008	0.059347	0.000004
JUN06B52	GJ-1	Batch 1	0.358698	0.000015	1.62108	0.00006	0.059359	0.000005
JUN06B56	GJ-1	Batch 1	0.358673	0.000015	1.62094	0.00007	0.059350	0.000004
JUN06B60	GJ-1	Batch 1	0.358713	0.000015	1.62118	0.00006	0.059357	0.000005
JUN06B62	GJ-1	Batch 1	0.358664	0.000014	1.62096	0.00007	0.059348	0.000004
JUN06B66	GJ-1	Batch 1	0.358665	0.000014	1.62100	0.00007	0.059345	0.000004
JUN06B70	GJ-1	Batch 1	0.358664	0.000015	1.62100	0.00007	0.059343	0.000004
JUN06B74	GJ-1	Batch 1	0.358591	0.000013	1.62070	0.00006	0.059327	0.000003
JUN06B76	GJ-1	Batch 1	0.358589	0.000015	1.62067	0.00006	0.059332	0.000004
JUN06B80	GJ-1	Batch 1	0.358611	0.000011	1.62077	0.00005	0.059337	0.000003
JUN06B84	GJ-1	Batch 1	0.358621	0.000013	1.62076	0.00005	0.059335	0.000003
JUN06B87	GJ-1	Batch 1	0.358625	0.000017	1.62080	0.00007	0.059338	0.000004
JUN06B91	GJ-1	Batch 1	0.358621	0.000017	1.62080	0.00008	0.059336	0.000005
JUN06C01	GJ-1	Batch 1	0.358643	0.000014	1.62091	0.00007	0.059341	0.000003
JUN06C05	GJ-1	Batch 1	0.358634	0.000015	1.62085	0.00006	0.059339	0.000004
JUN06C09	GJ-1	Batch 1	0.358705	0.000014	1.62107	0.00006	0.059351	0.000005
JUN06C17	GJ-1	Batch 1	0.358702	0.000016	1.62107	0.00006	0.059355	0.000004
JUN06C19	GJ-1	Batch 1	0.358722	0.000012	1.62120	0.00006	0.059359	0.000004
JUN06C23	GJ-1	Batch 1	0.358701	0.000013	1.62111	0.00007	0.059356	0.000004
JUN06C27	GJ-1	Batch 1	0.358702	0.000015	1.62108	0.00006	0.059359	0.000004
JUN06C31	GJ-1	Batch 1	0.358697	0.000012	1.62108	0.00006	0.059354	0.000004
JUN06C33	GJ-1	Batch 1	0.358694	0.000016	1.62105	0.00007	0.059352	0.000004
JUN06C37	GJ-1	Batch 1	0.358722	0.000012	1.62113	0.00008	0.059357	0.000004
JUN06C41	GJ-1	Batch 1	0.358699	0.000016	1.62113	0.00007	0.059352	0.000004
JUN06C46	GJ-1	Batch 1	0.358692	0.000013	1.62105	0.00007	0.059354	0.000004
JUN06C50	GJ-1	Batch 1	0.358697	0.000017	1.62101	0.00007	0.059349	0.000004
JUN06C54	GJ-1	Batch 1	0.358695	0.000015	1.62104	0.00007	0.059350	0.000004
JUN06C58	GJ-1	Batch 1	0.358721	0.000011	1.62108	0.00007	0.059359	0.000003
JUN06C60	GJ-1	Batch 1	0.358688	0.000012	1.62107	0.00007	0.059353	0.000004
JUN06C64	GJ-1	Batch 1	0.358700	0.000016	1.62108	0.00008	0.059350	0.000004
JUN06C68	GJ-1	Batch 1	0.358718	0.000012	1.62115	0.00006	0.059358	0.000003
JUN06C72	GJ-1	Batch 1	0.358712	0.000015	1.62118	0.00007	0.059356	0.000004
JUN06C74	GJ-1	Batch 1	0.358711	0.000015	1.62109	0.00008	0.059355	0.000005
JUN06C78	GJ-1	Batch 1	0.358712	0.000016	1.62114	0.00008	0.059352	0.000004
JUN06C82	GJ-1	Batch 1	0.358698	0.000017	1.62106	0.00008	0.059350	0.000005
JUN06C86	GJ-1	Batch 1	0.358706	0.000016	1.62111	0.00008	0.059354	0.000004
JUN06C88	GJ-1	Batch 1	0.358719	0.000016	1.62111	0.00007	0.059359	0.000004
JUN06C91	GJ-1	Batch 1	0.358712	0.000019	1.62111	0.00008	0.059356	0.000004
JUN06C95	GJ-1	Batch 1	0.358728	0.000015	1.62117	0.00007	0.059365	0.000004
JUN06D01	GJ-1	Batch 1	0.358727	0.000017	1.62118	0.00008	0.059359	0.000005
JUN06D05	GJ-1	Batch 1	0.358717	0.000017	1.62116	0.00008	0.059358	0.000005
JUN06D09	GJ-1	Batch 1	0.358713	0.000017	1.62110	0.00008	0.059360	0.000004
JUN06D13	GJ-1	Batch 1	0.358708	0.000015	1.62110	0.00007	0.059358	0.000004
JUN06D17	GJ-1	Batch 1	0.358707	0.000014	1.62106	0.00008	0.059357	0.000004
JUN06D19	GJ-1	Batch 1	0.358722	0.000014	1.62116	0.00007	0.059356	0.000005
JUN06D23	GJ-1	Batch 1	0.358724	0.000018	1.62118	0.00010	0.059355	0.000004
JUN06D27	GJ-1	Batch 1	0.358702	0.000014	1.62111	0.00007	0.059365	0.000004
JUN06D31	GJ-1	Batch 1	0.358708	0.000019	1.62111	0.00008	0.059357	0.000005
JUN06D33	GJ-1	Batch 1	0.358718	0.000013	1.62116	0.00007	0.059356	0.000004
JUN06D37	GJ-1	Batch 1	0.358734	0.000012	1.62127	0.00006	0.059365	0.000003

^{90}Zr (V)	$\delta^{94/90}Zr_{GJ-1}$	2SE	$\delta^{94/91}Zr_{GJ-1}$	2SE	$\delta^{96/90}Zr_{GJ-1}$	2SE	$\delta^{94/90}Zr_{IPGP-Zr}$ †	2SE
9.5	-0.02	0.09	-0.02	0.09	-0.05	0.14	-0.03	0.10
9.6	0.04	0.10	0.04	0.10	0.06	0.14	0.03	0.11
9.8	0.00	0.10	0.01	0.11	0.06	0.15	-0.01	0.11
9.5	-0.01	0.08	-0.02	0.10	-0.07	0.15	-0.02	0.09
9.6	0.09	0.09	0.07	0.09	0.14	0.16	0.07	0.10
10.3	-0.07	0.09	-0.08	0.09	-0.08	0.16	-0.08	0.10
10.0	0.00	0.09	0.02	0.09	-0.04	0.14	-0.01	0.10
9.7	0.01	0.09	0.02	0.09	0.02	0.15	-0.01	0.10
10.0	0.01	0.09	0.02	0.10	0.01	0.15	0.00	0.10
9.9	-0.06	0.09	-0.07	0.10	-0.02	0.14	-0.07	0.10
9.5	0.01	0.09	0.02	0.10	-0.01	0.14	0.00	0.10
9.5	-0.04	0.09	-0.02	0.09	-0.06	0.16	-0.05	0.10
9.6	0.01	0.09	0.00	0.10	0.04	0.15	0.00	0.10
9.5	0.00	0.10	0.00	0.10	-0.10	0.17	-0.02	0.10
9.5	0.03	0.09	0.04	0.09	0.08	0.19	0.02	0.10
9.6	-0.06	0.09	-0.07	0.09	-0.06	0.17	-0.07	0.10
9.5	0.07	0.09	0.07	0.09	0.07	0.17	0.06	0.10
9.7	0.00	0.09	-0.01	0.09	0.03	0.15	-0.01	0.10
9.6	0.00	0.09	0.00	0.09	0.02	0.15	-0.01	0.10
9.8	0.10	0.09	0.09	0.09	0.14	0.14	0.09	0.10
10.7	0.00	0.09	0.01	0.09	-0.04	0.13	-0.01	0.10
10.7	-0.03	0.09	-0.03	0.08	-0.05	0.14	-0.04	0.10
10.6	-0.01	0.08	0.00	0.07	0.02	0.12	-0.03	0.09
10.7	0.00	0.13	0.00	0.11	0.00	0.21	-0.01	0.13
10.2	0.01	0.10	0.00	0.10	0.01	0.15	-0.01	0.11
10.2	-0.01	0.10	0.00	0.10	-0.01	0.16	-0.02	0.11
11.4	0.01	0.09	0.02	0.09	0.02	0.14	0.00	0.10
11.0	0.00	0.09	0.00	0.09	0.00	0.16	-0.01	0.10
9.5	0.00	0.09	0.00	0.09	-0.03	0.16	-0.01	0.10
9.6	-0.03	0.09	-0.04	0.09	-0.03	0.15	-0.04	0.10
9.4	0.03	0.08	0.03	0.09	0.02	0.16	0.02	0.09
9.3	0.00	0.08	0.01	0.09	-0.02	0.16	-0.01	0.09
9.2	0.01	0.09	0.00	0.09	0.04	0.16	0.00	0.10
9.2	0.00	0.08	0.01	0.09	0.02	0.16	-0.01	0.09
9.3	-0.04	0.09	-0.02	0.10	-0.04	0.16	-0.05	0.10
9.2	0.03	0.09	0.00	0.10	0.04	0.15	0.02	0.10
9.8	0.00	0.13	0.00	0.13	0.00	0.21	-0.01	0.14
9.4	-0.01	0.09	0.01	0.09	0.04	0.15	-0.02	0.10
9.2	0.00	0.10	-0.01	0.10	-0.01	0.16	-0.01	0.11
9.0	-0.04	0.08	-0.01	0.10	-0.07	0.14	-0.05	0.09
9.4	0.05	0.07	0.00	0.09	0.05	0.14	0.04	0.08
9.7	-0.02	0.09	0.00	0.10	0.03	0.16	-0.03	0.10
9.2	-0.03	0.09	-0.02	0.10	-0.06	0.15	-0.04	0.10
9.1	0.01	0.08	-0.01	0.09	0.02	0.13	0.00	0.09
9.4	0.00	0.09	0.03	0.10	0.01	0.16	-0.01	0.10
8.9	0.00	0.10	-0.01	0.11	0.03	0.17	-0.01	0.11
9.5	0.02	0.10	0.02	0.11	0.02	0.16	0.01	0.11
9.6	-0.01	0.10	-0.01	0.11	-0.04	0.18	-0.02	0.11
9.6	-0.02	0.10	0.00	0.11	-0.04	0.16	-0.03	0.11
9.6	0.01	0.11	0.00	0.10	0.02	0.15	0.00	0.12
9.3	-0.02	0.11	-0.02	0.10	-0.07	0.16	-0.03	0.12
9.8	0.02	0.10	0.02	0.10	0.07	0.16	0.01	0.11
9.3	0.01	0.11	0.00	0.11	0.01	0.18	0.00	0.12
9.3	0.01	0.11	0.02	0.11	-0.01	0.18	-0.01	0.12
9.7	0.01	0.10	0.00	0.11	0.02	0.17	-0.01	0.11
9.6	0.00	0.09	0.01	0.10	0.00	0.17	-0.01	0.10
9.3	-0.02	0.09	-0.03	0.10	0.01	0.17	-0.03	0.10
9.7	0.00	0.10	-0.01	0.11	0.01	0.16	-0.01	0.10
9.0	0.03	0.10	0.02	0.12	-0.08	0.15	0.02	0.11
8.9	-0.01	0.10	0.00	0.11	0.06	0.17	-0.02	0.11
9.4	-0.01	0.10	-0.01	0.11	0.01	0.17	-0.03	0.11
9.2	-0.02	0.08	-0.04	0.09	-0.07	0.13	-0.03	0.09
9.2	0.00	0.09	0.02	0.09	0.01	0.13	-0.01	0.10

Analysis No.	Sample Name	Comment	$^{94}Zr/^{90}Zr$	2SE	$^{94}Zr/^{91}Zr$	2SE	$^{96}Zr/^{90}Zr$	2SE
JUN06D41	GJ-1	Batch 1	0.358733	0.000016	1.62121	0.00007	0.059364	0.000003
JUN06D45	GJ-1	Batch 1	0.358736	0.000014	1.62127	0.00007	0.059362	0.000004
JUN06D47	GJ-1	Batch 1	0.358730	0.000016	1.62128	0.00008	0.059363	0.000005
JUN06D51	GJ-1	Batch 1	0.358701	0.000017	1.62112	0.00008	0.059359	0.000005
JUN06D55	GJ-1	Batch 1	0.358747	0.000019	1.62133	0.00008	0.059363	0.000005
JUN06D59	GJ-1	Batch 1	0.358685	0.000019	1.62107	0.00009	0.059353	0.000005
JUN06D61	GJ-1	Batch 1	0.358753	0.000016	1.62136	0.00008	0.059366	0.000004
JUN06D65	GJ-1	Batch 1	0.358759	0.000016	1.62127	0.00008	0.059368	0.000004
JUN06D69	GJ-1	Batch 1	0.358770	0.000015	1.62129	0.00008	0.059368	0.000004
JUN06D73	GJ-1	Batch 1	0.358753	0.000014	1.62121	0.00007	0.059367	0.000003
JUN06D75	GJ-1	Batch 1	0.358757	0.000016	1.62131	0.00007	0.059368	0.000004
JUN06D79	GJ-1	Batch 1	0.358767	0.000015	1.62131	0.00007	0.059373	0.000005
JUN06D83	GJ-1	Batch 1	0.358737	0.000015	1.62126	0.00007	0.059364	0.000004
JUN06D87	GJ-1	Batch 1	0.358787	0.000017	1.62139	0.00008	0.059365	0.000005
JUN06D91	GJ-1	Batch 1	0.358726	0.000012	1.62109	0.00005	0.059370	0.000004
JUN06D95	GJ-1	Batch 1	0.358774	0.000016	1.62131	0.00008	0.059377	0.000005
JUN06D99	GJ-1	Batch 1	0.358769	0.000015	1.62137	0.00009	0.059372	0.000004
JUL19A002	GJ-1	Batch 2	0.359884	0.000012	1.62514	0.00006	0.059660	0.000004
JUL19A003	GJ-1	Batch 2	0.359877	0.000012	1.62515	0.00006	0.059656	0.000003
JUL19A004	GJ-1	Batch 2	0.359876	0.000013	1.62514	0.00006	0.059653	0.000003
JUL19A007	GJ-1	Batch 2	0.359856	0.000009	1.62507	0.00006	0.059647	0.000004
JUL19A010	GJ-1	Batch 2	0.359873	0.000009	1.62520	0.00005	0.059652	0.000004
JUL19A013	GJ-1	Batch 2	0.359840	0.000012	1.62503	0.00006	0.059647	0.000004
JUL19A016	GJ-1	Batch 2	0.359853	0.000009	1.62506	0.00006	0.059648	0.000004
JUL19A019	GJ-1	Batch 2	0.359843	0.000011	1.62501	0.00006	0.059644	0.000003
JUL19A022	GJ-1	Batch 2	0.359817	0.000009	1.62493	0.00005	0.059642	0.000003
JUL19A025	GJ-1	Batch 2	0.359808	0.000019	1.62495	0.00008	0.059642	0.000005
JUL19A028	GJ-1	Batch 2	0.359834	0.000008	1.62498	0.00005	0.059641	0.000003
JUL19A031	GJ-1	Batch 2	0.359810	0.000012	1.62492	0.00007	0.059643	0.000004
JUL19A034	GJ-1	Batch 2	0.359819	0.000010	1.62494	0.00005	0.059641	0.000003
JUL19A037	GJ-1	Batch 2	0.359826	0.000014	1.62497	0.00006	0.059644	0.000004
JUL19A040	GJ-1	Batch 2	0.359818	0.000010	1.62494	0.00006	0.059641	0.000003
JUL19A043	GJ-1	Batch 2	0.359783	0.000010	1.62484	0.00005	0.059634	0.000003
JUL19A046	GJ-1	Batch 2	0.359816	0.000010	1.62496	0.00006	0.059642	0.000003
JUL19A049	GJ-1	Batch 2	0.359783	0.000009	1.62489	0.00005	0.059629	0.000003
JUL19A055	GJ-1	Batch 2	0.359778	0.000010	1.62483	0.00006	0.059631	0.000003
JUL19A058	GJ-1	Batch 2	0.359768	0.000008	1.62480	0.00004	0.059627	0.000003
JUL19A061	GJ-1	Batch 2	0.359773	0.000011	1.62480	0.00006	0.059630	0.000004
JUL19A064	GJ-1	Batch 2	0.359768	0.000012	1.62476	0.00005	0.059634	0.000003
JUL19A065	GJ-1	Batch 2	0.359769	0.000011	1.62481	0.00005	0.059628	0.000003
JUL19A068	GJ-1	Batch 2	0.359763	0.000010	1.62473	0.00006	0.059627	0.000003
JUL19A071	GJ-1	Batch 2	0.359740	0.000010	1.62468	0.00005	0.059622	0.000003
JUL19A074	GJ-1	Batch 2	0.359740	0.000009	1.62468	0.00004	0.059625	0.000003
JUL19A077	GJ-1	Batch 2	0.359748	0.000010	1.62472	0.00006	0.059624	0.000003
JUL19A080	GJ-1	Batch 2	0.359747	0.000009	1.62472	0.00004	0.059630	0.000003
JUL19A083	GJ-1	Batch 2	0.359761	0.000009	1.62474	0.00005	0.059631	0.000003
JUL19A086	GJ-1	Batch 2	0.359747	0.000011	1.62469	0.00005	0.059624	0.000003
JUL19A089	GJ-1	Batch 2	0.359744	0.000013	1.62476	0.00007	0.059626	0.000004
JUL19A092	GJ-1	Batch 2	0.359747	0.000010	1.62470	0.00006	0.059629	0.000003
JUL19A095	GJ-1	Batch 2	0.359725	0.000012	1.62469	0.00006	0.059618	0.000004
JUL19B001	GJ-1	Batch 2	0.359777	0.000011	1.62483	0.00005	0.059634	0.000003
JUL19B004	GJ-1	Batch 2	0.359744	0.000011	1.62469	0.00006	0.059623	0.000003
JUL19B007	GJ-1	Batch 2	0.359772	0.000014	1.62476	0.00006	0.059634	0.000003
JUL19B010	GJ-1	Batch 2	0.359737	0.000012	1.62467	0.00006	0.059622	0.000003
JUL19B013	GJ-1	Batch 2	0.359745	0.000010	1.62472	0.00005	0.059625	0.000003
JUL19B016	GJ-1	Batch 2	0.359738	0.000016	1.62467	0.00007	0.059618	0.000005
JUL19B019	GJ-1	Batch 2	0.359753	0.000010	1.62473	0.00006	0.059625	0.000003
JUL19B022	GJ-1	Batch 2	0.359733	0.000010	1.62469	0.00005	0.059622	0.000003
JUL19B025	GJ-1	Batch 2	0.359744	0.000011	1.62466	0.00006	0.059623	0.000004
JUL19B028	GJ-1	Batch 2	0.359741	0.000010	1.62469	0.00005	0.059626	0.000003
JUL19B031	GJ-1	Batch 2	0.359725	0.000016	1.62466	0.00007	0.059621	0.000004
JUL19B034	GJ-1	Batch 2	0.359749	0.000012	1.62468	0.00006	0.059625	0.000004
JUL19B037	GJ-1	Batch 2	0.359752	0.000010	1.62479	0.00006	0.059622	0.000003
JUL19B040	GJ-1	Batch 2	0.359746	0.000012	1.62473	0.00007	0.059623	0.000004
JUL19B043	GJ-1	Batch 2	0.359760	0.000012	1.62477	0.00005	0.059628	0.000004
JUL19B044	GJ-1	Batch 2	0.359750	0.000012	1.62474	0.00006	0.059623	0.000004
JUL19B047	GJ-1	Batch 2	0.359743	0.000010	1.62466	0.00005	0.059620	0.000003

^{90}Zr (V)	$\delta^{94/90}Zr_{GJ-1}$	2SE	$\delta^{94/91}Zr_{GJ-1}$	2SE	$\delta^{96/90}Zr_{GJ-1}$	2SE	$\delta^{94/90}Zr_{IPGP-Zr}$ †	2SE
8.8	0.00	0.10	-0.02	0.10	0.01	0.14	-0.02	0.10
8.7	0.01	0.09	0.00	0.10	-0.01	0.16	0.00	0.10
8.9	0.04	0.10	0.05	0.11	0.03	0.19	0.03	0.11
9.5	-0.06	0.11	-0.06	0.11	-0.03	0.19	-0.08	0.12
9.0	0.09	0.12	0.08	0.12	0.08	0.18	0.07	0.12
9.7	-0.09	0.11	-0.09	0.12	-0.11	0.18	-0.11	0.12
9.1	-0.01	0.10	0.03	0.11	-0.02	0.15	-0.02	0.11
9.1	-0.01	0.10	-0.01	0.11	0.01	0.15	-0.03	0.11
8.9	0.02	0.09	0.03	0.10	0.01	0.14	0.01	0.10
9.0	-0.01	0.09	-0.03	0.09	-0.01	0.14	-0.02	0.10
8.8	-0.01	0.10	0.00	0.10	-0.04	0.16	-0.03	0.11
9.3	0.04	0.09	0.02	0.10	0.08	0.17	0.03	0.10
9.2	-0.07	0.10	-0.04	0.10	-0.01	0.16	-0.08	0.11
8.8	0.09	0.10	0.09	0.10	-0.04	0.16	0.07	0.10
9.6	-0.07	0.09	-0.07	0.09	-0.06	0.16	-0.08	0.10
8.7	0.01	0.10	-0.02	0.12	0.05	0.17	-0.01	0.11
8.9	-0.01	0.10	0.02	0.12	-0.05	0.16	-0.02	0.11
10.7	0.01	0.08	0.00	0.08	0.05	0.14	0.00	0.09
11.0	0.00	0.08	0.00	0.08	-0.01	0.13	-0.02	0.09
11.3	0.03	0.07	0.02	0.09	0.05	0.13	0.02	0.09
10.2	-0.02	0.06	-0.04	0.08	-0.04	0.14	-0.04	0.07
9.4	0.05	0.06	0.05	0.08	0.04	0.15	0.03	0.08
11.3	-0.02	0.07	-0.01	0.08	-0.01	0.15	-0.03	0.08
10.9	0.01	0.06	0.02	0.08	0.03	0.13	0.00	0.07
11.8	0.04	0.06	0.02	0.08	0.02	0.12	0.02	0.08
12.0	0.01	0.09	0.00	0.08	0.00	0.15	0.00	0.10
12.6	-0.04	0.10	-0.01	0.09	0.00	0.16	-0.05	0.10
12.3	0.03	0.06	0.02	0.08	-0.01	0.13	0.02	0.07
11.6	-0.01	0.07	-0.01	0.08	0.01	0.12	-0.02	0.08
11.7	-0.01	0.07	-0.01	0.08	-0.03	0.12	-0.02	0.08
11.1	0.01	0.08	0.01	0.08	0.03	0.14	0.00	0.09
12.6	0.05	0.06	0.03	0.08	0.06	0.12	0.04	0.07
12.9	-0.05	0.06	-0.04	0.08	-0.07	0.12	-0.06	0.07
11.1	0.05	0.06	0.02	0.08	0.11	0.12	0.03	0.07
12.3	0.01	0.06	-0.02	0.07	-0.09	0.11	0.00	0.07
11.4	0.01	0.06	0.01	0.07	0.04	0.12	0.00	0.07
12.7	-0.01	0.06	0.00	0.07	-0.02	0.12	-0.02	0.07
11.6	0.01	0.07	0.01	0.08	-0.03	0.12	0.00	0.08
12.2	-0.01	0.07	-0.01	0.08	0.03	0.12	-0.02	0.08
13.1	0.01	0.07	0.02	0.08	0.01	0.13	0.00	0.08
12.7	0.03	0.06	0.01	0.08	0.04	0.12	0.02	0.08
13.3	0.00	0.06	0.00	0.07	-0.03	0.11	-0.01	0.07
13.3	-0.01	0.06	-0.01	0.07	0.01	0.11	-0.02	0.07
13.5	0.00	0.06	0.00	0.07	-0.05	0.11	-0.01	0.07
12.9	-0.02	0.06	-0.01	0.07	-0.01	0.12	-0.03	0.07
12.7	0.02	0.06	0.01	0.07	0.06	0.12	0.01	0.07
13.6	0.00	0.07	-0.02	0.08	-0.02	0.12	-0.01	0.08
13.4	0.00	0.07	0.02	0.08	-0.02	0.13	-0.02	0.09
13.1	0.03	0.07	0.00	0.08	0.09	0.13	0.02	0.08
13.6	-0.03	0.07	0.00	0.08	-0.09	0.14	-0.04	0.08
13.2	0.05	0.07	0.04	0.07	0.09	0.12	0.03	0.08
13.4	-0.04	0.07	-0.02	0.08	-0.09	0.12	-0.05	0.09
13.4	0.05	0.08	0.03	0.08	0.10	0.12	0.04	0.09
13.4	-0.01	0.07	-0.02	0.07	-0.03	0.12	-0.02	0.08
13.1	0.01	0.08	0.02	0.08	0.06	0.15	0.00	0.09
13.0	-0.02	0.09	-0.02	0.10	-0.06	0.15	-0.03	0.10
13.2	0.03	0.06	0.01	0.08	0.03	0.12	0.02	0.08
13.3	-0.02	0.06	0.01	0.07	0.00	0.14	-0.03	0.08
12.9	0.00	0.07	-0.01	0.08	-0.03	0.14	-0.01	0.08
13.0	0.02	0.08	0.01	0.08	0.05	0.14	0.01	0.09
13.3	-0.03	0.09	-0.01	0.09	-0.04	0.15	-0.05	0.10
13.2	0.00	0.07	-0.03	0.08	0.03	0.13	-0.02	0.08
12.2	0.01	0.07	0.02	0.08	-0.01	0.14	0.00	0.08
13.0	-0.02	0.08	-0.01	0.09	-0.05	0.15	-0.03	0.09
12.9	0.02	0.08	0.01	0.08	0.05	0.14	0.01	0.09
12.9	0.01	0.07	0.02	0.08	0.03	0.14	0.00	0.08
13.0	-0.02	0.06	-0.03	0.07	-0.07	0.12	-0.03	0.07

Analysis No.	Sample Name	Comment	$^{94}Zr/^{90}Zr$	2SE	$^{94}Zr/^{91}Zr$	2SE	$^{96}Zr/^{90}Zr$	2SE
JUL19B050	GJ-1	Batch 2	0.359754	0.000010	1.62476	0.00004	0.059628	0.000003
JUL19B053	GJ-1	Batch 2	0.359758	0.000013	1.62479	0.00007	0.059630	0.000004
JUL19B056	GJ-1	Batch 2	0.359761	0.000011	1.62479	0.00006	0.059629	0.000003
JUL19B059	GJ-1	Batch 2	0.359759	0.000010	1.62470	0.00007	0.059624	0.000003
JUL19B062	GJ-1	Batch 2	0.359749	0.000011	1.62471	0.00006	0.059625	0.000003
JUL19B065	GJ-1	Batch 2	0.359753	0.000010	1.62474	0.00005	0.059632	0.000003
JUL19B068	GJ-1	Batch 2	0.359753	0.000009	1.62475	0.00005	0.059627	0.000004
JUL19B071	GJ-1	Batch 2	0.359767	0.000012	1.62474	0.00006	0.059629	0.000004
JUL19B074	GJ-1	Batch 2	0.359757	0.000009	1.62470	0.00005	0.059628	0.000003
JUL19B077	GJ-1	Batch 2	0.359723	0.000011	1.62465	0.00005	0.059623	0.000004
JUL19B080	GJ-1	Batch 2	0.359737	0.000011	1.62472	0.00006	0.059627	0.000004
JUL19B083	GJ-1	Batch 2	0.359761	0.000013	1.62476	0.00006	0.059629	0.000004
JUL19B086	GJ-1	Batch 2	0.359723	0.000011	1.62462	0.00004	0.059623	0.000003
JUL19B089	GJ-1	Batch 2	0.359747	0.000011	1.62475	0.00007	0.059622	0.000003
JUL19B090	GJ-1	Batch 2	0.359767	0.000010	1.62470	0.00005	0.059632	0.000003
JUL19B093	GJ-1	Batch 2	0.359738	0.000012	1.62467	0.00005	0.059626	0.000003
JUL19C001	GJ-1	Batch 2	0.359770	0.000012	1.62477	0.00005	0.059633	0.000004
JUL19C004	GJ-1	Batch 2	0.359739	0.000011	1.62468	0.00005	0.059626	0.000004
JUL19C007	GJ-1	Batch 2	0.359716	0.000012	1.62457	0.00006	0.059618	0.000004
JUL19C010	GJ-1	Batch 2	0.359740	0.000013	1.62466	0.00006	0.059622	0.000004
JUL19C013	GJ-1	Batch 2	0.359711	0.000013	1.62457	0.00006	0.059619	0.000004
JUL19C016	GJ-1	Batch 2	0.359723	0.000012	1.62461	0.00005	0.059619	0.000004
JUL19C019	GJ-1	Batch 2	0.359720	0.000012	1.62458	0.00006	0.059617	0.000003
JUL19C022	GJ-1	Batch 2	0.359717	0.000009	1.62465	0.00006	0.059619	0.000003
JUL19C025	GJ-1	Batch 2	0.359687	0.000013	1.62456	0.00006	0.059614	0.000003
JUL19C026	GJ-1	Batch 2	0.359724	0.000011	1.62460	0.00005	0.059620	0.000003
JUL19C029	GJ-1	Batch 2	0.359737	0.000013	1.62468	0.00007	0.059622	0.000005
JUL19C032	GJ-1	Batch 2	0.359724	0.000009	1.62461	0.00005	0.059615	0.000003
JUL19C035	GJ-1	Batch 2	0.359739	0.000010	1.62468	0.00005	0.059621	0.000004
JUL19C038	GJ-1	Batch 2	0.359685	0.000012	1.62451	0.00005	0.059609	0.000003
JUL19C041	GJ-1	Batch 2	0.359693	0.000010	1.62457	0.00005	0.059614	0.000003
JUL19C044	GJ-1	Batch 2	0.359726	0.000014	1.62460	0.00006	0.059619	0.000004
JUL19C047	GJ-1	Batch 2	0.359724	0.000010	1.62465	0.00005	0.059621	0.000004
JUL19C050	GJ-1	Batch 2	0.359722	0.000012	1.62460	0.00005	0.059620	0.000003
JUL19C053	GJ-1	Batch 2	0.359696	0.000011	1.62452	0.00006	0.059612	0.000004
JUL19C056	GJ-1	Batch 2	0.359716	0.000011	1.62460	0.00006	0.059615	0.000003
JUL19C059	GJ-1	Batch 2	0.359740	0.000010	1.62468	0.00005	0.059621	0.000003
JUL19C062	GJ-1	Batch 2	0.359722	0.000011	1.62459	0.00005	0.059618	0.000003
JUL19C065	GJ-1	Batch 2	0.359748	0.000010	1.62466	0.00004	0.059622	0.000003
JUL19C068	GJ-1	Batch 2	0.359750	0.000013	1.62469	0.00006	0.059620	0.000004
JUL19C071	GJ-1	Batch 2	0.359716	0.000010	1.62455	0.00005	0.059617	0.000003
JUL19C074	GJ-1	Batch 2	0.359742	0.000012	1.62470	0.00006	0.059619	0.000004
JUL19C077	GJ-1	Batch 2	0.359699	0.000010	1.62455	0.00006	0.059616	0.000003
JUL19C080	GJ-1	Batch 2	0.359742	0.000010	1.62469	0.00005	0.059621	0.000003
JUL19C083	GJ-1	Batch 2	0.359753	0.000011	1.62472	0.00005	0.059627	0.000004
JUL19C086	GJ-1	Batch 2	0.359749	0.000014	1.62471	0.00006	0.059623	0.000003
JUL19C089	GJ-1	Batch 2	0.359732	0.000013	1.62468	0.00006	0.059619	0.000004
JUL19C092	GJ-1	Batch 2	0.359728	0.000012	1.62461	0.00006	0.059618	0.000003
JUL20A017	GJ-1	Batch 2	0.359930	0.000013	1.62533	0.00006	0.059674	0.000004
JUL20A020	GJ-1	Batch 2	0.359944	0.000015	1.62533	0.00007	0.059677	0.000005
JUL20A023	GJ-1	Batch 2	0.359995	0.000013	1.62554	0.00006	0.059689	0.000004
JUL20A026	GJ-1	Batch 2	0.360018	0.000010	1.62559	0.00006	0.059695	0.000003
JUL20A028	GJ-1	Batch 2	0.360052	0.000012	1.62568	0.00007	0.059704	0.000004
JUL20A029	GJ-1	Batch 2	0.360040	0.000011	1.62572	0.00006	0.059703	0.000004
JUL20A030	GJ-1	Batch 2	0.360046	0.000011	1.62573	0.00006	0.059703	0.000003
JUL20A033	GJ-1	Batch 2	0.360084	0.000010	1.62587	0.00005	0.059709	0.000004
JUL20A036	GJ-1	Batch 2	0.360098	0.000013	1.62592	0.00008	0.059709	0.000004
JUL20A039	GJ-1	Batch 2	0.360093	0.000014	1.62588	0.00006	0.059718	0.000004
JUL22A001	GJ-1	Batch 2	0.359606	0.000011	1.62418	0.00006	0.059590	0.000002
JUL22A002	GJ-1	Batch 2	0.359616	0.000014	1.62417	0.00006	0.059596	0.000004
JUL22A005	GJ-1	Batch 2	0.359628	0.000011	1.62425	0.00006	0.059600	0.000004
JUL22A008	GJ-1	Batch 2	0.359662	0.000014	1.62430	0.00006	0.059605	0.000003
JUL22A011	GJ-1	Batch 2	0.359666	0.000015	1.62441	0.00006	0.059604	0.000004
JUL22A014	GJ-1	Batch 2	0.359700	0.000012	1.62450	0.00006	0.059617	0.000003
JUL22A017	GJ-1	Batch 2	0.359729	0.000016	1.62457	0.00009	0.059625	0.000004
JUL22A020	GJ-1	Batch 2	0.359722	0.000014	1.62449	0.00007	0.059619	0.000004
JUL22A023	GJ-1	Batch 2	0.359713	0.000016	1.62448	0.00007	0.059617	0.000006

^{90}Zr (V)	$\delta^{94/90}Zr_{GJ-1}$	2SE	$\delta^{94/91}Zr_{GJ-1}$	2SE	$\delta^{96/90}Zr_{GJ-1}$	2SE	$\delta^{94/90}Zr_{IPGP-Zr}$ †	2SE
12.9	-0.01	0.07	-0.01	0.07	-0.01	0.13	-0.02	0.08
12.7	0.00	0.07	0.00	0.09	0.00	0.14	-0.02	0.09
12.9	0.00	0.07	0.03	0.09	0.05	0.13	-0.01	0.08
12.7	0.01	0.07	0.00	0.09	-0.01	0.12	0.00	0.08
12.8	-0.01	0.07	-0.01	0.08	-0.06	0.13	-0.02	0.08
13.0	0.00	0.06	0.00	0.08	0.04	0.12	-0.01	0.07
12.3	-0.02	0.07	0.00	0.08	-0.02	0.14	-0.03	0.08
12.6	0.01	0.07	0.01	0.08	0.01	0.13	0.00	0.08
13.3	0.05	0.06	0.02	0.07	0.04	0.13	0.04	0.08
13.4	-0.02	0.07	-0.02	0.08	-0.04	0.14	-0.03	0.08
13.3	-0.03	0.07	-0.01	0.08	-0.02	0.14	-0.05	0.09
13.7	0.05	0.08	0.04	0.08	0.05	0.13	0.04	0.09
13.6	-0.03	0.07	-0.04	0.07	0.01	0.11	-0.05	0.08
13.4	0.03	0.07	0.04	0.08	-0.01	0.11	0.02	0.08
13.9	0.04	0.07	0.01	0.07	0.06	0.13	0.03	0.08
13.5	-0.04	0.07	-0.01	0.07	-0.06	0.12	-0.05	0.08
13.6	0.04	0.07	0.03	0.07	0.05	0.13	0.03	0.08
13.2	0.03	0.07	0.03	0.08	0.06	0.14	0.02	0.08
13.3	-0.03	0.08	-0.03	0.08	-0.03	0.14	-0.05	0.09
13.8	0.04	0.08	0.03	0.08	0.03	0.14	0.03	0.09
13.7	-0.02	0.08	-0.01	0.08	0.00	0.13	-0.03	0.09
13.6	0.00	0.08	0.01	0.07	0.02	0.13	-0.01	0.09
13.5	0.00	0.07	-0.02	0.08	-0.02	0.12	-0.01	0.08
13.5	0.04	0.07	0.03	0.08	0.04	0.12	0.03	0.08
13.3	-0.04	0.07	-0.03	0.08	-0.04	0.12	-0.05	0.08
13.8	-0.02	0.07	-0.03	0.08	-0.02	0.15	-0.03	0.08
13.5	0.02	0.07	0.02	0.09	0.07	0.15	0.01	0.08
14.1	-0.02	0.06	-0.02	0.07	-0.06	0.12	-0.03	0.07
14.0	0.07	0.07	0.05	0.07	0.10	0.14	0.06	0.08
13.6	-0.01	0.07	-0.02	0.07	-0.04	0.12	-0.02	0.08
13.6	-0.05	0.07	-0.01	0.07	-0.04	0.12	-0.06	0.08
14.1	0.00	0.08	-0.02	0.08	-0.02	0.14	-0.01	0.09
13.5	0.00	0.07	0.01	0.07	0.01	0.13	-0.01	0.08
13.6	0.04	0.07	0.02	0.07	0.07	0.14	0.02	0.08
13.7	-0.03	0.07	-0.02	0.08	-0.03	0.14	-0.04	0.08
13.7	-0.03	0.07	-0.03	0.08	-0.05	0.13	-0.05	0.08
13.9	0.03	0.07	0.03	0.07	0.03	0.13	0.01	0.08
13.8	-0.04	0.07	-0.02	0.07	-0.03	0.12	-0.05	0.08
14.1	0.00	0.07	-0.01	0.07	0.01	0.13	-0.02	0.08
14.0	0.05	0.08	0.04	0.08	0.02	0.14	0.04	0.09
13.6	-0.04	0.07	-0.05	0.07	-0.02	0.13	-0.05	0.08
13.9	0.06	0.07	0.05	0.08	0.03	0.13	0.05	0.08
13.7	-0.06	0.06	-0.04	0.08	-0.04	0.12	-0.07	0.07
14.3	-0.02	0.06	-0.01	0.07	-0.05	0.13	-0.03	0.07
14.1	0.01	0.07	0.01	0.08	0.04	0.13	-0.01	0.09
14.3	0.02	0.08	0.01	0.08	0.03	0.13	0.01	0.09
14.1	0.01	0.08	0.02	0.08	0.01	0.14	-0.01	0.09
14.1	-0.01	0.08	-0.02	0.08	-0.01	0.14	-0.02	0.09
12.6	-0.02	0.09	0.00	0.09	-0.03	0.17	-0.03	0.10
12.5	-0.07	0.09	-0.06	0.09	-0.10	0.17	-0.08	0.10
12.4	-0.03	0.07	-0.01	0.08	-0.06	0.14	-0.04	0.08
12.6	-0.05	0.07	-0.03	0.08	-0.07	0.14	-0.06	0.08
12.8	0.02	0.06	-0.02	0.08	0.01	0.12	0.00	0.07
12.9	-0.02	0.06	0.01	0.08	-0.01	0.12	-0.03	0.07
12.8	-0.05	0.07	-0.04	0.08	-0.05	0.12	-0.06	0.08
12.7	-0.02	0.07	-0.02	0.09	0.00	0.13	-0.03	0.08
11.7	0.01	0.08	0.01	0.10	-0.08	0.14	0.00	0.09
11.6	-0.01	0.09	-0.01	0.10	0.08	0.14	-0.02	0.10
12.1	-0.03	0.07	-0.01	0.08	-0.09	0.12	-0.04	0.08
11.9	0.00	0.08	-0.02	0.08	0.01	0.13	-0.01	0.09
11.5	-0.05	0.08	-0.02	0.08	-0.04	0.13	-0.06	0.09
10.7	-0.01	0.09	-0.03	0.08	0.01	0.14	-0.02	0.10
11.0	-0.05	0.09	-0.03	0.08	-0.11	0.15	-0.06	0.10
10.7	-0.04	0.09	-0.02	0.10	-0.06	0.13	-0.05	0.10
10.3	0.01	0.10	0.02	0.11	0.04	0.15	0.00	0.10
10.6	0.01	0.05	0.00	0.04	0.02	0.07	0.00	0.06
11.1	0.03	0.09	0.01	0.09	0.04	0.20	0.02	0.10

Analysis No.	Sample Name	Comment	$^{94}Zr/^{90}Zr$	2SE	$^{94}Zr/^{91}Zr$	2SE	$^{96}Zr/^{90}Zr$	2SE
JUL22A026	GJ-1	Batch 2	0.359691	0.000013	1.62445	0.00006	0.059613	0.000004
JUL22A029	GJ-1	Batch 2	0.359693	0.000018	1.62442	0.00007	0.059614	0.000005
JUL22A032	GJ-1	Batch 2	0.359711	0.000013	1.62446	0.00006	0.059615	0.000004
JUL22A035	GJ-1	Batch 2	0.359745	0.000012	1.62461	0.00007	0.059623	0.000004
JUL22A038	GJ-1	Batch 2	0.359717	0.000010	1.62452	0.00005	0.059614	0.000004
JUL22A041	GJ-1	Batch 2	0.359759	0.000012	1.62468	0.00006	0.059631	0.000004
JUL22A044	GJ-1	Batch 2	0.359777	0.000015	1.62473	0.00007	0.059635	0.000004
JUL22A047	GJ-1	Batch 2	0.359798	0.000017	1.62477	0.00008	0.059640	0.000005
JUL22A050	GJ-1	Batch 2	0.359790	0.000012	1.62475	0.00007	0.059638	0.000004
JUL22A053	GJ-1	Batch 2	0.359781	0.000014	1.62475	0.00007	0.059635	0.000004
JUL22A056	GJ-1	Batch 2	0.359794	0.000012	1.62474	0.00006	0.059634	0.000004
JUL22A062	GJ-1	Batch 2	0.359798	0.000012	1.62478	0.00006	0.059637	0.000004
JUL22A065	GJ-1	Batch 2	0.359825	0.000014	1.62491	0.00007	0.059647	0.000004
JUL22A068	GJ-1	Batch 2	0.359809	0.000011	1.62486	0.00006	0.059645	0.000003
JUL22A073	GJ-1	Batch 2	0.359844	0.000012	1.62499	0.00008	0.059649	0.000003
JUL22A076	GJ-1	Batch 2	0.359862	0.000016	1.62506	0.00007	0.059651	0.000004
JUL22A082	GJ-1	Batch 2	0.359848	0.000015	1.62496	0.00007	0.059654	0.000004
JUL22A087	GJ-1	Batch 2	0.359849	0.000014	1.62498	0.00006	0.059655	0.000005
JUL22A090	GJ-1	Batch 2	0.359862	0.000019	1.62501	0.00009	0.059653	0.000005
JUL22B01	GJ-1	Batch 2	0.359725	0.000010	1.62456	0.00006	0.059623	0.000003
JUL22B02	GJ-1	Batch 2	0.359706	0.000010	1.62448	0.00005	0.059619	0.000003
JUL22B04	GJ-1	Batch 2	0.359687	0.000010	1.62444	0.00005	0.059613	0.000003
JUL22B07	GJ-1	Batch 2	0.359714	0.000013	1.62453	0.00007	0.059621	0.000005
JUL22B13	GJ-1	Batch 2	0.359721	0.000008	1.62454	0.00005	0.059602	0.000006
JUL22B16	GJ-1	Batch 2	0.359727	0.000008	1.62457	0.00005	0.059616	0.000003
JUL22B55	GJ-1	Batch 2	0.359455	0.000010	1.62363	0.00005	0.059554	0.000003
JUL22B56	GJ-1	Batch 2	0.359450	0.000007	1.62359	0.00005	0.059552	0.000003
JUL22B59	GJ-1	Batch 2	0.359441	0.000010	1.62357	0.00005	0.059551	0.000003
JUL22B62	GJ-1	Batch 2	0.359471	0.000010	1.62374	0.00005	0.059557	0.000002
JUL22B65	GJ-1	Batch 2	0.359413	0.000012	1.62346	0.00006	0.059543	0.000004
JUL22B68	GJ-1	Batch 2	0.359464	0.000009	1.62368	0.00005	0.059560	0.000003
JUL22B71	GJ-1	Batch 2	0.359468	0.000007	1.62371	0.00005	0.059557	0.000003
JUL22B74	GJ-1	Batch 2	0.359450	0.000013	1.62361	0.00006	0.059555	0.000004
JUL22B77	GJ-1	Batch 2	0.359407	0.000009	1.62351	0.00005	0.059541	0.000003
JUL22B80	GJ-1	Batch 2	0.359432	0.000017	1.62354	0.00007	0.059547	0.000004
JUL22B83	GJ-1	Batch 2	0.359414	0.000010	1.62354	0.00004	0.059543	0.000003
JUL22B86	GJ-1	Batch 2	0.359442	0.000011	1.62362	0.00005	0.059554	0.000004
JUL22B89	GJ-1	Batch 2	0.359433	0.000009	1.62356	0.00005	0.059550	0.000003
JUL22B93	GJ-1	Batch 2	0.359418	0.000011	1.62348	0.00006	0.059546	0.000003
JUL22B96	GJ-1	Batch 2	0.359386	0.000010	1.62340	0.00006	0.059539	0.000003
JUL22C01	GJ-1	Batch 2	0.359415	0.000010	1.62347	0.00004	0.059542	0.000003
JUL22C02	GJ-1	Batch 2	0.359406	0.000011	1.62351	0.00005	0.059542	0.000003
JUL22C05	GJ-1	Batch 2	0.359413	0.000012	1.62350	0.00005	0.059543	0.000003
JUL22C08	GJ-1	Batch 2	0.359364	0.000009	1.62338	0.00005	0.059529	0.000003
JUL22C11	GJ-1	Batch 2	0.359414	0.000008	1.62349	0.00005	0.059542	0.000003
JUL22C14	GJ-1	Batch 2	0.359413	0.000009	1.62349	0.00005	0.059544	0.000003
JUL22C17	GJ-1	Batch 2	0.359406	0.000009	1.62347	0.00005	0.059543	0.000003
JUL22C20	GJ-1	Batch 2	0.359386	0.000011	1.62341	0.00004	0.059538	0.000003
JUL22C24	GJ-1	Batch 2	0.359392	0.000010	1.62340	0.00005	0.059538	0.000004
JUL22C27	GJ-1	Batch 2	0.359361	0.000010	1.62332	0.00005	0.059530	0.000003
JUL22C30	GJ-1	Batch 2	0.359327	0.000009	1.62320	0.00005	0.059519	0.000003
JUL22C33	GJ-1	Batch 2	0.359369	0.000010	1.62335	0.00005	0.059535	0.000003
JUL22C36	GJ-1	Batch 2	0.359365	0.000010	1.62333	0.00005	0.059528	0.000003
JUL22C39	GJ-1	Batch 2	0.359349	0.000011	1.62329	0.00005	0.059526	0.000004
JUL22C42	GJ-1	Batch 2	0.359365	0.000009	1.62333	0.00006	0.059531	0.000003
JUL22C45	GJ-1	Batch 2	0.359366	0.000010	1.62333	0.00005	0.059532	0.000003
JUL22C48	GJ-1	Batch 2	0.359317	0.000011	1.62319	0.00005	0.059515	0.000003
JUL22C51	GJ-1	Batch 2	0.359343	0.000011	1.62324	0.00006	0.059525	0.000004
JUL22C54	GJ-1	Batch 2	0.359326	0.000010	1.62314	0.00005	0.059524	0.000003
JUL22C57	GJ-1	Batch 2	0.359319	0.000008	1.62319	0.00003	0.059519	0.000003
JUL22C60	GJ-1	Batch 2	0.359336	0.000010	1.62325	0.00005	0.059526	0.000004
JUL22C63	GJ-1	Batch 2	0.359359	0.000010	1.62331	0.00005	0.059528	0.000003
JUL22C67	GJ-1	Batch 2	0.359294	0.000010	1.62310	0.00005	0.059512	0.000003
JUL22C70	GJ-1	Batch 2	0.359332	0.000009	1.62324	0.00005	0.059520	0.000003
JUL22C71	GJ-1	Batch 2	0.359333	0.000011	1.62318	0.00006	0.059522	0.000003
JUL22C74	GJ-1	Batch 2	0.359341	0.000011	1.62329	0.00005	0.059524	0.000003
JUL22C77	GJ-1	Batch 2	0.359295	0.000009	1.62309	0.00005	0.059517	0.000003

^{90}Zr (V)	$\delta^{94/90}Zr_{GJ-1}$	2SE	$\delta^{94/91}Zr_{GJ-1}$	2SE	$\delta^{96/90}Zr_{GJ-1}$	2SE	$\delta^{94/90}Zr_{IPGP-Zr}$ †	2SE
11.3	0.00	0.09	0.01	0.09	-0.01	0.16	-0.01	0.10
11.0	-0.02	0.10	-0.01	0.10	-0.01	0.17	-0.04	0.11
11.1	-0.05	0.08	-0.05	0.09	-0.07	0.15	-0.06	0.09
10.9	0.04	0.07	0.03	0.08	0.08	0.15	0.03	0.08
11.1	-0.06	0.07	-0.05	0.08	-0.14	0.13	-0.07	0.08
10.6	-0.03	0.08	-0.01	0.09	-0.04	0.14	-0.04	0.09
10.1	-0.03	0.10	-0.01	0.10	-0.04	0.17	-0.04	0.11
10.3	0.01	0.10	0.01	0.11	0.01	0.18	0.00	0.11
10.2	0.01	0.08	0.00	0.09	0.03	0.14	0.00	0.09
10.9	-0.02	0.08	0.00	0.09	0.01	0.14	-0.03	0.09
11.1	-0.01	0.08	-0.01	0.09	-0.03	0.15	-0.02	0.09
10.8	-0.04	0.08	-0.04	0.09	-0.08	0.14	-0.05	0.09
10.4	0.02	0.08	0.01	0.09	0.02	0.13	0.01	0.09
10.2	-0.02	0.08	-0.01	0.09	-0.02	0.13	-0.03	0.09
10.4	-0.03	0.08	-0.02	0.10	-0.01	0.13	-0.04	0.09
10.1	0.02	0.10	0.02	0.09	-0.02	0.14	0.01	0.10
10.1	0.00	0.09	-0.04	0.08	-0.01	0.16	-0.01	0.10
11.0	-0.02	0.10	-0.01	0.10	0.01	0.18	-0.03	0.11
10.2	0.02	0.11	0.01	0.11	-0.01	0.19	0.01	0.11
13.9	0.05	0.06	0.04	0.07	0.08	0.12	0.04	0.08
14.2	0.00	0.06	-0.01	0.07	0.01	0.12	-0.01	0.08
14.4	-0.04	0.07	-0.03	0.08	-0.07	0.16	-0.05	0.08
14.2	-0.01	0.07	0.00	0.09	0.15	0.20	-0.02	0.08
14.3	-0.01	0.05	-0.01	0.08	-0.11	0.18	-0.02	0.07
14.0	0.00	0.05	0.00	0.08	0.00	0.11	-0.01	0.07
16.5	0.02	0.06	0.02	0.07	0.03	0.11	0.01	0.07
16.4	0.00	0.06	0.00	0.07	0.00	0.11	-0.01	0.07
16.8	-0.04	0.06	-0.05	0.06	-0.05	0.10	-0.05	0.07
16.4	0.08	0.07	0.09	0.07	0.11	0.12	0.07	0.08
16.7	-0.07	0.07	-0.07	0.08	-0.14	0.13	-0.08	0.08
16.2	-0.01	0.05	-0.01	0.07	0.03	0.11	-0.02	0.07
16.1	0.03	0.06	0.03	0.07	0.02	0.12	0.01	0.08
16.9	0.06	0.07	0.03	0.08	0.12	0.13	0.05	0.08
16.8	-0.03	0.08	-0.01	0.09	-0.05	0.13	-0.05	0.09
16.6	0.02	0.09	0.00	0.09	0.03	0.15	0.01	0.10
16.9	-0.04	0.07	-0.02	0.06	-0.09	0.13	-0.05	0.08
16.2	0.01	0.07	0.02	0.07	0.03	0.13	0.00	0.08
16.9	0.00	0.06	0.00	0.07	0.00	0.16	-0.01	0.08
17.0	0.04	0.07	0.03	0.08	0.06	0.12	0.03	0.08
17.1	-0.04	0.07	-0.03	0.08	-0.06	0.11	-0.06	0.08
17.1	0.01	0.07	-0.01	0.07	-0.01	0.12	0.00	0.08
17.1	-0.02	0.07	0.01	0.07	-0.01	0.12	-0.03	0.08
17.2	0.07	0.07	0.04	0.07	0.12	0.12	0.06	0.08
16.8	-0.07	0.06	-0.04	0.07	-0.11	0.11	-0.08	0.07
16.8	0.00	0.05	0.00	0.07	-0.02	0.10	-0.01	0.07
16.9	0.01	0.05	0.01	0.07	0.01	0.10	0.00	0.07
17.1	0.03	0.06	0.02	0.06	0.05	0.11	0.02	0.07
16.7	0.00	0.06	0.00	0.06	0.00	0.11	-0.01	0.08
16.9	0.04	0.06	0.03	0.07	0.07	0.13	0.03	0.07
17.1	0.05	0.06	0.04	0.07	0.09	0.12	0.03	0.07
17.1	-0.06	0.06	-0.05	0.07	-0.13	0.12	-0.07	0.07
17.1	0.00	0.06	0.01	0.07	0.06	0.12	-0.01	0.08
17.0	0.02	0.06	0.01	0.07	0.02	0.13	0.01	0.08
17.2	-0.02	0.06	-0.01	0.08	-0.05	0.13	-0.03	0.08
17.3	0.00	0.06	0.00	0.07	-0.01	0.13	-0.01	0.07
17.3	0.07	0.06	0.04	0.06	0.14	0.13	0.06	0.08
17.1	-0.04	0.07	-0.02	0.07	-0.09	0.13	-0.05	0.08
17.4	0.02	0.06	0.03	0.07	0.01	0.13	0.01	0.08
17.6	0.01	0.06	-0.02	0.06	0.04	0.12	0.00	0.07
17.3	-0.02	0.05	-0.02	0.06	-0.06	0.12	-0.04	0.07
17.3	-0.03	0.06	-0.02	0.07	-0.01	0.12	-0.04	0.07
17.1	0.00	0.06	0.00	0.07	0.00	0.11	-0.01	0.07
17.3	-0.05	0.06	-0.04	0.07	-0.06	0.11	-0.06	0.07
17.3	0.05	0.06	0.04	0.07	0.06	0.11	0.04	0.07
17.2	-0.01	0.07	-0.03	0.08	-0.02	0.12	-0.02	0.08
17.0	0.06	0.06	0.06	0.07	0.06	0.12	0.05	0.08
17.3	-0.02	0.06	0.00	0.06	0.02	0.12	-0.03	0.07

Analysis No.	Sample Name	Comment	$^{94}Zr/^{90}Zr$	2SE	$^{94}Zr/^{91}Zr$	2SE	$^{96}Zr/^{90}Zr$	2SE
JUL22C80	GJ-1	Batch 2	0.359307	0.000009	1.62311	0.00005	0.059515	0.000004
JUL22C83	GJ-1	Batch 2	0.359354	0.000009	1.62333	0.00005	0.059526	0.000003
JUL22C85	GJ-1	Batch 2	0.359305	0.000010	1.62317	0.00004	0.059513	0.000003
JUL22C88	GJ-1	Batch 2	0.359346	0.000009	1.62326	0.00005	0.059527	0.000003
JUL23A01	GJ-1	Batch 2	0.359337	0.000011	1.62323	0.00006	0.059522	0.000003
JUL23A04	GJ-1	Batch 2	0.359345	0.000010	1.62326	0.00005	0.059526	0.000003
JUL23A05	GJ-1	Batch 2	0.359324	0.000009	1.62322	0.00005	0.059522	0.000003
JUL23A06	GJ-1	Batch 2	0.359333	0.000010	1.62323	0.00005	0.059524	0.000003
JUL23A07	GJ-1	Batch 2	0.359303	0.000009	1.62312	0.00005	0.059515	0.000003
JUL23A10	GJ-1	Batch 2	0.359303	0.000007	1.62311	0.00004	0.059514	0.000003
JUL23A13	GJ-1	Batch 2	0.359297	0.000010	1.62306	0.00005	0.059511	0.000003
JUL23A16	GJ-1	Batch 2	0.359338	0.000010	1.62319	0.00005	0.059526	0.000003
JUL23A19	GJ-1	Batch 2	0.359342	0.000009	1.62329	0.00005	0.059526	0.000003
JUL23A22	GJ-1	Batch 2	0.359342	0.000011	1.62326	0.00005	0.059528	0.000003
JUL23A25	GJ-1	Batch 2	0.359318	0.000010	1.62318	0.00005	0.059516	0.000003
JUL23A31	GJ-1	Batch 2	0.359341	0.000010	1.62326	0.00006	0.059526	0.000003
JUL23A34	GJ-1	Batch 2	0.359304	0.000010	1.62311	0.00004	0.059518	0.000003
JUL23A37	GJ-1	Batch 2	0.359324	0.000011	1.62316	0.00005	0.059523	0.000003
JUL23A40	GJ-1	Batch 2	0.359348	0.000008	1.62325	0.00004	0.059525	0.000003
JUL23A44	GJ-1	Batch 2	0.359347	0.000010	1.62330	0.00006	0.059530	0.000003
JUL23A47	GJ-1	Batch 2	0.359310	0.000010	1.62314	0.00005	0.059516	0.000003
JUL23A50	GJ-1	Batch 2	0.359323	0.000008	1.62319	0.00004	0.059520	0.000003
JUL23A53	GJ-1	Batch 2	0.359323	0.000009	1.62321	0.00005	0.059517	0.000003
JUL23A56	GJ-1	Batch 2	0.359365	0.000011	1.62328	0.00005	0.059529	0.000003
JUL23A59	GJ-1	Batch 2	0.359382	0.000009	1.62335	0.00005	0.059531	0.000004
JUL23A62	GJ-1	Batch 2	0.359360	0.000010	1.62331	0.00006	0.059526	0.000003
JUL23A65	GJ-1	Batch 2	0.359382	0.000009	1.62340	0.00005	0.059538	0.000003
JUL23A69	GJ-1	Batch 2	0.359329	0.000011	1.62318	0.00005	0.059522	0.000004
JUL23A72	GJ-1	Batch 2	0.359261	0.000011	1.62299	0.00006	0.059504	0.000004
JUL23A75	GJ-1	Batch 2	0.359307	0.000009	1.62310	0.00005	0.059514	0.000003
JUL23A78	GJ-1	Batch 2	0.359279	0.000009	1.62302	0.00005	0.059508	0.000003
JUL23A82	GJ-1	Batch 2	0.359280	0.000013	1.62304	0.00006	0.059513	0.000003
JUL23A85	GJ-1	Batch 2	0.359312	0.000011	1.62314	0.00005	0.059512	0.000003
JUL23B13	GJ-1	Batch 2	0.359439	0.000009	1.62361	0.00005	0.059549	0.000003
JUL23B16	GJ-1	Batch 2	0.359438	0.000010	1.62356	0.00005	0.059547	0.000003
JUL23B19	GJ-1	Batch 2	0.359351	0.000010	1.62326	0.00005	0.059528	0.000003
JUL23B22	GJ-1	Batch 2	0.359397	0.000012	1.62342	0.00005	0.059541	0.000003
JUL23B25	GJ-1	Batch 2	0.359435	0.000012	1.62357	0.00006	0.059550	0.000003
JUL23B26	GJ-1	Batch 2	0.359474	0.000009	1.62367	0.00005	0.059560	0.000003
JUL23B29	GJ-1	Batch 2	0.359495	0.000010	1.62377	0.00005	0.059568	0.000003
JUL23B32	GJ-1	Batch 2	0.359492	0.000011	1.62374	0.00005	0.059562	0.000004
JUL23B35	GJ-1	Batch 2	0.359433	0.000013	1.62355	0.00005	0.059547	0.000004
JUL23B38	GJ-1	Batch 2	0.359441	0.000010	1.62355	0.00006	0.059551	0.000003
JUL23B41	GJ-1	Batch 2	0.359440	0.000010	1.62358	0.00005	0.059548	0.000003
JUL23B47	GJ-1	Batch 2	0.359472	0.000009	1.62366	0.00005	0.059558	0.000003
JUL23B50	GJ-1	Batch 2	0.359497	0.000009	1.62375	0.00005	0.059563	0.000003
JUL23B53	GJ-1	Batch 2	0.359483	0.000011	1.62374	0.00005	0.059563	0.000003
JUL23B56	GJ-1	Batch 2	0.359467	0.000010	1.62368	0.00005	0.059558	0.000003
JUL23B59	GJ-1	Batch 2	0.359495	0.000009	1.62374	0.00005	0.059563	0.000003
JUL23B62	GJ-1	Batch 2	0.359465	0.000012	1.62364	0.00006	0.059558	0.000003
JUL23B65	GJ-1	Batch 2	0.359495	0.000012	1.62378	0.00005	0.059561	0.000004
JUL23B68	GJ-1	Batch 2	0.359467	0.000011	1.62365	0.00005	0.059557	0.000003
JUL23B71	GJ-1	Batch 2	0.359467	0.000012	1.62368	0.00005	0.059557	0.000003
JUL23B74	GJ-1	Batch 2	0.359504	0.000010	1.62376	0.00005	0.059566	0.000003
JUL23B77	GJ-1	Batch 2	0.359515	0.000012	1.62382	0.00006	0.059567	0.000004
Average (±2SD)								
Ref. (16) *								
GJ-1 as an unknown								
MAY31B21	GJ-1	Batch 1	0.358445	0.000015	1.62020	0.00007	0.059301	0.000005
MAY31B29	GJ-1	Batch 1	0.358429	0.000014	1.62022	0.00006	0.059293	0.000005
MAY31B45	GJ-1	Batch 1	0.358406	0.000012	1.62013	0.00006	0.059288	0.000004
MAY31B49	GJ-1	Batch 1	0.358429	0.000013	1.62015	0.00007	0.059294	0.000004
MAY31B58	GJ-1	Batch 1	0.358401	0.000012	1.62016	0.00007	0.059287	0.000004
MAY31B61	GJ-1	Batch 1	0.358413	0.000014	1.62009	0.00007	0.059291	0.000005
MAY31B73	GJ-1	Batch 1	0.358436	0.000011	1.62023	0.00006	0.059291	0.000003

^{90}Zr (V)	$\delta^{94/90}Zr_{GJ-1}$	2SE	$\delta^{94/91}Zr_{GJ-1}$	2SE	$\delta^{96/90}Zr_{GJ-1}$	2SE	$\delta^{94/90}Zr_{IPGP-Zr}$ †	2SE
17.3	-0.07	0.06	-0.07	0.07	-0.10	0.13	-0.08	0.07
17.0	0.07	0.06	0.05	0.07	0.11	0.11	0.06	0.07
17.5	-0.06	0.06	-0.03	0.07	-0.12	0.11	-0.07	0.07
17.0	0.06	0.06	0.03	0.07	0.12	0.11	0.05	0.07
17.2	-0.01	0.07	-0.01	0.07	-0.03	0.12	-0.02	0.08
17.4	0.05	0.05	0.03	0.05	0.07	0.09	0.04	0.06
17.3	-0.01	0.05	0.01	0.05	0.01	0.09	-0.02	0.06
17.2	0.02	0.05	0.01	0.05	0.03	0.09	0.01	0.06
17.4	0.00	0.05	0.00	0.06	0.01	0.11	-0.01	0.07
17.4	0.01	0.05	0.02	0.06	0.03	0.11	0.00	0.07
17.3	-0.06	0.06	-0.04	0.07	-0.12	0.12	-0.07	0.07
17.1	-0.01	0.06	-0.03	0.07	-0.01	0.12	-0.02	0.07
17.2	0.00	0.06	0.01	0.07	-0.01	0.11	-0.01	0.07
17.2	0.03	0.06	0.03	0.07	0.10	0.11	0.02	0.08
17.2	0.00	0.06	0.00	0.08	0.00	0.12	-0.01	0.08
17.2	0.05	0.06	0.05	0.07	0.06	0.12	0.04	0.07
17.3	-0.03	0.06	-0.01	0.06	-0.04	0.11	-0.04	0.08
17.2	-0.03	0.06	-0.03	0.07	-0.02	0.11	-0.05	0.07
17.3	0.00	0.06	0.00	0.10	-0.04	0.10	-0.01	0.07
17.5	0.05	0.06	0.05	0.07	0.11	0.12	0.04	0.08
16.9	-0.02	0.06	-0.02	0.07	-0.03	0.11	-0.03	0.07
17.2	0.00	0.05	0.00	0.06	0.02	0.11	-0.01	0.07
17.3	-0.06	0.06	-0.02	0.07	-0.10	0.11	-0.07	0.07
17.0	-0.02	0.06	-0.02	0.07	-0.02	0.12	-0.04	0.07
17.0	0.03	0.06	0.01	0.07	0.04	0.12	0.02	0.07
17.0	-0.03	0.06	-0.03	0.07	-0.10	0.11	-0.04	0.07
17.2	0.07	0.06	0.00	0.09	0.13	0.12	0.06	0.07
17.3	0.09	0.07	0.06	0.08	0.15	0.14	0.08	0.08
17.0	-0.06	0.06	-0.03	0.08	-0.08	0.14	-0.08	0.08
17.3	0.04	0.06	0.03	0.07	0.05	0.10	0.03	0.07
17.4	0.00	0.07	0.00	0.09	-0.05	0.12	-0.01	0.08
17.5	-0.04	0.07	-0.03	0.08	0.01	0.12	-0.06	0.09
17.5	0.04	0.07	0.03	0.07	-0.01	0.12	0.03	0.08
16.2	0.00	0.06	0.02	0.07	0.02	0.11	-0.01	0.07
16.6	0.12	0.06	0.09	0.07	0.16	0.11	0.11	0.08
17.4	-0.06	0.07	-0.05	0.07	-0.11	0.12	-0.08	0.08
17.1	-0.05	0.07	-0.05	0.07	-0.08	0.12	-0.06	0.08
16.4	0.05	0.07	0.05	0.07	0.08	0.12	0.04	0.09
16.7	-0.03	0.06	-0.03	0.07	-0.07	0.12	-0.04	0.07
16.8	0.01	0.07	0.01	0.07	0.05	0.12	-0.01	0.08
16.8	0.08	0.08	0.06	0.07	0.13	0.14	0.07	0.09
16.3	-0.01	0.07	0.00	0.08	-0.04	0.14	-0.02	0.09
15.8	0.00	0.06	-0.01	0.08	0.03	0.11	-0.01	0.08
15.5	0.00	0.06	0.00	0.06	0.00	0.12	-0.01	0.07
16.4	-0.03	0.05	-0.03	0.06	-0.05	0.13	-0.05	0.07
16.3	0.02	0.06	0.00	0.07	0.01	0.12	0.01	0.07
15.8	0.02	0.06	0.02	0.07	0.04	0.12	0.01	0.08
15.6	-0.04	0.06	-0.02	0.07	-0.05	0.12	-0.05	0.07
15.9	0.04	0.06	0.03	0.07	0.04	0.12	0.03	0.07
15.8	-0.04	0.07	-0.04	0.07	-0.02	0.13	-0.05	0.08
16.7	0.04	0.07	0.04	0.07	0.03	0.13	0.03	0.08
16.3	0.00	0.07	-0.01	0.07	0.00	0.11	-0.01	0.08
16.2	-0.05	0.07	-0.03	0.07	-0.08	0.12	-0.06	0.08
16.6	-0.01	0.07	-0.02	0.07	-0.01	0.12	-0.03	0.08
16.5	0.01	0.07	0.02	0.07	0.01	0.13	0.00	0.08
	0±0.07		0±0.06		0±0.11		-0.01±0.07	
	-		-		-		-0.012±0.042	
9.3	0.12	0.10	0.06	0.10	0.17	0.14	0.10	0.11
9.0	0.07	0.11	0.07	0.09	0.03	0.16	0.06	0.11
9.1	-0.01	0.11	-0.03	0.10	-0.04	0.15	-0.02	0.11
8.9	0.07	0.10	0.01	0.10	0.07	0.15	0.05	0.11
9.1	0.04	0.10	0.05	0.09	0.05	0.18	0.03	0.11
9.2	0.08	0.10	0.03	0.09	0.08	0.15	0.06	0.11
9.0	0.09	0.09	0.07	0.09	0.03	0.15	0.08	0.10

Analysis No.	Sample Name	Comment	$^{94}Zr/^{90}Zr$	2SE	$^{94}Zr/^{91}Zr$	2SE	$^{96}Zr/^{90}Zr$	2SE
MAY31B85	GJ-1	Batch 1	0.358426	0.000010	1.62018	0.00006	0.059294	0.000004
MAY31B92	GJ-1	Batch 1	0.358455	0.000011	1.62028	0.00008	0.059296	0.000004
MAY31B94	GJ-1	Batch 1	0.358467	0.000012	1.62035	0.00006	0.059303	0.000004
MAY31C06	GJ-1	Batch 1	0.358451	0.000011	1.62023	0.00006	0.059297	0.000004
MAY31C17	GJ-1	Batch 1	0.358465	0.000012	1.62032	0.00006	0.059304	0.000004
MAY31C29	GJ-1	Batch 1	0.358489	0.000013	1.62037	0.00006	0.059309	0.000004
MAY31D02	GJ-1	Batch 1	0.358458	0.000010	1.62032	0.00006	0.059298	0.000004
MAY31D11	GJ-1	Batch 1	0.358490	0.000011	1.62038	0.00006	0.059301	0.000004
MAY31D17	GJ-1	Batch 1	0.358469	0.000012	1.62035	0.00006	0.059305	0.000004
MAY31D25	GJ-1	Batch 1	0.358491	0.000011	1.62036	0.00005	0.059313	0.000004
MAY31D34	GJ-1	Batch 1	0.358514	0.000013	1.62048	0.00007	0.059314	0.000005
MAY31D44	GJ-1	Batch 1	0.358531	0.000010	1.62055	0.00007	0.059319	0.000004
MAY31E25	GJ-1	Batch 1	0.358740	0.000011	1.62120	0.00006	0.059368	0.000005
MAY31E37	GJ-1	Batch 1	0.358758	0.000013	1.62132	0.00006	0.059375	0.000004
MAY31E49	GJ-1	Batch 1	0.358768	0.000012	1.62131	0.00006	0.059376	0.000004
MAY31E58	GJ-1	Batch 1	0.358812	0.000012	1.62146	0.00007	0.059388	0.000004
MAY31E61	GJ-1	Batch 1	0.358784	0.000013	1.62141	0.00006	0.059384	0.000004
MAY31F03	GJ-1	Batch 1	0.358703	0.000011	1.62117	0.00005	0.059359	0.000005
MAY31F13	GJ-1	Batch 1	0.358714	0.000011	1.62114	0.00005	0.059363	0.000003
MAY31F25	GJ-1	Batch 1	0.358731	0.000011	1.62121	0.00007	0.059369	0.000004
MAY31F37	GJ-1	Batch 1	0.358720	0.000011	1.62117	0.00005	0.059365	0.000004
MAY31F48	GJ-1	Batch 1	0.358731	0.000013	1.62120	0.00006	0.059368	0.000004
JUN06A02	GJ-1	Batch 1	0.358605	0.000016	1.62073	0.00006	0.059327	0.000005
JUN06A03	GJ-1	Batch 1	0.358578	0.000016	1.62067	0.00008	0.059324	0.000005
JUN06A08	GJ-1	Batch 1	0.358599	0.000018	1.62077	0.00008	0.059330	0.000005
JUN06A09	GJ-1	Batch 1	0.358588	0.000019	1.62072	0.00008	0.059319	0.000005
JUN06A24	GJ-1	Batch 1	0.358607	0.000017	1.62077	0.00008	0.059329	0.000005
JUN06A38	GJ-1	Batch 1	0.358635	0.000022	1.62087	0.00009	0.059336	0.000006
JUN06A54	GJ-1	Batch 1	0.358574	0.000017	1.62068	0.00007	0.059321	0.000005
JUN06A68	GJ-1	Batch 1	0.358588	0.000015	1.62067	0.00007	0.059323	0.000005
JUN06A82	GJ-1	Batch 1	0.358665	0.000019	1.62090	0.00008	0.059340	0.000005
JUN06A96	GJ-1	Batch 1	0.358684	0.000019	1.62101	0.00009	0.059353	0.000004
Average (±2SD)								
Ref. (16) [*]								
Penglai as an external standard								
MAY31B11	Penglai	Batch 1	0.358374	0.000013	1.62002	0.00006	0.059278	0.000004
MAY31B14	Penglai	Batch 1	0.358386	0.000013	1.62005	0.00006	0.059284	0.000004
MAY31B17	Penglai	Batch 1	0.358355	0.000013	1.62001	0.00006	0.059274	0.000005
MAY31B20	Penglai	Batch 1	0.358350	0.000012	1.61991	0.00007	0.059274	0.000004
MAY31B23	Penglai	Batch 1	0.358380	0.000012	1.61998	0.00007	0.059281	0.000003
MAY31B26	Penglai	Batch 1	0.358366	0.000012	1.61995	0.00006	0.059278	0.000004
MAY31B31	Penglai	Batch 1	0.358362	0.000016	1.61993	0.00008	0.059278	0.000004
MAY31B44	Penglai	Batch 1	0.358373	0.000015	1.62006	0.00007	0.059275	0.000004
MAY31B47	Penglai	Batch 1	0.358367	0.000014	1.61998	0.00007	0.059279	0.000004
MAY31B50	Penglai	Batch 1	0.358367	0.000011	1.61997	0.00007	0.059274	0.000004
MAY31B53	Penglai	Batch 1	0.358303	0.000013	1.61978	0.00007	0.059264	0.000003
MAY31B56	Penglai	Batch 1	0.358364	0.000013	1.62000	0.00005	0.059270	0.000005
MAY31B59	Penglai	Batch 1	0.358335	0.000015	1.61984	0.00007	0.059271	0.000005
MAY31B60	Penglai	Batch 1	0.358330	0.000011	1.61982	0.00006	0.059271	0.000004
MAY31B63	Penglai	Batch 1	0.358365	0.000012	1.61996	0.00007	0.059275	0.000004
MAY31B66	Penglai	Batch 1	0.358355	0.000013	1.61990	0.00007	0.059273	0.000003
MAY31B69	Penglai	Batch 1	0.358362	0.000013	1.61999	0.00006	0.059275	0.000004
MAY31B72	Penglai	Batch 1	0.358354	0.000013	1.61991	0.00006	0.059273	0.000003
MAY31B75	Penglai	Batch 1	0.358378	0.000012	1.62001	0.00006	0.059279	0.000004
MAY31B78	Penglai	Batch 1	0.358331	0.000012	1.61988	0.00006	0.059263	0.000004
MAY31B81	Penglai	Batch 1	0.358383	0.000011	1.62002	0.00007	0.059282	0.000004
MAY31B84	Penglai	Batch 1	0.358358	0.000013	1.61992	0.00006	0.059278	0.000004
MAY31B87	Penglai	Batch 1	0.358388	0.000013	1.62000	0.00006	0.059279	0.000004
MAY31B90	Penglai	Batch 1	0.358393	0.000012	1.62008	0.00006	0.059282	0.000003
MAY31B93	Penglai	Batch 1	0.358402	0.000012	1.62006	0.00008	0.059286	0.000004
MAY31B96	Penglai	Batch 1	0.358369	0.000012	1.61996	0.00005	0.059279	0.000005
MAY31C04	Penglai	Batch 1	0.358393	0.000012	1.62047	0.00011	0.059282	0.000004
MAY31C05	Penglai	Batch 1	0.358376	0.000011	1.62003	0.00006	0.059278	0.000004
MAY31C07	Penglai	Batch 1	0.358384	0.000013	1.62003	0.00006	0.059276	0.000004
MAY31C10	Penglai	Batch 1	0.358383	0.000013	1.62009	0.00006	0.059278	0.000004

^{90}Zr (V)	$\delta^{94/90}Zr_{GJ-1}$	2SE	$\delta^{94/91}Zr_{GJ-1}$	2SE	$\delta^{96/90}Zr_{GJ-1}$	2SE	$\delta^{94/90}Zr_{IPGP-Zr}$ †	2SE
9.2	0.04	0.10	0.03	0.08	0.03	0.16	0.03	0.11
8.6	0.05	0.10	0.03	0.10	-0.01	0.14	0.04	0.10
9.5	0.12	0.10	0.11	0.09	0.13	0.18	0.11	0.10
9.1	0.08	0.09	-0.07	0.10	0.10	0.16	0.07	0.10
9.2	0.03	0.09	-0.03	0.09	0.10	0.16	0.02	0.10
8.8	0.21	0.09	0.14	0.09	0.25	0.17	0.20	0.10
9.1	0.12	0.09	0.08	0.09	0.11	0.14	0.11	0.10
9.0	0.18	0.10	0.08	0.09	0.13	0.16	0.17	0.11
9.4	0.12	0.09	0.06	0.09	0.19	0.16	0.11	0.10
8.7	0.11	0.10	0.06	0.09	0.21	0.16	0.10	0.11
8.9	0.11	0.09	0.04	0.09	0.11	0.15	0.10	0.10
8.7	0.04	0.10	0.00	0.09	0.02	0.15	0.03	0.11
8.3	0.08	0.09	0.02	0.08	-0.02	0.16	0.06	0.10
8.4	0.15	0.10	0.10	0.08	0.22	0.16	0.14	0.11
8.6	0.04	0.09	-0.01	0.08	-0.05	0.15	0.03	0.10
8.4	0.14	0.10	0.06	0.08	0.11	0.16	0.13	0.10
8.7	0.05	0.10	0.03	0.08	0.06	0.16	0.04	0.11
8.6	0.03	0.10	0.03	0.09	-0.03	0.17	0.02	0.11
8.4	0.04	0.09	0.02	0.09	0.03	0.15	0.03	0.10
8.5	0.13	0.10	0.07	0.09	0.17	0.16	0.11	0.11
8.3	0.03	0.09	-0.01	0.09	0.00	0.17	0.02	0.10
8.2	0.10	0.10	0.04	0.09	0.13	0.16	0.09	0.11
8.9	0.09	0.10	0.02	0.09	0.01	0.15	0.08	0.11
9.3	0.02	0.10	-0.02	0.09	-0.04	0.15	0.00	0.11
8.7	0.16	0.11	0.13	0.09	0.17	0.18	0.14	0.12
8.6	0.13	0.11	0.10	0.09	-0.03	0.18	0.11	0.12
9.1	0.03	0.10	0.02	0.09	-0.09	0.15	0.02	0.11
8.8	0.20	0.14	0.15	0.12	0.14	0.22	0.19	0.15
8.7	0.03	0.12	0.05	0.10	-0.05	0.18	0.02	0.13
8.6	0.01	0.12	-0.06	0.10	-0.10	0.18	0.00	0.12
8.7	-0.02	0.11	-0.07	0.10	-0.25	0.18	-0.03	0.12
8.6	0.04	0.11	0.02	0.10	0.02	0.15	0.03	0.12
	0.08±0.11		0.04±0.11		0.05±0.2		0.07±0.11	
	-		-		-		-0.012±0.042	
10.3	-0.12	0.10	-0.11	0.09	-0.27	0.16	-0.14	0.11
10.3	-0.06	0.10	-0.09	0.09	-0.14	0.17	-0.08	0.11
10.8	-0.10	0.10	-0.07	0.09	-0.22	0.17	-0.11	0.11
10.8	-0.15	0.09	-0.12	0.10	-0.28	0.14	-0.16	0.10
10.5	-0.09	0.09	-0.09	0.09	-0.20	0.13	-0.10	0.10
10.4	-0.11	0.10	-0.10	0.09	-0.22	0.15	-0.12	0.11
10.0	-0.12	0.11	-0.11	0.10	-0.23	0.15	-0.13	0.11
9.9	-0.10	0.11	-0.07	0.10	-0.26	0.16	-0.11	0.12
9.6	-0.11	0.10	-0.10	0.10	-0.18	0.15	-0.12	0.11
10.2	-0.02	0.09	-0.04	0.09	-0.14	0.15	-0.03	0.10
11.2	-0.19	0.10	-0.17	0.09	-0.27	0.15	-0.20	0.11
10.3	-0.07	0.10	-0.05	0.08	-0.22	0.19	-0.08	0.11
10.1	-0.15	0.11	-0.15	0.09	-0.22	0.19	-0.16	0.12
11.4	-0.16	0.09	-0.14	0.09	-0.26	0.15	-0.17	0.10
10.9	-0.09	0.10	-0.08	0.10	-0.21	0.14	-0.10	0.11
10.5	-0.12	0.10	-0.13	0.09	-0.23	0.14	-0.13	0.11
9.9	-0.10	0.10	-0.07	0.08	-0.20	0.15	-0.11	0.11
10.6	-0.14	0.10	-0.13	0.08	-0.27	0.15	-0.15	0.11
10.2	-0.04	0.09	-0.06	0.09	-0.09	0.16	-0.05	0.10
11.3	-0.18	0.09	-0.14	0.09	-0.38	0.14	-0.19	0.10
10.3	-0.07	0.10	-0.07	0.09	-0.18	0.16	-0.08	0.10
10.6	-0.15	0.10	-0.13	0.09	-0.23	0.16	-0.16	0.11
10.3	-0.11	0.10	-0.12	0.08	-0.24	0.15	-0.13	0.11
10.4	-0.12	0.10	-0.09	0.09	-0.26	0.14	-0.13	0.11
10.0	-0.06	0.10	-0.07	0.09	-0.16	0.18	-0.07	0.11
10.5	-0.15	0.10	-0.13	0.09	-0.28	0.18	-0.17	0.10
10.1	-0.08	0.10	0.08	0.12	-0.17	0.16	-0.10	0.10
10.6	-0.13	0.10	-0.19	0.10	-0.22	0.16	-0.14	0.10
10.8	-0.11	0.10	-0.12	0.08	-0.23	0.16	-0.12	0.11
10.3	-0.13	0.10	-0.11	0.09	-0.32	0.15	-0.15	0.11

Analysis No.	Sample Name	Comment	$^{94}Zr/^{90}Zr$	2SE	$^{94}Zr/^{91}Zr$	2SE	$^{96}Zr/^{90}Zr$	2SE
MAY31C13	Penglai	Batch 1	0.358403	0.000011	1.62011	0.00007	0.059290	0.000003
MAY31C16	Penglai	Batch 1	0.358408	0.000011	1.62016	0.00006	0.059281	0.000004
MAY31C19	Penglai	Batch 1	0.358424	0.000012	1.62024	0.00007	0.059289	0.000005
MAY31C22	Penglai	Batch 1	0.358431	0.000014	1.62020	0.00005	0.059288	0.000005
MAY31C25	Penglai	Batch 1	0.358392	0.000014	1.62008	0.00007	0.059282	0.000005
MAY31C28	Penglai	Batch 1	0.358385	0.000011	1.62003	0.00006	0.059288	0.000004
MAY31C31	Penglai	Batch 1	0.358364	0.000012	1.61992	0.00007	0.059275	0.000005
MAY31D01	Penglai	Batch 1	0.358391	0.000012	1.62004	0.00007	0.059283	0.000004
MAY31D04	Penglai	Batch 1	0.358361	0.000012	1.62000	0.00006	0.059274	0.000004
MAY31D07	Penglai	Batch 1	0.358394	0.000013	1.62009	0.00007	0.059283	0.000004
MAY31D10	Penglai	Batch 1	0.358366	0.000014	1.62002	0.00006	0.059278	0.000004
MAY31D13	Penglai	Batch 1	0.358405	0.000015	1.62017	0.00006	0.059283	0.000004
MAY31D16	Penglai	Batch 1	0.358381	0.000011	1.62002	0.00006	0.059280	0.000005
MAY31D19	Penglai	Batch 1	0.358394	0.000013	1.62014	0.00007	0.059282	0.000004
MAY31D20	Penglai	Batch 1	0.358410	0.000011	1.62015	0.00006	0.059290	0.000004
MAY31D22	Penglai	Batch 1	0.358396	0.000014	1.62013	0.00007	0.059284	0.000003
MAY31D24	Penglai	Batch 1	0.358421	0.000012	1.62012	0.00008	0.059288	0.000004
MAY31D27	Penglai	Batch 1	0.358408	0.000014	1.62009	0.00006	0.059289	0.000004
MAY31D30	Penglai	Batch 1	0.358435	0.000015	1.62024	0.00008	0.059292	0.000004
MAY31D33	Penglai	Batch 1	0.358416	0.000012	1.62015	0.00007	0.059292	0.000003
MAY31D37	Penglai	Batch 1	0.358457	0.000011	1.62035	0.00006	0.059297	0.000004
MAY31D41	Penglai	Batch 1	0.358450	0.000013	1.62029	0.00006	0.059298	0.000004
MAY31D45	Penglai	Batch 1	0.358506	0.000014	1.62048	0.00006	0.059311	0.000004
MAY31E17	Penglai	Batch 1	0.358666	0.000010	1.62098	0.00007	0.059350	0.000005
MAY31E18	Penglai	Batch 1	0.358628	0.000012	1.62089	0.00006	0.059346	0.000005
MAY31E23	Penglai	Batch 1	0.358700	0.000012	1.62109	0.00006	0.059359	0.000004
MAY31E24	Penglai	Batch 1	0.358676	0.000010	1.62095	0.00006	0.059357	0.000004
MAY31E29	Penglai	Batch 1	0.358658	0.000012	1.62095	0.00005	0.059352	0.000005
MAY31E30	Penglai	Batch 1	0.358665	0.000010	1.62103	0.00006	0.059355	0.000004
MAY31E35	Penglai	Batch 1	0.358656	0.000013	1.62096	0.00007	0.059345	0.000004
MAY31E36	Penglai	Batch 1	0.358653	0.000013	1.62099	0.00006	0.059347	0.000004
MAY31E41	Penglai	Batch 1	0.358662	0.000013	1.62100	0.00005	0.059348	0.000005
MAY31E42	Penglai	Batch 1	0.358694	0.000010	1.62107	0.00006	0.059358	0.000004
MAY31E47	Penglai	Batch 1	0.358715	0.000012	1.62114	0.00006	0.059366	0.000004
MAY31E48	Penglai	Batch 1	0.358702	0.000012	1.62111	0.00006	0.059359	0.000004
MAY31E53	Penglai	Batch 1	0.358721	0.000010	1.62124	0.00004	0.059368	0.000004
MAY31E54	Penglai	Batch 1	0.358725	0.000013	1.62117	0.00006	0.059370	0.000005
MAY31E59	Penglai	Batch 1	0.358711	0.000012	1.62114	0.00007	0.059364	0.000004
MAY31E60	Penglai	Batch 1	0.358738	0.000013	1.62123	0.00005	0.059371	0.000005
MAY31E65	Penglai	Batch 1	0.358732	0.000012	1.62125	0.00005	0.059373	0.000005
MAY31E66	Penglai	Batch 1	0.358730	0.000011	1.62121	0.00006	0.059359	0.000003
MAY31F01	Penglai	Batch 1	0.358648	0.000013	1.62091	0.00007	0.059349	0.000004
MAY31F02	Penglai	Batch 1	0.358641	0.000014	1.62092	0.00006	0.059345	0.000004
MAY31F07	Penglai	Batch 1	0.358681	0.000014	1.62107	0.00007	0.059353	0.000004
MAY31F08	Penglai	Batch 1	0.358640	0.000015	1.62093	0.00008	0.059341	0.000005
MAY31F11	Penglai	Batch 1	0.358630	0.000012	1.62086	0.00007	0.059342	0.000003
MAY31F12	Penglai	Batch 1	0.358681	0.000011	1.62099	0.00005	0.059352	0.000004
MAY31F17	Penglai	Batch 1	0.358665	0.000012	1.62099	0.00007	0.059350	0.000005
MAY31F18	Penglai	Batch 1	0.358663	0.000011	1.62097	0.00006	0.059350	0.000004
MAY31F23	Penglai	Batch 1	0.358638	0.000015	1.62087	0.00007	0.059343	0.000004
MAY31F24	Penglai	Batch 1	0.358663	0.000014	1.62098	0.00006	0.059350	0.000005
MAY31F29	Penglai	Batch 1	0.358658	0.000013	1.62103	0.00007	0.059350	0.000004
MAY31F30	Penglai	Batch 1	0.358633	0.000014	1.62084	0.00007	0.059341	0.000004
MAY31F35	Penglai	Batch 1	0.358670	0.000013	1.62104	0.00007	0.059349	0.000005
MAY31F36	Penglai	Batch 1	0.358671	0.000012	1.62102	0.00006	0.059352	0.000004
MAY31F41	Penglai	Batch 1	0.358673	0.000012	1.62101	0.00007	0.059353	0.000004
MAY31F42	Penglai	Batch 1	0.358672	0.000011	1.62102	0.00006	0.059356	0.000004
MAY31F46	Penglai	Batch 1	0.358660	0.000011	1.62102	0.00006	0.059350	0.000004
MAY31F47	Penglai	Batch 1	0.358652	0.000012	1.62094	0.00006	0.059346	0.000005
MAY31F52	Penglai	Batch 1	0.358667	0.000016	1.62104	0.00008	0.059349	0.000005
MAY31F53	Penglai	Batch 1	0.358639	0.000012	1.62088	0.00006	0.059345	0.000004
JUN06A01	Penglai	Batch 1	0.358520	0.000014	1.62049	0.00007	0.059310	0.000004
JUN06A04	Penglai	Batch 1	0.358548	0.000012	1.62059	0.00006	0.059317	0.000003
JUN06A07	Penglai	Batch 1	0.358490	0.000016	1.62032	0.00007	0.059303	0.000005
JUN06A10	Penglai	Batch 1	0.358519	0.000011	1.62046	0.00005	0.059311	0.000004
JUN06A14	Penglai	Batch 1	0.358473	0.000014	1.62030	0.00008	0.059303	0.000004
JUN06A15	Penglai	Batch 1	0.358476	0.000018	1.62029	0.00007	0.059303	0.000005

^{90}Zr (V)	$\delta^{94/90}Zr_{GJ-1}$	2SE	$\delta^{94/91}Zr_{GJ-1}$	2SE	$\delta^{96/90}Zr_{GJ-1}$	2SE	$\delta^{94/90}Zr_{IPGP-Zr}$ †	2SE
10.1	-0.11	0.09	-0.12	0.09	-0.14	0.14	-0.13	0.10
10.2	-0.13	0.09	-0.12	0.09	-0.29	0.16	-0.14	0.10
10.3	-0.12	0.10	-0.09	0.09	-0.21	0.18	-0.13	0.11
10.0	-0.05	0.10	-0.06	0.08	-0.16	0.18	-0.06	0.11
10.3	-0.10	0.10	-0.09	0.09	-0.27	0.17	-0.11	0.11
10.3	-0.08	0.09	-0.06	0.08	-0.11	0.17	-0.09	0.10
10.8	-0.14	0.09	-0.14	0.09	-0.33	0.17	-0.15	0.10
10.5	-0.07	0.10	-0.09	0.09	-0.14	0.14	-0.08	0.10
11.3	-0.15	0.10	-0.13	0.09	-0.29	0.14	-0.16	0.11
10.4	-0.07	0.10	-0.08	0.09	-0.18	0.15	-0.08	0.11
10.6	-0.16	0.11	-0.15	0.09	-0.26	0.16	-0.17	0.11
10.3	-0.07	0.10	-0.05	0.09	-0.20	0.17	-0.09	0.11
10.7	-0.13	0.09	-0.14	0.09	-0.23	0.17	-0.14	0.10
10.9	-0.12	0.10	-0.10	0.09	-0.28	0.15	-0.14	0.11
10.6	-0.08	0.10	-0.09	0.09	-0.14	0.15	-0.09	0.11
11.4	-0.12	0.10	-0.11	0.09	-0.24	0.14	-0.13	0.11
10.5	-0.09	0.10	-0.09	0.10	-0.23	0.17	-0.10	0.11
10.8	-0.15	0.11	-0.15	0.09	-0.25	0.16	-0.16	0.12
10.9	-0.08	0.11	-0.07	0.10	-0.21	0.14	-0.09	0.11
10.3	-0.16	0.09	-0.16	0.09	-0.27	0.14	-0.18	0.10
10.3	-0.10	0.10	-0.08	0.08	-0.22	0.15	-0.11	0.10
10.2	-0.19	0.10	-0.16	0.08	-0.33	0.15	-0.20	0.11
10.1	-0.03	0.10	-0.04	0.08	-0.11	0.15	-0.04	0.11
9.9	-0.11	0.09	-0.10	0.08	-0.28	0.17	-0.12	0.10
10.5	-0.22	0.09	-0.16	0.08	-0.33	0.17	-0.23	0.10
10.1	-0.04	0.09	-0.05	0.08	-0.17	0.16	-0.05	0.10
10.3	-0.10	0.09	-0.13	0.08	-0.20	0.16	-0.11	0.10
10.3	-0.11	0.10	-0.12	0.08	-0.18	0.16	-0.12	0.11
10.2	-0.09	0.09	-0.07	0.08	-0.13	0.15	-0.10	0.10
10.4	-0.14	0.10	-0.13	0.08	-0.30	0.16	-0.15	0.11
10.3	-0.14	0.10	-0.11	0.08	-0.26	0.16	-0.16	0.11
10.2	-0.19	0.10	-0.15	0.07	-0.39	0.16	-0.21	0.10
10.4	-0.10	0.09	-0.10	0.08	-0.21	0.16	-0.12	0.10
10.4	-0.11	0.09	-0.12	0.08	-0.21	0.15	-0.12	0.10
10.3	-0.14	0.09	-0.14	0.08	-0.33	0.15	-0.16	0.10
9.7	-0.12	0.09	-0.07	0.08	-0.23	0.16	-0.13	0.10
9.5	-0.10	0.10	-0.12	0.08	-0.19	0.16	-0.12	0.11
9.6	-0.15	0.10	-0.14	0.09	-0.26	0.16	-0.16	0.10
9.4	-0.08	0.10	-0.09	0.08	-0.14	0.17	-0.09	0.10
9.8	-0.10	0.10	-0.07	0.08	-0.12	0.17	-0.11	0.10
9.4	-0.10	0.09	-0.10	0.08	-0.35	0.15	-0.11	0.10
9.6	-0.12	0.10	-0.13	0.09	-0.19	0.16	-0.13	0.11
9.5	-0.14	0.11	-0.12	0.09	-0.26	0.16	-0.15	0.11
9.3	-0.04	0.10	-0.03	0.09	-0.12	0.16	-0.05	0.11
9.9	-0.16	0.10	-0.12	0.09	-0.32	0.17	-0.17	0.11
10.2	-0.19	0.09	-0.15	0.09	-0.34	0.15	-0.20	0.10
9.4	-0.05	0.09	-0.08	0.09	-0.15	0.15	-0.06	0.10
9.8	-0.09	0.10	-0.08	0.09	-0.19	0.17	-0.10	0.11
9.8	-0.09	0.10	-0.09	0.09	-0.19	0.16	-0.10	0.11
9.7	-0.13	0.11	-0.14	0.09	-0.27	0.16	-0.15	0.11
9.9	-0.06	0.10	-0.07	0.09	-0.15	0.16	-0.08	0.11
10.0	-0.11	0.10	-0.07	0.10	-0.18	0.16	-0.12	0.11
10.0	-0.18	0.10	-0.19	0.10	-0.34	0.16	-0.19	0.11
9.6	-0.11	0.10	-0.09	0.09	-0.28	0.17	-0.12	0.11
9.5	-0.11	0.10	-0.10	0.09	-0.23	0.17	-0.12	0.10
9.7	-0.08	0.09	-0.09	0.09	-0.19	0.17	-0.09	0.10
9.5	-0.09	0.09	-0.09	0.08	-0.15	0.17	-0.10	0.10
9.9	-0.09	0.10	-0.07	0.09	-0.18	0.16	-0.10	0.11
9.4	-0.11	0.10	-0.12	0.09	-0.25	0.17	-0.13	0.11
9.0	-0.07	0.10	-0.06	0.09	-0.19	0.17	-0.08	0.11
9.5	-0.15	0.10	-0.16	0.09	-0.26	0.16	-0.16	0.11
10.0	-0.15	0.10	-0.13	0.09	-0.28	0.15	-0.16	0.11
9.6	-0.03	0.11	-0.02	0.09	-0.11	0.16	-0.04	0.11
9.8	-0.15	0.11	-0.15	0.09	-0.29	0.18	-0.16	0.11
10.3	-0.02	0.10	-0.03	0.09	-0.12	0.17	-0.04	0.11
10.0	-0.18	0.11	-0.15	0.10	-0.31	0.17	-0.19	0.12
10.0	-0.17	0.11	-0.16	0.10	-0.30	0.18	-0.19	0.12

Analysis No.	Sample Name	Comment	$^{94}Zr/^{90}Zr$	2SE	$^{94}Zr/^{91}Zr$	2SE	$^{96}Zr/^{90}Zr$	2SE
JUN06A19	Penglai	Batch 1	0.358551	0.000013	1.62056	0.00007	0.059318	0.000004
JUN06A23	Penglai	Batch 1	0.358555	0.000011	1.62054	0.00006	0.059319	0.000004
JUN06A25	Penglai	Batch 1	0.358560	0.000013	1.62060	0.00006	0.059323	0.000004
JUN06A29	Penglai	Batch 1	0.358531	0.000016	1.62048	0.00007	0.059316	0.000005
JUN06A33	Penglai	Batch 1	0.358539	0.000016	1.62053	0.00008	0.059317	0.000005
JUN06A37	Penglai	Batch 1	0.358524	0.000022	1.62048	0.00009	0.059313	0.000007
JUN06A39	Penglai	Batch 1	0.358526	0.000018	1.62045	0.00008	0.059316	0.000005
JUN06A45	Penglai	Batch 1	0.358506	0.000020	1.62041	0.00008	0.059306	0.000006
JUN06A49	Penglai	Batch 1	0.358540	0.000014	1.62051	0.00007	0.059318	0.000005
JUN06A53	Penglai	Batch 1	0.358503	0.000018	1.62041	0.00008	0.059306	0.000005
JUN06A55	Penglai	Batch 1	0.358547	0.000018	1.62046	0.00007	0.059314	0.000004
JUN06A59	Penglai	Batch 1	0.358508	0.000020	1.62041	0.00008	0.059308	0.000006
JUN06A63	Penglai	Batch 1	0.358575	0.000012	1.62064	0.00006	0.059322	0.000004
JUN06A67	Penglai	Batch 1	0.358492	0.000018	1.62042	0.00008	0.059300	0.000005
JUN06A73	Penglai	Batch 1	0.358599	0.000014	1.62077	0.00007	0.059331	0.000005
JUN06A77	Penglai	Batch 1	0.358576	0.000017	1.62068	0.00008	0.059326	0.000004
JUN06A81	Penglai	Batch 1	0.358638	0.000014	1.62086	0.00007	0.059340	0.000005
JUN06A83	Penglai	Batch 1	0.358627	0.000013	1.62085	0.00006	0.059343	0.000004
JUN06A87	Penglai	Batch 1	0.358596	0.000012	1.62069	0.00007	0.059334	0.000004
JUN06A91	Penglai	Batch 1	0.358580	0.000020	1.62066	0.00007	0.059327	0.000006
JUN06A95	Penglai	Batch 1	0.358634	0.000014	1.62081	0.00007	0.059337	0.000004
JUN06A97	Penglai	Batch 1	0.358629	0.000014	1.62083	0.00007	0.059341	0.000004
Average (±2SD)								
Ref. (16) *	Double spike							
Ref. (16) *	LA-MC-ICP-MS							
Penglai as an unknown								
MAY31D18	Penglai	Batch 1	0.358427	0.000013	1.62018	0.00006	0.059295	0.000004
MAY31D28	Penglai	Batch 1	0.358429	0.000012	1.62014	0.00006	0.059294	0.000004
MAY31D36	Penglai	Batch 1	0.358451	0.000012	1.62024	0.00006	0.059301	0.000004
MAY31E63	Penglai	Batch 1	0.358714	0.000012	1.62115	0.00006	0.059366	0.000004
MAY31F05	Penglai	Batch 1	0.358612	0.000012	1.62080	0.00006	0.059345	0.000004
MAY31F09	Penglai	Batch 1	0.358664	0.000013	1.62095	0.00008	0.059348	0.000004
MAY31F10	Penglai	Batch 1	0.358638	0.000012	1.62087	0.00006	0.059343	0.000003
MAY31F15	Penglai	Batch 1	0.358641	0.000012	1.62090	0.00005	0.059345	0.000004
MAY31F50	Penglai	Batch 1	0.358630	0.000013	1.62084	0.00007	0.059346	0.000004
JUN06B05	Penglai	Batch 1	0.358599	0.000017	1.62077	0.00007	0.059329	0.000004
JUN06B19	Penglai	Batch 1	0.358597	0.000013	1.62069	0.00006	0.059328	0.000003
JUN06B33	Penglai	Batch 1	0.358631	0.000015	1.62085	0.00007	0.059333	0.000004
JUN06B47	Penglai	Batch 1	0.358617	0.000016	1.62076	0.00008	0.059335	0.000004
JUN06B61	Penglai	Batch 1	0.358630	0.000013	1.62088	0.00006	0.059340	0.000004
JUN06B75	Penglai	Batch 1	0.358589	0.000009	1.62066	0.00006	0.059327	0.000004
JUN06B90	Penglai	Batch 1	0.358614	0.000012	1.62078	0.00006	0.059336	0.000003
JUN06C04	Penglai	Batch 1	0.358617	0.000014	1.62079	0.00006	0.059333	0.000003
JUN06C18	Penglai	Batch 1	0.358657	0.000009	1.62088	0.00006	0.059343	0.000004
JUN06C32	Penglai	Batch 1	0.358613	0.000015	1.62075	0.00007	0.059335	0.000004
JUN06C45	Penglai	Batch 1	0.358653	0.000016	1.62095	0.00006	0.059343	0.000004
JUN06C59	Penglai	Batch 1	0.358648	0.000013	1.62092	0.00007	0.059341	0.000004
JUN06C73	Penglai	Batch 1	0.358657	0.000014	1.62097	0.00006	0.059345	0.000004
JUN06C87	Penglai	Batch 1	0.358639	0.000016	1.62085	0.00007	0.059344	0.000005
JUN06C94	Penglai	Batch 1	0.358659	0.000018	1.62094	0.00008	0.059349	0.000005
JUN06D04	Penglai	Batch 1	0.358609	0.000016	1.62078	0.00008	0.059335	0.000005
JUN06D18	Penglai	Batch 1	0.358609	0.000013	1.62077	0.00006	0.059338	0.000004
JUN06D32	Penglai	Batch 1	0.358658	0.000017	1.62098	0.00008	0.059346	0.000004
JUN06D46	Penglai	Batch 1	0.358655	0.000017	1.62098	0.00006	0.059347	0.000004
JUN06D60	Penglai	Batch 1	0.358681	0.000014	1.62111	0.00007	0.059353	0.000004
JUN06D74	Penglai	Batch 1	0.358705	0.000015	1.62115	0.00007	0.059357	0.000004
JUN06D98	Penglai	Batch 1	0.358714	0.000013	1.62118	0.00006	0.059362	0.000004
Average (±2SD)								
Ref. (16) *	Double spike							
Ref. (16) *	LA-MC-ICP-MS							
91500 as an unknown								
MAY31B30	91500	Batch 1	0.358396	0.000015	1.62008	0.00006	0.059288	0.000004
MAY31B48	91500	Batch 1	0.358380	0.000014	1.62003	0.00006	0.059281	0.000004
JUN06A05	91500	Batch 1	0.358579	0.000017	1.62066	0.00008	0.059328	0.000005

^{90}Zr (V)	$\delta^{94/90}Zr_{GJ-1}$	2SE	$\delta^{94/91}Zr_{GJ-1}$	2SE	$\delta^{96/90}Zr_{GJ-1}$	2SE	$\delta^{94/90}Zr_{IPGP-Zr}$ †	2SE
9.5	-0.11	0.10	-0.09	0.09	-0.23	0.14	-0.12	0.11
9.4	-0.11	0.09	-0.12	0.08	-0.25	0.15	-0.13	0.10
9.4	-0.07	0.11	-0.06	0.09	-0.16	0.16	-0.08	0.11
9.9	-0.12	0.12	-0.11	0.10	-0.23	0.17	-0.13	0.12
10.0	-0.09	0.13	-0.09	0.12	-0.18	0.21	-0.10	0.14
10.0	-0.11	0.14	-0.09	0.12	-0.25	0.22	-0.12	0.15
10.2	-0.08	0.13	-0.09	0.11	-0.13	0.20	-0.09	0.14
9.9	-0.15	0.12	-0.13	0.11	-0.32	0.21	-0.17	0.13
9.4	-0.06	0.11	-0.07	0.10	-0.12	0.18	-0.07	0.12
10.0	-0.17	0.13	-0.11	0.11	-0.29	0.18	-0.18	0.13
9.4	-0.05	0.13	-0.08	0.10	-0.17	0.18	-0.06	0.14
9.9	-0.20	0.12	-0.17	0.10	-0.33	0.19	-0.21	0.13
9.3	0.01	0.11	-0.03	0.10	-0.04	0.17	0.00	0.12
10.1	-0.26	0.12	-0.21	0.11	-0.48	0.18	-0.27	0.13
9.2	-0.08	0.11	-0.07	0.10	-0.18	0.17	-0.09	0.12
9.7	-0.19	0.12	-0.16	0.11	-0.34	0.16	-0.20	0.12
9.5	-0.09	0.10	-0.10	0.10	-0.24	0.17	-0.10	0.11
9.7	-0.06	0.10	-0.05	0.09	-0.15	0.16	-0.08	0.11
9.7	-0.09	0.12	-0.09	0.10	-0.16	0.18	-0.10	0.12
10.1	-0.18	0.13	-0.15	0.10	-0.31	0.19	-0.19	0.13
9.7	-0.10	0.10	-0.11	0.09	-0.25	0.15	-0.11	0.11
9.8	-0.11	0.10	-0.09	0.09	-0.19	0.15	-0.13	0.11
	-0.11±0.09		-0.1±0.08		-0.23±0.14		-0.12±0.09	
	-		-		-		-0.119±0.040	
	-0.14±0.12		-0.10±0.09		-0.22±0.20		-	
11.3	-0.03	0.08	-0.04	0.09	0.01	0.16	-0.04	0.09
11.5	-0.12	0.09	-0.11	0.09	-0.16	0.16	-0.13	0.10
11.3	-0.10	0.07	-0.11	0.09	-0.11	0.14	-0.11	0.08
10.9	-0.18	0.07	-0.13	0.08	-0.22	0.16	-0.19	0.09
11.1	-0.25	0.08	-0.20	0.09	-0.25	0.16	-0.26	0.09
9.5	-0.12	0.08	-0.11	0.09	-0.21	0.16	-0.13	0.09
9.8	-0.20	0.08	-0.15	0.09	-0.29	0.16	-0.21	0.09
10.9	-0.19	0.07	-0.13	0.08	-0.28	0.15	-0.20	0.08
11.1	-0.21	0.08	-0.18	0.09	-0.25	0.16	-0.22	0.09
10.8	-0.24	0.09	-0.18	0.09	-0.37	0.15	-0.25	0.10
11.0	-0.11	0.09	-0.12	0.09	-0.19	0.16	-0.13	0.10
10.4	-0.05	0.09	-0.01	0.10	-0.16	0.15	-0.06	0.10
10.5	-0.22	0.09	-0.18	0.10	-0.26	0.15	-0.24	0.10
10.1	-0.16	0.09	-0.12	0.09	-0.21	0.16	-0.18	0.10
11.2	0.00	0.08	-0.01	0.09	-0.04	0.14	-0.01	0.09
11.2	-0.03	0.10	-0.01	0.10	-0.02	0.15	-0.04	0.11
11.2	-0.06	0.09	-0.05	0.09	-0.12	0.14	-0.07	0.10
10.1	-0.15	0.08	-0.16	0.09	-0.23	0.15	-0.17	0.09
11.0	-0.23	0.09	-0.19	0.10	-0.30	0.16	-0.24	0.10
9.8	-0.13	0.13	-0.11	0.12	-0.14	0.21	-0.14	0.14
10.0	-0.16	0.07	-0.09	0.09	-0.25	0.15	-0.17	0.08
10.0	-0.15	0.09	-0.10	0.10	-0.18	0.16	-0.17	0.10
11.1	-0.20	0.10	-0.16	0.11	-0.20	0.16	-0.22	0.11
10.4	-0.17	0.11	-0.12	0.11	-0.20	0.16	-0.18	0.12
11.0	-0.31	0.11	-0.24	0.11	-0.39	0.18	-0.33	0.11
11.5	-0.29	0.08	-0.21	0.10	-0.32	0.16	-0.31	0.09
10.8	-0.15	0.10	-0.10	0.10	-0.18	0.16	-0.16	0.11
10.8	-0.22	0.10	-0.18	0.10	-0.27	0.17	-0.23	0.10
10.7	-0.11	0.10	-0.07	0.11	-0.11	0.17	-0.12	0.11
10.2	-0.14	0.09	-0.07	0.09	-0.18	0.14	-0.15	0.10
10.4	-0.16	0.10	-0.09	0.11	-0.20	0.16	-0.17	0.10
	-0.16±0.15		-0.12±0.12		-0.2±0.18		-0.17±0.15	
	-		-		-		-0.119±0.040	
	-0.14±0.12		-0.10±0.09		-0.22±0.20		-	
12.0	-0.02	0.11	-0.01	0.09	-0.06	0.15	-0.04	0.11
11.6	-0.07	0.10	-0.07	0.09	-0.14	0.15	-0.08	0.11
9.6	0.06	0.11	0.03	0.09	0.09	0.17	0.05	0.12

Analysis No.	Sample Name	Comment	$^{94}Zr/^{90}Zr$	2SE	$^{94}Zr/^{91}Zr$	2SE	$^{96}Zr/^{90}Zr$	2SE
JUN06A40	91500	Batch 1	0.358575	0.000015	1.62059	0.00007	0.059325	0.000004
JUN06B03	91500	Batch 1	0.358690	0.000014	1.62103	0.00007	0.059354	0.000003
JUN06B88	91500	Batch 1	0.358606	0.000014	1.62072	0.00007	0.059334	0.000004
JUN06C02	91500	Batch 1	0.358621	0.000011	1.62080	0.00005	0.059337	0.000004
JUN06C92	91500	Batch 1	0.358688	0.000017	1.62102	0.00008	0.059352	0.000004
JUN06D02	91500	Batch 1	0.358641	0.000012	1.62088	0.00005	0.059341	0.000003
JUN06D96	91500	Batch 1	0.358767	0.000015	1.62137	0.00008	0.059375	0.000004
JUL19A005	91500	Batch 2	0.359812	0.000009	1.62493	0.00005	0.059642	0.000003
JUL19A008	91500	Batch 2	0.359855	0.000010	1.62512	0.00006	0.059645	0.000004
JUL19A029	91500	Batch 2	0.359794	0.000009	1.62491	0.00004	0.059633	0.000004
JUL19A050	91500	Batch 2	0.359766	0.000012	1.62475	0.00006	0.059629	0.000004
JUL19A066	91500	Batch 2	0.359729	0.000011	1.62465	0.00005	0.059620	0.000003
JUL19A090	91500	Batch 2	0.359722	0.000014	1.62459	0.00007	0.059617	0.000004
JUL19A093	91500	Batch 2	0.359730	0.000012	1.62461	0.00006	0.059621	0.000004
JUL19B002	91500	Batch 2	0.359724	0.000012	1.62463	0.00006	0.059620	0.000004
JUL19B023	91500	Batch 2	0.359739	0.000010	1.62468	0.00005	0.059623	0.000003
JUL19B045	91500	Batch 2	0.359740	0.000013	1.62469	0.00006	0.059620	0.000004
JUL19B066	91500	Batch 2	0.359740	0.000012	1.62466	0.00006	0.059625	0.000004
JUL19B087	91500	Batch 2	0.359744	0.000011	1.62470	0.00007	0.059624	0.000004
JUL19B091	91500	Batch 2	0.359731	0.000010	1.62463	0.00006	0.059622	0.000003
JUL19C002	91500	Batch 2	0.359728	0.000011	1.62462	0.00006	0.059619	0.000003
JUL19C023	91500	Batch 2	0.359721	0.000011	1.62461	0.00006	0.059619	0.000003
JUL19C027	91500	Batch 2	0.359719	0.000012	1.62455	0.00006	0.059616	0.000003
JUL19C048	91500	Batch 2	0.359705	0.000011	1.62457	0.00006	0.059617	0.000004
JUL19C069	91500	Batch 2	0.359722	0.000008	1.62461	0.00004	0.059615	0.000003
JUL19C090	91500	Batch 2	0.359728	0.000010	1.62461	0.00006	0.059618	0.000003
JUL20A031	91500	Batch 2	0.360064	0.000014	1.62575	0.00007	0.059708	0.000004
JUL22A003	91500	Batch 2	0.359638	0.000009	1.62421	0.00004	0.059599	0.000003
JUL22A024	91500	Batch 2	0.359679	0.000010	1.62440	0.00005	0.059609	0.000003
JUL22A045	91500	Batch 2	0.359723	0.000011	1.62455	0.00006	0.059620	0.000004
JUL22A066	91500	Batch 2	0.359818	0.000008	1.62490	0.00005	0.059646	0.000003
JUL22A074	91500	Batch 2	0.359851	0.000011	1.62493	0.00006	0.059651	0.000003
JUL22A088	91500	Batch 2	0.359836	0.000011	1.62491	0.00005	0.059651	0.000004
JUL22B03	91500	Batch 2	0.359699	0.000015	1.62447	0.00006	0.059618	0.000004
JUL22B05	91500	Batch 2	0.359682	0.000013	1.62436	0.00007	0.059609	0.000004
JUL22B63	91500	Batch 2	0.359427	0.000018	1.62355	0.00007	0.059549	0.000004
JUL22B94	91500	Batch 2	0.359360	0.000013	1.62331	0.00007	0.059529	0.000004
JUL22C03	91500	Batch 2	0.359376	0.000011	1.62336	0.00005	0.059535	0.000003
JUL22C25	91500	Batch 2	0.359357	0.000015	1.62329	0.00006	0.059528	0.000004
JUL22C46	91500	Batch 2	0.359301	0.000014	1.62307	0.00007	0.059518	0.000003
JUL22C68	91500	Batch 2	0.359267	0.000011	1.62296	0.00006	0.059507	0.000003
JUL22C72	91500	Batch 2	0.359293	0.000012	1.62308	0.00005	0.059513	0.000004
JUL22C86	91500	Batch 2	0.359273	0.000010	1.62301	0.00006	0.059505	0.000003
JUL23A02	91500	Batch 2	0.359303	0.000011	1.62308	0.00006	0.059517	0.000003
JUL23A08	91500	Batch 2	0.359271	0.000011	1.62300	0.00006	0.059510	0.000003
JUL23A29	91500	Batch 2	0.359265	0.000012	1.62300	0.00006	0.059505	0.000003
JUL23A51	91500	Batch 2	0.359316	0.000010	1.62313	0.00006	0.059518	0.000003
JUL23A73	91500	Batch 2	0.359265	0.000010	1.62295	0.00005	0.059504	0.000003
JUL23A83	91500	Batch 2	0.359266	0.000010	1.62299	0.00006	0.059504	0.000003
JUL23B27	91500	Batch 2	0.359446	0.000015	1.62359	0.00006	0.059553	0.000004
JUL23B48	91500	Batch 2	0.359477	0.000012	1.62369	0.00005	0.059557	0.000004
JUL23B75	91500	Batch 2	0.359456	0.000014	1.62363	0.00006	0.059553	0.000004
JUL23B23	91500	Batch 2	0.359280	0.000013	1.62305	0.00006	0.059511	0.000004
Average (±2SD)								
Ref. (16) *	Double spike							
Ref. (16) *	LA-MC-ICP-MS							
Plešovice as an unknown								
MAY31B46	Plešovice	Batch 1	0.358438	0.000010	1.62026	0.00005	0.059292	0.000004
MAY31B62	Plešovice	Batch 1	0.358437	0.000013	1.62020	0.00007	0.059291	0.000004
MAY31B95	Plešovice	Batch 1	0.358491	0.000012	1.62035	0.00006	0.059305	0.000004
MAY31D03	Plešovice	Batch 1	0.358484	0.000011	1.62034	0.00006	0.059307	0.000004
MAY31D26	Plešovice	Batch 1	0.358545	0.000012	1.62057	0.00006	0.059321	0.000004
MAY31D35	Plešovice	Batch 1	0.358557	0.000014	1.62062	0.00007	0.059321	0.000004
MAY31D43	Plešovice	Batch 1	0.358530	0.000011	1.62053	0.00005	0.059312	0.000003
MAY31E62	Plešovice	Batch 1	0.358829	0.000010	1.62153	0.00006	0.059395	0.000003

^{90}Zr (V)	$\delta^{94/90}Zr_{GJ-1}$	2SE	$\delta^{94/91}Zr_{GJ-1}$	2SE	$\delta^{96/90}Zr_{GJ-1}$	2SE	$\delta^{94/90}Zr_{IPGP-Zr}$ †	2SE
9.7	0.06	0.13	0.00	0.11	0.02	0.20	0.05	0.14
10.6	0.01	0.09	-0.01	0.09	0.05	0.13	0.00	0.10
11.6	-0.05	0.10	-0.05	0.10	-0.06	0.15	-0.06	0.11
12.0	-0.05	0.09	-0.05	0.09	-0.05	0.14	-0.06	0.10
10.5	-0.09	0.11	-0.07	0.10	-0.15	0.15	-0.10	0.11
11.3	-0.23	0.10	-0.18	0.10	-0.29	0.17	-0.24	0.11
10.4	-0.01	0.10	0.02	0.11	0.01	0.17	-0.03	0.11
12.9	-0.15	0.07	-0.10	0.08	-0.15	0.13	-0.16	0.08
11.7	-0.03	0.06	-0.01	0.08	-0.07	0.15	-0.04	0.07
13.1	-0.08	0.06	-0.02	0.08	-0.15	0.13	-0.09	0.07
13.8	-0.04	0.06	-0.11	0.08	-0.08	0.11	-0.05	0.07
13.7	-0.10	0.07	-0.07	0.08	-0.12	0.13	-0.12	0.08
13.8	-0.07	0.07	-0.08	0.09	-0.18	0.14	-0.08	0.09
13.8	-0.02	0.07	-0.05	0.08	-0.05	0.13	-0.03	0.08
13.9	-0.10	0.07	-0.08	0.08	-0.15	0.13	-0.11	0.08
13.8	0.00	0.06	0.00	0.08	0.01	0.14	-0.01	0.08
13.5	-0.02	0.07	0.00	0.08	-0.02	0.14	-0.03	0.08
13.3	-0.04	0.06	-0.06	0.08	-0.08	0.13	-0.05	0.08
13.9	0.03	0.07	0.01	0.08	0.02	0.12	0.01	0.08
13.9	-0.06	0.07	-0.04	0.08	-0.12	0.12	-0.07	0.08
14.0	-0.07	0.07	-0.07	0.07	-0.17	0.13	-0.09	0.08
14.3	0.05	0.07	0.00	0.08	0.03	0.12	0.04	0.08
14.3	-0.03	0.07	-0.06	0.08	-0.09	0.15	-0.04	0.09
14.3	-0.05	0.07	-0.03	0.07	-0.07	0.13	-0.06	0.08
14.3	-0.03	0.07	-0.01	0.07	-0.06	0.14	-0.04	0.08
14.5	-0.01	0.08	-0.02	0.08	0.00	0.14	-0.02	0.09
12.7	0.00	0.07	-0.03	0.08	0.03	0.13	-0.02	0.08
14.3	0.06	0.07	0.00	0.08	0.07	0.12	0.05	0.08
14.2	-0.06	0.09	-0.04	0.08	-0.10	0.18	-0.08	0.10
13.7	-0.18	0.09	-0.12	0.10	-0.30	0.17	-0.19	0.10
13.4	0.00	0.07	0.01	0.08	0.00	0.13	-0.01	0.08
13.4	-0.01	0.08	-0.06	0.10	0.01	0.13	-0.02	0.09
13.0	-0.05	0.10	-0.05	0.10	-0.05	0.18	-0.06	0.11
13.4	-0.02	0.07	-0.01	0.08	0.00	0.13	-0.03	0.08
13.5	-0.05	0.07	-0.08	0.09	-0.12	0.16	-0.06	0.09
14.9	-0.04	0.08	-0.03	0.08	-0.02	0.13	-0.05	0.09
15.8	-0.12	0.07	-0.08	0.08	-0.23	0.12	-0.13	0.08
15.7	-0.10	0.07	-0.08	0.07	-0.13	0.12	-0.11	0.08
15.8	-0.05	0.07	-0.04	0.08	-0.10	0.13	-0.06	0.08
15.9	-0.11	0.07	-0.12	0.07	-0.09	0.13	-0.13	0.08
16.6	-0.13	0.06	-0.13	0.07	-0.15	0.11	-0.14	0.07
16.6	-0.12	0.07	-0.10	0.08	-0.16	0.13	-0.13	0.08
16.6	-0.15	0.06	-0.13	0.07	-0.25	0.11	-0.16	0.07
16.5	-0.11	0.07	-0.10	0.07	-0.11	0.12	-0.12	0.08
16.6	-0.09	0.06	-0.07	0.07	-0.08	0.11	-0.10	0.07
16.0	-0.15	0.06	-0.11	0.08	-0.18	0.12	-0.16	0.08
16.6	-0.02	0.05	-0.04	0.06	0.00	0.11	-0.03	0.07
16.7	-0.05	0.06	-0.06	0.07	-0.08	0.13	-0.06	0.08
16.6	-0.08	0.07	-0.06	0.08	-0.16	0.12	-0.10	0.08
15.1	-0.11	0.07	-0.08	0.08	-0.19	0.12	-0.12	0.08
14.6	-0.02	0.06	-0.01	0.07	-0.05	0.13	-0.03	0.07
14.6	-0.15	0.07	-0.10	0.07	-0.22	0.13	-0.16	0.08
16.2	-0.38	0.08	-0.28	0.08	-0.58	0.13	-0.39	0.09
	-0.06±0.12		-0.05±0.09		-0.09±0.18		-0.07±0.12	
	-		-		-		-0.028±0.038	
	0.00±0.11		-0.01±0.09		0.00±0.17		-	
10.2	0.08	0.10	0.05	0.09	0.04	0.15	0.07	0.11
10.3	0.14	0.10	0.09	0.09	0.09	0.15	0.13	0.10
10.2	0.19	0.10	0.11	0.09	0.17	0.17	0.18	0.10
10.5	0.19	0.09	0.10	0.09	0.26	0.14	0.18	0.10
10.3	0.26	0.10	0.19	0.09	0.33	0.17	0.25	0.11
10.1	0.23	0.10	0.13	0.09	0.23	0.14	0.22	0.10
10.3	0.04	0.10	-0.01	0.08	-0.08	0.14	0.03	0.11
10.0	0.17	0.09	0.10	0.08	0.25	0.15	0.16	0.10

Analysis No.	Sample Name	Comment	$^{94}Zr/^{90}Zr$	2SE	$^{94}Zr/^{91}Zr$	2SE	$^{96}Zr/^{90}Zr$	2SE
MAY31F04	Plešovice	Batch 1	0.358743	0.000012	1.62119	0.00007	0.059370	0.000004
MAY31F14	Plešovice	Batch 1	0.358758	0.000012	1.62129	0.00007	0.059373	0.000004
MAY31F49	Plešovice	Batch 1	0.358717	0.000011	1.62121	0.00005	0.059367	0.000004
JUN06A06	Plešovice	Batch 1	0.358560	0.000013	1.62058	0.00007	0.059323	0.000004
JUN06B04	Plešovice	Batch 1	0.358722	0.000012	1.62115	0.00006	0.059358	0.000004
JUN06B89	Plešovice	Batch 1	0.358678	0.000012	1.62095	0.00005	0.059353	0.000004
JUN06C03	Plešovice	Batch 1	0.358697	0.000009	1.62101	0.00005	0.059355	0.000003
JUN06C93	Plešovice	Batch 1	0.358762	0.000014	1.62129	0.00006	0.059374	0.000004
JUN06D03	Plešovice	Batch 1	0.358771	0.000015	1.62131	0.00007	0.059377	0.000004
JUN06D97	Plešovice	Batch 1	0.358857	0.000013	1.62161	0.00006	0.059386	0.000003
Average (±2SD)								
Ref. (15) *	Double spike							
Ref. (16) *	LA-MC-ICP-MS							
Zr-Paki as an unknown								
JUL19A006	Zr-Paki	Batch 2	0.359898	0.000010	1.62522	0.00005	0.059659	0.000003
JUL19A009	Zr-Paki	Batch 2	0.359889	0.000011	1.62520	0.00006	0.059656	0.000003
JUL19A030	Zr-Paki	Batch 2	0.359833	0.000011	1.62495	0.00005	0.059646	0.000003
JUL19A051	Zr-Paki	Batch 2	0.359819	0.000008	1.62590	0.00023	0.059641	0.000004
JUL19A067	Zr-Paki	Batch 2	0.359786	0.000011	1.62485	0.00005	0.059632	0.000003
JUL19A091	Zr-Paki	Batch 2	0.359791	0.000010	1.62483	0.00005	0.059635	0.000003
JUL19A094	Zr-Paki	Batch 2	0.359789	0.000010	1.62484	0.00005	0.059634	0.000004
JUL19B003	Zr-Paki	Batch 2	0.359795	0.000011	1.62486	0.00005	0.059631	0.000004
JUL19B024	Zr-Paki	Batch 2	0.359769	0.000010	1.62479	0.00006	0.059628	0.000003
JUL19B046	Zr-Paki	Batch 2	0.359763	0.000011	1.62477	0.00005	0.059636	0.000003
JUL19B067	Zr-Paki	Batch 2	0.359796	0.000011	1.62483	0.00005	0.059633	0.000003
JUL19B088	Zr-Paki	Batch 2	0.359774	0.000011	1.62477	0.00006	0.059632	0.000003
JUL19B092	Zr-Paki	Batch 2	0.359774	0.000011	1.62477	0.00005	0.059631	0.000003
JUL19C003	Zr-Paki	Batch 2	0.359789	0.000011	1.62483	0.00006	0.059635	0.000003
JUL19C024	Zr-Paki	Batch 2	0.359795	0.000011	1.62482	0.00006	0.059638	0.000003
JUL19C028	Zr-Paki	Batch 2	0.359783	0.000010	1.62481	0.00005	0.059632	0.000003
JUL19C049	Zr-Paki	Batch 2	0.359780	0.000010	1.62479	0.00006	0.059635	0.000004
JUL19C070	Zr-Paki	Batch 2	0.359788	0.000009	1.62482	0.00005	0.059636	0.000003
JUL19C091	Zr-Paki	Batch 2	0.359791	0.000010	1.62483	0.00005	0.059633	0.000004
JUL20A032	Zr-Paki	Batch 2	0.360111	0.000012	1.62593	0.00006	0.059719	0.000003
JUL22A004	Zr-Paki	Batch 2	0.359690	0.000013	1.62440	0.00006	0.059614	0.000003
JUL22A025	Zr-Paki	Batch 2	0.359767	0.000011	1.62466	0.00007	0.059631	0.000003
JUL22A046	Zr-Paki	Batch 2	0.359830	0.000010	1.62489	0.00006	0.059645	0.000004
JUL22A067	Zr-Paki	Batch 2	0.359847	0.000016	1.62493	0.00007	0.059653	0.000005
JUL22A075	Zr-Paki	Batch 2	0.359878	0.000013	1.62507	0.00006	0.059660	0.000005
JUL22A089	Zr-Paki	Batch 2	0.359941	0.000014	1.62528	0.00006	0.059672	0.000004
JUL22B06	Zr-Paki	Batch 2	0.359785	0.000009	1.62476	0.00005	0.059638	0.000003
JUL22B64	Zr-Paki	Batch 2	0.359545	0.000011	1.62393	0.00005	0.059576	0.000003
JUL22B95	Zr-Paki	Batch 2	0.359491	0.000011	1.62374	0.00006	0.059561	0.000003
JUL22C04	Zr-Paki	Batch 2	0.359482	0.000010	1.62373	0.00005	0.059560	0.000003
JUL22C26	Zr-Paki	Batch 2	0.359444	0.000009	1.62358	0.00006	0.059553	0.000003
JUL22C47	Zr-Paki	Batch 2	0.359417	0.000010	1.62354	0.00004	0.059544	0.000003
JUL22C69	Zr-Paki	Batch 2	0.359391	0.000010	1.62344	0.00005	0.059536	0.000003
JUL22C73	Zr-Paki	Batch 2	0.359400	0.000010	1.62342	0.00005	0.059543	0.000003
JUL22C87	Zr-Paki	Batch 2	0.359393	0.000012	1.62336	0.00005	0.059533	0.000003
JUL23A03	Zr-Paki	Batch 2	0.359380	0.000011	1.62337	0.00006	0.059535	0.000003
JUL23A09	Zr-Paki	Batch 2	0.359395	0.000009	1.62346	0.00005	0.059539	0.000003
JUL23A30	Zr-Paki	Batch 2	0.359392	0.000009	1.62343	0.00005	0.059536	0.000003
JUL23A52	Zr-Paki	Batch 2	0.359390	0.000011	1.62341	0.00006	0.059534	0.000003
JUL23A74	Zr-Paki	Batch 2	0.359363	0.000010	1.62327	0.00005	0.059531	0.000002
JUL23A84	Zr-Paki	Batch 2	0.359381	0.000011	1.62335	0.00005	0.059534	0.000003
JUL23B24	Zr-Paki	Batch 2	0.359410	0.000012	1.62351	0.00005	0.059542	0.000003
JUL23B28	Zr-Paki	Batch 2	0.359515	0.000011	1.62385	0.00006	0.059572	0.000003
JUL23B49	Zr-Paki	Batch 2	0.359545	0.000013	1.62391	0.00006	0.059577	0.000003
JUL23B76	Zr-Paki	Batch 2	0.359521	0.000010	1.62387	0.00005	0.059570	0.000003
Average (±2SD)								
Ref. (16) *	LA-MC-ICP-MS							

* Errors for the reference values are given at 2SD level. † The $\delta^{94}Zr$ of GJ-1 relative to the IPGP-Zr standard is -0.012 ± 0.042‰ (2SD).

^{90}Zr (V)	$\delta^{94/90}Zr_{GJ-1}$	2SE	$\delta^{94/91}Zr_{GJ-1}$	2SE	$\delta^{96/90}Zr_{GJ-1}$	2SE	$\delta^{94/90}Zr_{IPGP-Zr}$ †	2SE
9.9	0.15	0.10	0.04	0.09	0.17	0.16	0.13	0.11
9.9	0.17	0.09	0.11	0.09	0.20	0.15	0.16	0.10
10.2	0.07	0.10	0.05	0.09	0.11	0.16	0.06	0.11
10.7	0.01	0.11	-0.02	0.09	0.00	0.16	-0.01	0.11
10.2	0.10	0.09	0.06	0.09	0.12	0.14	0.09	0.10
11.1	0.15	0.10	0.09	0.10	0.26	0.15	0.14	0.11
11.3	0.16	0.08	0.08	0.09	0.25	0.14	0.15	0.09
10.3	0.12	0.10	0.10	0.10	0.22	0.15	0.11	0.11
10.2	0.14	0.11	0.09	0.10	0.30	0.18	0.12	0.11
9.9	0.24	0.10	0.17	0.11	0.20	0.16	0.23	0.10
	0.14±0.13		0.08±0.11		0.17±0.22		0.13±0.13	
	-		-		-		0.106±0.043	
	0.14±0.10		0.10±0.09		0.21±0.19		-	
12.3	0.12	0.09	0.07	0.08	0.14	0.13	0.11	0.10
12.1	0.10	0.08	0.04	0.08	0.10	0.14	0.09	0.09
12.4	0.06	0.09	0.00	0.08	0.07	0.13	0.05	0.10
12.0	0.14	0.08	0.60	0.15	0.11	0.11	0.13	0.09
12.7	0.09	0.09	0.05	0.08	0.08	0.12	0.08	0.10
13.0	0.16	0.09	0.06	0.08	0.12	0.13	0.15	0.10
13.1	0.18	0.09	0.09	0.08	0.18	0.13	0.17	0.10
13.2	0.09	0.07	0.06	0.08	0.04	0.13	0.08	0.08
13.1	0.09	0.06	0.07	0.08	0.09	0.14	0.07	0.08
12.7	0.05	0.07	0.05	0.07	0.23	0.13	0.03	0.08
12.4	0.12	0.06	0.05	0.07	0.06	0.13	0.11	0.08
12.8	0.11	0.07	0.05	0.08	0.17	0.12	0.10	0.08
12.8	0.06	0.07	0.05	0.07	0.03	0.12	0.05	0.08
13.1	0.10	0.07	0.06	0.07	0.09	0.13	0.08	0.08
13.2	0.26	0.07	0.13	0.08	0.35	0.12	0.25	0.08
13.3	0.14	0.07	0.11	0.08	0.19	0.15	0.13	0.08
13.3	0.16	0.07	0.10	0.07	0.24	0.13	0.15	0.08
13.2	0.15	0.07	0.13	0.07	0.29	0.13	0.14	0.08
13.3	0.17	0.08	0.12	0.08	0.25	0.14	0.16	0.09
11.5	0.13	0.07	0.08	0.08	0.21	0.12	0.12	0.08
9.9	0.20	0.08	0.12	0.08	0.32	0.12	0.19	0.09
10.1	0.18	0.09	0.12	0.09	0.27	0.18	0.17	0.10
9.8	0.12	0.09	0.09	0.10	0.13	0.17	0.11	0.10
9.4	0.08	0.08	0.03	0.09	0.11	0.14	0.07	0.09
9.6	0.07	0.09	0.03	0.10	0.16	0.14	0.06	0.10
8.9	0.24	0.10	0.17	0.10	0.31	0.18	0.23	0.11
13.6	0.23	0.07	0.17	0.08	0.36	0.16	0.22	0.08
15.5	0.29	0.07	0.21	0.07	0.43	0.13	0.27	0.08
15.9	0.25	0.07	0.18	0.08	0.32	0.12	0.23	0.08
16.0	0.20	0.07	0.14	0.07	0.29	0.12	0.19	0.08
16.0	0.19	0.06	0.13	0.07	0.32	0.12	0.18	0.07
16.2	0.21	0.06	0.17	0.06	0.35	0.13	0.20	0.08
16.0	0.22	0.06	0.17	0.07	0.33	0.11	0.21	0.07
16.0	0.18	0.07	0.11	0.08	0.33	0.12	0.16	0.08
15.7	0.19	0.06	0.09	0.07	0.22	0.11	0.18	0.07
16.1	0.11	0.07	0.08	0.07	0.18	0.12	0.10	0.08
15.8	0.26	0.05	0.21	0.06	0.41	0.11	0.24	0.07
16.1	0.21	0.06	0.15	0.08	0.35	0.12	0.19	0.07
15.7	0.19	0.06	0.13	0.06	0.27	0.11	0.18	0.07
16.1	0.22	0.06	0.14	0.07	0.38	0.12	0.21	0.07
16.0	0.24	0.07	0.16	0.07	0.35	0.12	0.22	0.08
15.8	-0.02	0.07	0.01	0.07	-0.06	0.13	-0.03	0.08
15.0	0.09	0.06	0.08	0.07	0.13	0.11	0.07	0.07
14.7	0.17	0.06	0.12	0.07	0.28	0.13	0.16	0.07
14.8	0.03	0.07	0.05	0.07	0.06	0.12	0.02	0.08
	0.15±0.14		0.11±0.18		0.21±0.24		0.14±0.14	
	0.24±0.11		-		0.37±0.20		-	

Table S8

LA-ICP-MS U-Pb isotope and Ti concentration data of standard zircons

Analysis No.	Pb	Th	U	U-Pb Ratios						
	ppm	ppm	ppm	$^{207}Pb/^{206}Pb$	1σ	$^{207}Pb/^{235}U$	1σ	$^{206}Pb/^{238}U$	1σ	rho
Standard zircon 91500 as an external standard										
AUG22A02	15.2	23.1	67.1	0.0735	0.0026	1.8305	0.0646	0.1803	0.0020	0.32
AUG22A03	14.4	22.1	64.5	0.0763	0.0028	1.8699	0.0682	0.1780	0.0018	0.27
AUG22A15	16.6	26.7	76.5	0.0748	0.0022	1.8513	0.0548	0.1794	0.0021	0.40
AUG22A16	16.8	26.9	76.8	0.0749	0.0023	1.8491	0.0550	0.1789	0.0018	0.35
AUG22A25	14.7	23.2	68.2	0.0757	0.0025	1.8629	0.0628	0.1784	0.0019	0.32
AUG22A26	14.5	22.7	66.3	0.0740	0.0024	1.8375	0.0620	0.1799	0.0019	0.32
AUG22A35	17.1	27.6	79.1	0.0760	0.0025	1.8388	0.0577	0.1757	0.0021	0.38
AUG22A36	16.1	25.4	72.3	0.0738	0.0022	1.8616	0.0554	0.1827	0.0022	0.40
AUG22A45	13.6	20.8	61.4	0.0762	0.0024	1.8679	0.0562	0.1778	0.0023	0.42
AUG22A46	15.3	23.8	69.4	0.0735	0.0023	1.8325	0.0549	0.1805	0.0021	0.40
AUG22A55	15.7	24.3	69.7	0.0746	0.0025	1.8347	0.0641	0.1780	0.0023	0.37
AUG22A56	15.7	24.3	69.4	0.0752	0.0026	1.8657	0.0655	0.1804	0.0021	0.33
AUG22A65	17.4	27.6	77.7	0.0744	0.0023	1.8540	0.0562	0.1806	0.0022	0.40
AUG22A66	16.8	26.7	76.5	0.0754	0.0023	1.8464	0.0566	0.1778	0.0022	0.41
AUG22A72	16.3	25.8	74.9	0.0766	0.0028	1.8807	0.0677	0.1774	0.0027	0.43
AUG22A73	16.5	25.4	74.4	0.0731	0.0024	1.8197	0.0588	0.1809	0.0025	0.42
AUG22B02	16.6	27.1	75.6	0.0762	0.0027	1.8632	0.0660	0.1774	0.0023	0.36
AUG22B03	16.1	25.7	72.5	0.0736	0.0023	1.8372	0.0581	0.1809	0.0021	0.37
AUG22B15	17.8	28.2	80.3	0.0751	0.0022	1.8647	0.0533	0.1805	0.0022	0.43
AUG22B16	17.4	28.0	79.8	0.0747	0.0023	1.8357	0.0576	0.1778	0.0021	0.37
AUG22B25	16.3	25.9	73.9	0.0760	0.0024	1.8666	0.0589	0.1778	0.0020	0.35
AUG22B26	16.5	26.0	73.9	0.0737	0.0023	1.8338	0.0559	0.1805	0.0021	0.39
AUG22B35	16.9	26.3	76.3	0.0739	0.0022	1.8264	0.0564	0.1791	0.0022	0.40
AUG22B36	16.9	26.6	76.8	0.0759	0.0023	1.8740	0.0591	0.1792	0.0023	0.41
AUG22B45	16.8	26.5	76.1	0.0726	0.0025	1.7911	0.0574	0.1793	0.0025	0.43
AUG22B46	17.0	26.7	76.3	0.0772	0.0024	1.9093	0.0567	0.1791	0.0023	0.42
AUG22B55	18.3	29.3	82.8	0.0752	0.0024	1.8524	0.0614	0.1796	0.0022	0.36
AUG22B56	17.2	26.1	78.5	0.0746	0.0023	1.8480	0.0615	0.1788	0.0023	0.38
AUG22B65	16.0	25.6	72.3	0.0741	0.0024	1.8336	0.0584	0.1790	0.0023	0.40
AUG22B66	16.5	25.8	74.5	0.0757	0.0025	1.8668	0.0600	0.1794	0.0025	0.43
AUG22B75	17.3	27.4	77.8	0.0773	0.0026	1.9116	0.0655	0.1794	0.0017	0.28
AUG22B76	17.3	27.3	77.7	0.0724	0.0025	1.7888	0.0637	0.1789	0.0017	0.26
AUG22B85	16.4	25.7	75.0	0.0750	0.0025	1.8442	0.0610	0.1784	0.0019	0.32
AUG22B86	16.4	25.8	74.1	0.0748	0.0024	1.8562	0.0584	0.1800	0.0019	0.34
AUG22B97	17.0	26.8	75.8	0.0738	0.0024	1.8346	0.0594	0.1804	0.0021	0.35
AUG22B98	16.8	26.9	76.3	0.0760	0.0025	1.8658	0.0607	0.1780	0.0020	0.34
JUL17A02	16.2	28.2	74.6	0.0739	0.0023	1.8302	0.0556	0.1797	0.0020	0.09
JUL17A03	16.4	28.3	76.0	0.0758	0.0024	1.8702	0.0581	0.1787	0.0020	0.09
JUL17A15	16.4	28.0	74.9	0.0754	0.0024	1.8915	0.0574	0.1808	0.0022	0.09
JUL17A16	16.6	28.1	76.7	0.0743	0.0024	1.8089	0.0542	0.1775	0.0022	0.09
JUL17A25	16.6	28.5	76.1	0.0750	0.0024	1.8563	0.0580	0.1790	0.0020	0.09
JUL17A26	16.6	28.3	76.3	0.0747	0.0021	1.8441	0.0506	0.1794	0.0022	0.09
JUL17A37	16.3	27.8	75.7	0.0748	0.0024	1.8487	0.0579	0.1785	0.0019	0.09
JUL17A38	16.2	27.4	75.3	0.0749	0.0026	1.8517	0.0593	0.1798	0.0021	0.00
JUL17A47	16.1	27.5	74.7	0.0744	0.0024	1.8585	0.0598	0.1802	0.0019	0.18
JUL17A48	14.8	25.3	69.5	0.0754	0.0024	1.8419	0.0569	0.1781	0.0021	0.03
JUL17A59	16.2	27.3	73.8	0.0730	0.0023	1.8342	0.0567	0.1820	0.0020	0.12
JUL17A60	15.8	27.1	73.9	0.0767	0.0025	1.8662	0.0596	0.1763	0.0019	0.09
JUL17A69	16.3	27.4	75.0	0.0745	0.0024	1.8488	0.0579	0.1788	0.0022	0.12
JUL17A70	16.4	28.0	75.4	0.0752	0.0025	1.8516	0.0589	0.1796	0.0028	0.10
JUL17A81	16.4	28.0	75.4	0.0753	0.0026	1.8536	0.0609	0.1789	0.0020	0.09
JUL17A82	16.4	27.4	75.0	0.0745	0.0025	1.8468	0.0602	0.1794	0.0019	0.10
JUL17A89	15.2	24.8	68.7	0.0754	0.0023	1.8831	0.0545	0.1810	0.0022	0.09
JUL17A90	15.2	25.5	70.5	0.0743	0.0023	1.8173	0.0548	0.1774	0.0023	0.17
JUL18B02	14.7	23.9	67.4	0.0772	0.0026	1.8886	0.0605	0.1781	0.0023	0.09
JUL18B03	14.0	22.1	64.1	0.0725	0.0025	1.8118	0.0608	0.1803	0.0021	0.15
JUL18B15	15.8	25.7	69.9	0.0745	0.0022	1.8641	0.0533	0.1818	0.0018	0.12
JUL18B16	15.6	26.1	71.1	0.0753	0.0023	1.8363	0.0564	0.1765	0.0019	0.17
JUL18B26	15.4	25.7	69.6	0.0749	0.0026	1.8502	0.0612	0.1792	0.0021	0.03
JUL18B37	16.1	27.4	74.2	0.0754	0.0026	1.8298	0.0590	0.1758	0.0019	0.00
JUL18B38	16.4	27.1	72.9	0.0743	0.0022	1.8706	0.0528	0.1825	0.0021	0.10

U-Pb Ages (Ma)					Ti
$^{207}Pb/^{235}U$	1σ	$^{206}Pb/^{238}U$	1σ	Conc.% *	ppm
1056	23	1069	11	101	4.3
1071	24	1056	10	99	6.0
1064	20	1064	12	100	5.0
1063	20	1061	10	100	6.9
1068	22	1058	11	99	3.9
1059	22	1066	11	101	5.8
1059	21	1043	11	98	7.1
1068	20	1082	12	101	2.7
1070	20	1055	12	99	6.9
1057	20	1070	12	101	4.1
1058	23	1056	13	100	4.8
1069	23	1069	11	100	3.9
1065	20	1070	12	100	4.8
1062	20	1055	12	99	3.8
1074	24	1053	15	98	4.2
1053	21	1053	13	100	6.0
1068	23	1053	12	99	5.0
1059	21	1072	11	101	4.0
1069	19	1070	12	100	2.8
1058	21	1055	11	100	3.9
1069	21	1055	11	99	3.3
1058	20	1070	12	101	4.7
1055	20	1062	12	101	3.1
1072	21	1063	13	99	3.3
1042	21	1063	13	102	5.6
1084	20	1062	12	98	3.9
1064	22	1065	12	100	4.2
1063	22	1060	12	100	3.6
1058	21	1061	12	100	5.0
1069	21	1063	13	99	5.4
1085	23	1064	10	98	5.6
1041	23	1061	9	102	4.3
1061	22	1058	10	100	6.4
1066	21	1067	11	100	7.0
1058	21	1069	11	101	7.3
1069	22	1056	11	99	4.0
1056	20	1065	11	101	5.3
1071	21	1060	11	99	8.0
1078	20	1071	12	99	4.1
1049	20	1053	12	100	1.0
1066	21	1061	11	100	2.9
1061	18	1064	12	100	6.1
1063	21	1059	11	100	6.8
1064	21	1066	12	100	7.2
1066	21	1068	10	100	7.6
1061	20	1057	11	100	6.7
1058	20	1078	11	102	4.0
1069	21	1047	10	98	3.0
1063	21	1060	12	100	4.3
1064	21	1065	15	100	3.9
1065	22	1061	11	100	5.7
1062	21	1064	10	100	4.1
1075	19	1072	12	100	3.5
1052	20	1053	12	100	2.3
1077	21	1056	13	98	5.3
1050	22	1068	12	102	4.6
1068	19	1077	10	101	3.8
1059	20	1048	11	99	5.0
1064	22	1062	12	100	4.0
1056	21	1044	11	99	4.5
1071	19	1081	11	101	7.4

Analysis No.	Pb	Th	U	U-Pb Ratios						
	ppm	ppm	ppm	$^{207}Pb/^{206}Pb$	1σ	$^{207}Pb/^{235}U$	1σ	$^{206}Pb/^{238}U$	1σ	rho
JUL18B47	16.1	27.5	73.7	0.0759	0.0025	1.8501	0.0558	0.1772	0.0020	0.04
JUL18B48	16.5	27.6	74.3	0.0738	0.0022	1.8503	0.0545	0.1811	0.0021	0.16
JUL18B64	16.4	27.6	74.4	0.0751	0.0023	1.8489	0.0560	0.1791	0.0018	0.06
JUL18B65	16.9	28.1	76.0	0.0747	0.0023	1.8515	0.0563	0.1792	0.0018	0.17
Average (±2SD)	16	26	74							
N										
Wiendenbeck et al. (1995)										
Szymanowski et al. (2018)										
Standard zircon GJ-1 as an unknown										
AUG22A04	28.7	7.6	259.1	0.0579	0.0016	0.7834	0.0220	0.0979	0.0009	0.33
AUG22A05	28.4	7.8	259.6	0.0596	0.0016	0.7968	0.0217	0.0969	0.0008	0.30
AUG22A06	28.5	7.9	262.8	0.0582	0.0016	0.7776	0.0212	0.0966	0.0008	0.30
AUG22A69	28.1	7.5	253.9	0.0601	0.0014	0.8235	0.0195	0.0985	0.0010	0.41
AUG22A70	28.8	7.9	263.8	0.0611	0.0014	0.8311	0.0197	0.0978	0.0011	0.46
AUG22A71	29.7	8.1	273.1	0.0607	0.0016	0.8242	0.0213	0.0976	0.0011	0.42
AUG22B04	29.2	8.1	275.0	0.0583	0.0016	0.7693	0.0211	0.0954	0.0010	0.39
AUG22B05	30.6	8.4	285.7	0.0591	0.0015	0.7852	0.0201	0.0964	0.0009	0.38
AUG22B06	30.4	8.3	282.1	0.0593	0.0015	0.7918	0.0193	0.0966	0.0009	0.39
AUG22B94	30.1	8.2	275.8	0.0576	0.0014	0.7811	0.0189	0.0979	0.0009	0.36
AUG22B95	30.4	8.3	276.6	0.0589	0.0014	0.8041	0.0196	0.0986	0.0009	0.37
AUG22B96	29.5	8.1	269.8	0.0571	0.0016	0.7714	0.0209	0.0978	0.0009	0.34
JUL17A04	32.1	9.4	301.6	0.0609	0.0016	0.8238	0.0216	0.0977	0.0009	0.12
JUL17A05	32.7	9.5	305.5	0.0584	0.0016	0.7981	0.0211	0.0985	0.0008	0.12
JUL17A06	32.4	9.6	302.8	0.0589	0.0015	0.8004	0.0205	0.0981	0.0008	0.12
JUL17A27	33.0	9.8	307.9	0.0587	0.0015	0.7923	0.0199	0.0971	0.0009	0.12
JUL17A28	32.3	9.7	301.1	0.0594	0.0014	0.8071	0.0192	0.0978	0.0009	0.12
JUL17A49	31.9	9.4	298.1	0.0593	0.0016	0.8094	0.0208	0.0985	0.0009	0.08
JUL17A50	32.1	9.4	297.6	0.0606	0.0016	0.8314	0.0218	0.0987	0.0008	0.13
JUL17A71	32.0	9.7	298.4	0.0598	0.0015	0.8084	0.0199	0.0969	0.0010	0.14
JUL17A72	32.3	9.6	302.3	0.0586	0.0015	0.7924	0.0194	0.0971	0.0010	0.17
JUL17A86	32.1	9.4	297.4	0.0588	0.0014	0.8041	0.0197	0.0981	0.0008	0.26
JUL17A87	32.0	9.2	297.6	0.0596	0.0014	0.8105	0.0196	0.0974	0.0008	0.22
JUL17A88	32.4	9.4	297.4	0.0597	0.0014	0.8172	0.0189	0.0986	0.0009	0.07
JUL18B04	30.1	8.8	278.0	0.0597	0.0016	0.8005	0.0207	0.0971	0.0010	0.00
JUL18B05	30.2	9.0	278.6	0.0605	0.0016	0.8212	0.0205	0.0978	0.0009	0.01
JUL18B06	30.2	8.9	277.4	0.0596	0.0016	0.8104	0.0204	0.0981	0.0009	0.12
JUL18B27	31.1	8.7	283.9	0.0592	0.0018	0.8073	0.0231	0.0987	0.0010	0.03
JUL18B28	31.3	9.0	284.7	0.0594	0.0018	0.8118	0.0228	0.0988	0.0010	0.00
JUL18B61	31.8	9.2	293.8	0.0590	0.0015	0.7891	0.0187	0.0968	0.0008	0.05
JUL18B62	31.6	9.2	292.1	0.0584	0.0016	0.7848	0.0208	0.0969	0.0008	0.19
JUL18B63	31.5	9.0	291.9	0.0587	0.0016	0.7829	0.0202	0.0966	0.0008	0.06
Average (±2SD)										
N										
Jackson et al. (2004)										
Piazolo et al. (2017)										

* Concordance = 100% * ($^{206}Pb/^{238}U$ age)/ ($^{207}Pb/^{235}U$ age).

U-Pb Ages (Ma)					Ti
$^{207}Pb/^{235}U$	1σ	$^{206}Pb/^{238}U$	1σ	Conc.% *	ppm
1063	20	1052	11	99	3.3
1064	19	1073	11	101	6.8
1063	20	1062	10	100	6.4
1064	20	1063	10	100	4.1
1063±16		1062±16			4.8±3
65		65			65
		1062.4±0.4			
					4.73±0.32
587	13	602	5	103	4.3
595	12	596	5	100	3.4
584	12	594	5	102	3.2
610	11	606	6	99	4.9
614	11	601	6	98	2.6
610	12	600	6	98	2.3
579	12	587	6	101	5.5
588	11	593	5	101	3.8
592	11	595	5	100	4.1
586	11	602	5	103	5.8
599	11	606	5	101	2.8
581	12	601	5	104	1.8
610	12	601	5	98	6.5
596	12	606	5	102	6.6
597	12	604	5	101	8.1
592	11	598	5	101	
601	11	601	5	100	6.1
602	12	606	5	101	5.0
614	12	607	5	99	
602	11	596	6	99	2.9
593	11	597	6	101	3.9
599	11	603	5	101	4.2
603	11	599	5	99	6.3
606	11	606	5	100	3.5
597	12	598	6	100	2.6
609	11	602	5	99	2.2
603	11	603	5	100	5.1
601	13	606	6	101	5.5
603	13	607	6	101	6.3
591	11	596	5	101	4.6
588	12	596	5	101	2.5
587	12	594	5	101	4.3
598±19		600±10			4.4±3.2
32		32			30
601.6±3.8		599.8±4.8			
					3.4±0.4

Table S9

Summary of the operating parameters for MC-ICP-MS and the femto-second laser ablation system.

MC-ICP-MS (Neptune Plus)							
Farady cup configuration	L4	L3	L2	L1	C	H1	H2
	$^{89}Y^{+}$	$^{90}Zr^{+}$	$^{91}Zr^{+}$	$^{92}Zr^{+}$	$^{94}Zr^{+}$	$^{95}Mo^{+}$	$^{96}Zr^{+}$
RF power	1250 W						
Cool gas flow	16.0 L min^{-1}						
Auxiliary gas flow	0.80 L min^{-1}						
Argon make-up gas flow	~0.70 L min^{-1}						
Helium carrier gas flow	~0.60 L min^{-1}						
Nitrogen gas flow	8 ml min^{-1}						
Interface cones	X skimmer cone + Jet sample cone						
Mass resolution	Low						
Block number	1						
Cycles of each block	120						
Integration time (s)	0.524 s						
Laser ablation system (NWRfemto)							
Laser type	Yb femtosecond laser						
Wavelength	257 nm						
Pulse length	15 ns						
Energy density	1.2 J cm^{-2}						
Output energy	70%						
Ablation mode	Single spot						
Spot size	20 µm						
Laser frequency	1 Hz						
